# Post-glacial phylogeography and evolution of a wide-ranging highly-exploited keystone forest tree, eastern white pine (*Pinus strobus*) in North America: single refugium, multiple routes

**DOI:** 10.1186/s12862-016-0624-1

**Published:** 2016-03-02

**Authors:** John W. R. Zinck, Om P. Rajora

**Affiliations:** Faculty of Forestry and Environmental Management, University of New Brunswick, 28 Dineen Drive, Fredericton, NB E3B 5A3 Canada; Present address: Athletigen Technologies Inc., 535 Legget Drive, Kanata, ON K2K 3B8 Canada

**Keywords:** Phylogeography, Population genetic structure, post-glacial migration, *Pinus strobus*, Genetic signatures of heavy exploitation, Molecular evolution, Evolutionarily significant units

## Abstract

**Background:**

Knowledge of the historical distribution and postglacial phylogeography and evolution of a species is important to better understand its current distribution and population structure and potential fate in the future, especially under climate change conditions, and conservation of its genetic resources. We have addressed this issue in a wide-ranging and heavily exploited keystone forest tree species of eastern North America, eastern white pine (*Pinus strobus*). We examined the range-wide population genetic structure, tested various hypothetical population history and evolutionary scenarios and inferred the location of glacial refugium and post-glacial recolonization routes. Our hypothesis was that eastern white pine survived in a single glacial refugium and expanded through multiple post-glacial recolonization routes.

**Results:**

We studied the range-wide genetic diversity and population structure of 33 eastern white pine populations using 12 nuclear and 3 chloroplast microsatellite DNA markers. We used Approximate Bayesian Computation approach to test various evolutionary scenarios. We observed high levels of genetic diversity, and significant genetic differentiation (*F*_ST_ = 0.104) and population structure among eastern white pine populations across its range. A south to north trend of declining genetic diversity existed, consistent with repeated founder effects during post-glaciation migration northwards. We observed broad consensus from nuclear and chloroplast genetic markers supporting the presence of two main post-glacial recolonization routes that originated from a single southern refugium in the mid-Atlantic plain. One route gave rise to populations at the western margin of the species’ range in Minnesota and western Ontario. The second route gave rise to central-eastern populations, which branched into two subgroups: central and eastern. We observed minimal sharing of chloroplast haplotypes between recolonization routes but there was evidence of admixture between the western and west-central populations.

**Conclusions:**

Our study reveals a single southern refugium, two recolonization routes and three genetically distinguishable lineages in eastern white pine that we suggest to be treated as separate Evolutionarily Significant Units. Like many wide-ranging North American species, eastern white pine retains the genetic signatures of post-glacial recolonization and evolution, and its contemporary population genetic structure reflects not just the modern distribution and effects of heavy exploitation but also routes northward from its glacial refugium.

**Electronic supplementary material:**

The online version of this article (doi:10.1186/s12862-016-0624-1) contains supplementary material, which is available to authorized users.

## Background

Ecological changes and anthropogenic activities over the past 12,000 years have had a profound effect on the distribution of plant populations including that of forest trees in eastern North America [1]. Following the last glacial maximum (LGM, 26,500-19,000 ybp), a time when plant species were forced to inhabit unglaciated areas, climate oscillations and topographic and hydrological barriers have influenced the location of suitable habitats and the migration of plant populations [2]. Superimposed on phylogeographic patterns triggered by postglacial climatic changes is the impact of recent human disturbance and habitat change [3]. Therefore, knowledge of the historical distribution and postglacial phylogeography, evolution and expansion of a species is important to better understand its current distribution and population structure, the historical processes that shaped its current distribution and to predict potential fate in the future, especially under climate change conditions, as well as conservation and management of its genetic resources. However, these aspects for eastern North American plant species, especially forest trees, are not well understood.

In eastern North America (unlike Europe, with its east–west mountain ranges), the absence of major geographic barriers to northward dispersal can lead to a presumption that northward post-glacial recolonization simply proceeded uniformly across the longitudinal range of the recolonizing species. Thus the only phylogeographic patterns likely to be introduced during post-glacial recolonization would be south-to-north declines in genetic diversity introduced by repeated founder events [4]. However, applications of molecular genetic markers have shown that this view is far too simple, and that instead the landscape exposed by glacial retreat likely directed recolonization in ways that also introduced longitudinal structure in modern populations [5]. For tree species with ranges that extend from the Atlantic Ocean to the Midwest plains, two geographical features may have disrupted uniform post-glacial northward migrations: the Appalachian Mountains and the Great Lakes. In a few eastern and transcontinental North American conifer tree species, whose post-glacial phylogeography has recently been studied, refugia and post-glacial migration routes were inferred to be separated by the Appalachian Mountains. Three refugia (Beringian, Mississippian and east Appalachian) have been reported for transcontinental white spruce (*Picea glauca*) [6, 7], three southern and one northern refugia for transcontinental black spruce (*Picea mariana*) [8], and three (one east and one west of Appalachians and one in northern Canada) for widely-distributed almost-transcontinental jack pine (*Pinus banksiana*) [9]. For Atlantic white cypress (*Chamaecyparis thyoides*), Mylecraine et al. [10] argued that refugia and recolonization routes were separated by the Appalachian Mountains.

The objective of this study was to examine the range-wide population genetic structure and phylogeography, and infer post-glacial migration and evolution of eastern white pine (*Pinus strobus*) in North America in order to understand how historical processes have shaped the current species’ distribution and population genetic structure. We have chosen eastern white pine because (1) it is a widely distributed, ecologically and economically important key-stone species in eastern North America, (2) it is predicted that this species will expand its range northward under the anticipated climate change, (3) there is no information on range-wide population genetic structure and phylogeography of this species, and its post-glacial migration and evolution is not well-understood, and (4) it is of conservation genetic concerns because it has been heavily exploited for over 150 years [11]. Fossil-based studies, using radio-carbon dating, of eastern white pine historical distribution provided some indication that the species inhabited a glacial refugium in the mid-Atlantic plain of the eastern North American seaboard (Virginia and North Carolina) [12]. After the LGM, northward expansion continued to within 320 km of James Bay, northern Ontario, in part due to warmer-than-modern climates approximately 6000 year ago [13]. In Minnesota, contraction to the current range is estimated to have occurred about roughly 1000 years ago [14]. However, due to many inherent limitations, the fossil pollen data alone does not provide a clear and detailed picture of phylogeography and post-glacial migration and evolution of a species. Therefore, it is essential to examine a species’ phylogeography using molecular genetic markers.

Here we have examined range-wide population genetic structure and phylogeography of eastern white pine, using microsatellite DNA markers from the nuclear and chloroplast genomes, and inferred its post-glaciation migration and evolution using this genetic data supplemented with previously published fossil pollen information. We tested the following hypotheses: (i) if the current eastern white pine arose from a southern refugium, there should be a geographical trend of south to north decreasing genetic diversity as a result of repeated founder effect; (ii) that current eastern white pine populations descended from a single glacial refugium during the LGM and multiple post-glacial recolonization routes; and (iii) the Appalachian mountains and Great Lakes separated the post-glacial colonization routes resulting in longitudinal genetic differentiation of eastern white pine in the north. We take advantage of the fact that the chloroplast DNA is paternally inherited and thus pollen dispersed in conifers, e.g., [15], and the nuclear DNA is biparentally inherited and thus both pollen and seed dispersed. A combination of the nuclear and chloroplast microsatellite markers, as applied in [16], provides stronger inferences than based on either of these markers. We demonstrate that eastern white pine populations are highly genetically variable and significantly differentiated across the species’ range. Then we infer that eastern white pine survived in a single southern refugium and expanded northward using two major post-glacial migration routes, one of them further branched into two. The genetic signatures of extensive harvesting of eastern white pine over the past century and half on the contemporary genetic structure tended to blur the genetic signals of postglacial phylogeography and evolution that we disentangled.

## Methods

### Study species

Eastern white pine is a long-lived widely distributed species in eastern North America from Newfoundland in the east to southeastern Manitoba in the west and to parts of Georgia and South Carolina in the southeast [17]. It is highly ecologically and economically important and a keystone species of temperate white pine ecosystems [17]. Eastern white pine has undergone heavy exploitation over more than 150 years [11]) and multiple episodes of post-glacial range expansion and retraction [18]. The range-wide population genetic structure of this species remains poorly understood, and current genetic diversity and population structure estimates are based on studies covering only quite small geographic areas in the northern parts of the species’ range [19–27]. These studies have indicated that eastern white pine populations have moderate to high levels of genetic diversity and low levels of genetic differentiation. So far, there is no range-wide study reported on molecular population genetic diversity and structure in this species. Eastern white pine is a predominantly outcrossing species [28].

### Populations and sampling

**Fig. 1:**
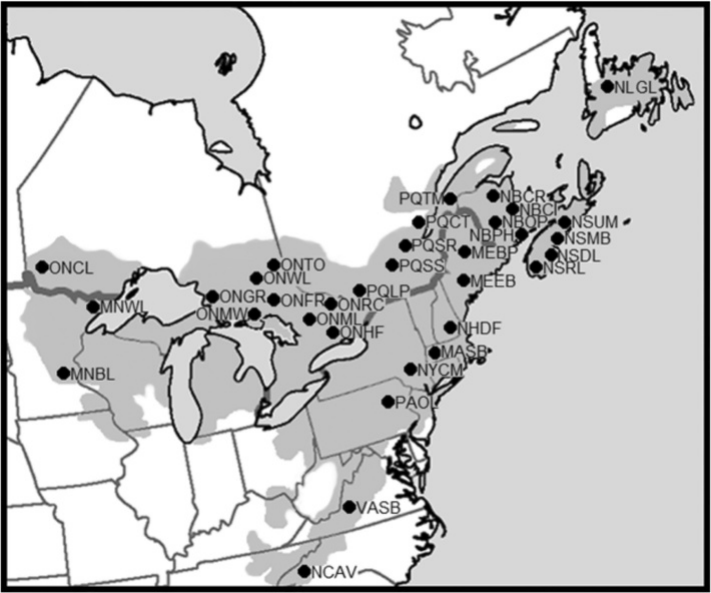
Location map of 33 eastern white pine populations sampled throughout the species’ range in North America. The species’ range in represented by the shaded region on land. The full names of the populations are provided in Table 1

Thirty-three natural eastern white pine populations were sampled from throughout the species’ range (Table 1, Fig. 1). Fifty mature eastern white pine trees were sampled randomly from each population. To minimize the chance of sampling siblings, we left a 30 m buffer between the sampled trees. We collected needles from each of the individual 1650 mature trees sampled from 33 populations. After collection, each needle sample was stored in a sealed plastic bag, with a 5 g silica desiccant pack, at −20 °C pending DNA extraction.

**Table 1:** Eastern white pine populations sampled, and their locations, abbreviated names and geographical coordinates

Province/State	Population	Abbreviation	Latitude (N)	Longitude (W)
Newfoundland	Grand Lake	NLGL	49°15'44.44"	56°53'24.24"
Nova Scotia	Saint Margarets Bay	NSMB	44°38'22.15"	63°52'5.49"
	Lake Rossignol	NSRL	44°16'23.40"	65° 8'32.22"
	Dory Mills Lake	NSDL	44°30'15.91"	64°24'35.47"
	Mount Uniacke	NSUM	44°57'15.77"	63°36'31.47"
New Brunswick	Paper Mill Hill	NBPM	45°47'9.55"	65°17'44.53"
	Canaan River	NBCI	46° 8'45.23"	65°35'27.39"
	Chipman Road	NBCR	46°19'14.24"	65°55'45.75"
	Odell Park	NBOP	45°57'21.66"	66°39'53.18"
Quebec	Temiscouata	QCTM	47°42'2.86"	68°51'41.74"
	Cap Tourmente	QCCT	47° 4'36.32"	70°48'16.28"
	Saint Renyold	QCSR	46°51'4.40"	71°48.900"
	Saint Stanilis	QCSS	46°38'35.28"	72°17'12.18"
	Lac Phillip	QCLP	45°33'44.63"	75°54'34.79"
Ontario	Muskoka	ONML	45° 1'12.60"	79°39'37.01"
	French River	ONFR	46° 3'8.24"	80°17'3.33"
	High Falls	ONHF	44°35'50.52"	78° 4'47.58"
	Goulais River	ONGR	46°44'57.21"	84°13'19.78"
	Whitefish Reserve	ONMW	46° 5'13.02"	81°43'23.63”
	Renfrew County	ONRC	45°39'49.07"	77°23'46.03"
	Wolf Lake	ONWL	46°50'35.55"	80°39'10.31"
	Timiskaming	ONTO	47° 7'50.58"	79°28'42.18"
	Crow Lake	ONCL	49° 5'3.56"	94°16'23.77"
Minnesota	Whale Lake	MNWL	47°51'53.88"	90°27'50.57"
	Boot Lake	MNBL	45°19'45.20"	93°07'39.90"
Maine	Etna Brook	MEEB	44°47'24.73"	69° 9'52.54"
	Baxter State Park	MEBP	45°40'21.15"	68°38'7.85"
New Hampshire	Deerfield	NHDF	43° 6'32.54"	71°15'40.85"
Massachusetts	Stockbridge	MASB	42°15'49.08"	73°17'9.84"
New York	Pacama Catskills	NYCM	41°56'47.40"	74°10'4.94"
Pennsylvania	Pocono Lake	PAOL	41° 5'42.83"	75°30'14.47”
Virginia	Bennett Springs	VABS	37°22'48.13"	80° 1'0.34"
North Carolina	Asheville	NCAV	35°36'59.03"	82°31'54.63"

### DNA extraction and microsatellite genotyping

We isolated total genomic DNA from 300 mg of ground needles from each individual using a modified CTAB method [29]. After extraction, the DNA was diluted to a 10 mM working concentration and stored in double distilled water at −20 °C. Twelve nuclear microsatellite markers (*RPS1b, RPS2, RPS12, RPS20, RPS25, RPS34b, RPS39, RPS50, RPS6, RPS118b, RPS119* and *RPS127*) [30] were used to genotype all sampled individuals and three chloroplast microsatellite markers *(pt26081, pt63718, pt71936*) [31] to genotype a subset of 20 individuals per population. The nuclear microsatellite markers were the same as used previously by Rajora et al. [22] in eastern white pine from Ontario. The chloroplast microsatellite markers (cpSSRs) were those that provided the best and unambiguous patterns in eastern white pine, selected from screening the cpSSRs developed earlier for *Pinus thunbergii* [31]. We used a subset of 20 individuals per population for cpSSR genotyping because the chloroplast genome is quite conserved; thus using a larger sample size will unlikely improve the results and inferences significantly. This sample size is consistent with or larger than that generally used in similar studies.

For each microsatellite marker, we modified one of the two primers to accept a fluorescently labelled m13 tail (700 nm or 800 nm wavelength). Polymerase chain reaction (PCR) reactions for the microsatellite amplification were performed in a volume of 10 μL. The reaction mixtures contained 10–15 ng of DNA, 2 μL 5x PCR Buffer (Promega, Madison, WI, USA), 0.5 μL 2 mM MgCl_2_ (EMD), 1 μL 4 mM dNTPs, 0.1 μL 10 mM untailed primer, 0.1 μL 10 mM fluorescently labelled m13 primers, 0.06 μL 10 mM m13 tailed primer, 0.1 μL 5 U/μL Taq DNA polymerase and 5.09 μL nuclease free water.

We performed PCR amplifications starting with a “touchdown” step from 65 °C to the annealing temperatures according to Echt et al. [30] and Rajora et al. [22], 27 cycles of 94 °C 30 s, annealing temperature 30 s and 72 °C 30 s, and an extension step at 72 °C for 3 min. PCR reactions were performed using 96 well EP Gradient S Master Cyclers (Eppendorf, Germany). To assist with genotyping calibration and to monitor possible PCR errors, we included a positive (previously tested working sample) and a negative (PCR master mix with no DNA) control in each PCR plate.

We used Licor Biosciences IR4300 DNA analyzers (Licor, Lincoln, Nebraska, USA) to visualize microsatellite polymorphisms through a 6 % agarose gel matrix (National Diagnostic Ureagel-6), suspended in a TBE buffer. To determine microsatellite fragment lengths, five LiCor 50–350 base pair molecular weight size standards were included in each gel. We used Licor Biosciences Saga Generation 2 (v3.3, Lincoln Nebraska, USA) to score microsatellite genotype data, which was verified manually. The nuclear microsatellite genotype data was scored as allelic constitution of individuals at a locus. The chloroplast microsatellite data was first scored as allelic constitution of individuals at individual three loci, and then as multilocus haplotypes to account for linkage between the chloroplast regions.

### Data analysis

#### Genetic diversity

Genetic diversity parameters of individual populations for the nuclear and chloroplast microsatellites were determined using GenAlEx 6 [32]. Number of alleles per locus (A_N_), number of private alleles (A_P_), and observed and expected heterozygosity (H_O_ and H_E_) were calculated for the nuclear markers. Number of alleles per locus (A_N_), Shannon’s Information Index (I) see [33], haplotype diversity (H), and unbiased haplotype diversity (uH) were calculated for the chloroplast markers. We also estimated the effective number of alleles (A_E_) per locus, rarefaction-based allelic richness (A_R_), and inbreeding index (*F*_IS_) for the nuclear microsatellites using FSTAT v2.9.3.2 [34]. Departures from Hardy-Weinberg equilibrium were examined. We tested non-random association of alleles at different nuclear loci using a linkage disequilibrium test in FSTAT v2.9.3.2 [34] and Arlequin v3.5.1.2 [35]. We calculated correlation between latitude and genetic diversity indices of populations to test for any declining genetic diversity trend from south to north, an expected signature of founder effects along the recolonization route(s).

#### Population genetic differentiation and structure

Inter-population genetic differentiation for nuclear microsatellites was determined by using *F*-statistics [36] employing GenAlEx 6 [32], and AMOVA [37] using Arlequin v3.5 [35]. G_ST_ and R_ST_/N_ST_ among populations were calculated from the chloroplast markers using 1000 permutations in PermutCpSSR v2.0 [38].

The population genetic structure resulting from natural barriers and human activities was examined using two Bayesian model-based clustering approaches. First, STRUCTURE [39] was used to examine the range-wide population structure, based on the 12 nuclear microsatellites, under the assumption that sample locality has no significant role in population structure. STRUCTURE works by grouping individuals into clusters (*K*) such that Hardy-Weinberg equilibrium is maximized within clusters. By varying the *K*-values across several runs and inspecting the resulting probabilities for these various *K* values, one can infer the likely number of groups which best capture the variation present in the data. We performed multiple runs of STRUCTURE to test *K* values ranging from 1 to 33, over 50 replications, using an admixture model and correlated allele frequencies options [40], a 10^5^ burn-in length and 10^5^ MCMC replications for each run. In order to facilitate the selection of the best *K* value, we used STRUCTURE HARVESTER [41]; an online application that uses the Evanno et al. [42] technique for assessing and visualizing likelihood values across multiple values of *K* and detecting the number of genetic groups that best fit the data.

Due to the large variation in geographical distances among the locations of the sampled populations, we sought to disentangle any artifactual population structure signal caused by populations in close proximity. We did this by performing a second Bayesian population structure analysis using BAPS v5.3 [43]. Unlike STRUCTURE, BAPS provides the user with an option to integrate spatial coordinates into the prior assumptions [44]. We also employed the BAPS to examine the population structure as defined by the chloroplast markers, using the haplotype data. Both the nuclear and chloroplast datasets were analyzed for a maximum of 33 spatial cluster groups with a population mixture option.

Regions where abrupt genetic differentiation exists over relatively small geographic distances can be indicative of boundaries of population groups and genetic barriers in a species range perhaps where distinct phylogeographic lineages meet. We used Barrier v2.2 [45] to identify genetic barriers and boundaries of population groups, for nuclear and chloroplast microsatellites data, using both the multi-locus pairwise *F*_ST_ matrix and individual locus *F*_ST_ pairwise matrices to determine the number of loci that support any inferred barriers.

#### Phylogeographical analysis

Although we observed varying levels of population differentiation and a significant magnitude of population structure among eastern white pine populations, signals of past phylogeographic patterns were present in nearly all analyses (e.g. regional clustering in low *K*-value STRUCTURE runs, Barrier analysis). In order to disentangle these patterns from the present population structure, we employed geographic distribution patterns of chloroplast haplotypes, and tested various phylogeographic hypotheses using the Approximate Bayesian Computation (ABC) analysis.

We first examined the composition and geographic distribution of chloroplast haplotypes to infer genetic lineages and post-glacial northward migration of eastern white pine. The geographic distribution of the haplotype data was visualized using PhyloGeoViz [46]. The distribution of these haplotypes across the species’ range was combined with previous information on fossil pollen occurrence [12] to formulate possible recolonization scenarios, including possible routes and divergence times.

We used DIYABC v2.0.3 [47] to test competing hypothetical scenarios regarding phylogeography and population divergence in eastern white pine on a range-wide scale. The hypotheses were constructed primarily to test the order (from south to north) and time of divergence of the population groups, as well as the possibility of population admixture after divergence. For the ABC simulations, we analyzed the nuclear and chloroplast marker data separately. We hypothesized four groups of populations (lineages) based on the signals from STRUCTURE, BAPS and Barrier analyses and geographical distribution of chloroplast haplotypes (see Results). These groups were as follows: Western, Central, Eastern and Southern (Additional file 1: Table S1). First, we compared the competing scenarios of population divergence without admixture and then with population admixture (Additional file 2: Figure S1). The information on the parameters and their prior distributions used in the analysis are provided in Additional file 1: Table S1. Then we compared the best scenarios taken from each of the without and with admixture analyses. We simulated one million data sets for each of the scenarios, and four million data sets for the comparison between the two best scenarios (~ two million each). The population divergence scenarios differed in the order of population divergence and in the number and time of demographic expansion events. The population admixture scenarios were developed based on both the chloroplast haplotype distribution and the best scenario from with and without admixture comparison.

We performed a logistic regression to estimate posterior probability of each scenario, taking the simulated data sets closest to our real data set between 0.1 % and 1 % [47]. The 95 % credibility intervals for the posterior probabilities were computed through the limiting distribution of the maximum likelihood estimators. Once the most likely scenarios were identified, we used a linear regression analysis to estimate the posterior distributions of parameters under this scenario. We chose the 1 % of the simulated data sets closest to our real data for the logistic regression after applying a logit transformation to the parameter values. In order to evaluate the goodness-of-fit of the estimation procedure, we performed a model checking computation [47] by generating 10,000 pseudo-observed data sets with parameters values drawn from the posterior distribution given the most likely scenario.

## Results

### Range-wide genetic diversity

A total of 340 alleles were observed at 12 nuclear microsatellite loci in 1650 eastern white pine individuals. Twenty alleles were observed at three chloroplast microsatellites in the subset of 660 individuals. The genetic diversity parameters and fixation index estimates for the studied eastern white pine populations based on nuclear microsatellites are in Table 2, whereas genetic diversity parameters based on chloroplast microsatellites are in Table 3. A total of 60 chloroplast haplotypes were observed (Additional file 3: Table S2). Five of these were most common (Table 4).

**Table 2:** Genetic diversity parameters and fixation index (*F*_IS_) for eastern white pine populations based on nuclear microsatellites

Populations	A_N_	A_R_	A_E_	A_P_	H_O_	H_E_	*F*_IS_
NLGL	11.17	11.08	5.39	0.00	0.67	0.78	0.15
NSMB	8.17	8.10	3.95	0.17	0.63	0.67	0.07
NSRL	8.67	8.59	4.29	0.25	0.59	0.70	0.16
NSDL	8.67	8.60	4.09	0.08	0.60	0.70	0.15
NSUM	8.92	8.84	3.97	0.25	0.63	0.71	0.13
NBPM	10.92	10.80	4.54	0.00	0.73	0.75	0.04
NBCI	10.58	10.47	4.42	0.17	0.59	0.72	0.19
NBCR	10.67	10.58	5.05	0.00	0.64	0.76	0.16
NBOP	10.00	9.94	5.46	0.25	0.73	0.76	0.04
QCTM	10.25	10.18	4.81	0.17	0.70	0.77	0.11
QCCT	9.33	9.26	4.36	0.25	0.62	0.74	0.17
QCSR	10.25	10.14	4.17	0.00	0.56	0.70	0.20
QCSS	9.83	9.76	4.88	0.08	0.65	0.69	0.07
QCLP	9.17	9.09	4.05	0.00	0.63	0.70	0.12
ONML	10.33	10.24	5.21	0.08	0.70	0.71	0.03
ONFR	10.17	10.08	5.41	0.08	0.74	0.74	0.01
ONHF	10.67	10.57	4.69	0.00	0.62	0.72	0.15
ONGR	10.17	10.06	4.76	0.00	0.77	0.75	−0.01
ONMW	10.67	10.53	4.53	0.17	0.63	0.68	0.09
ONRC	9.17	9.08	4.23	0.33	0.59	0.75	0.22
ONWL	9.83	9.75	4.68	0.08	0.56	0.65	0.16
ONTO	9.83	9.75	5.14	0.08	0.80	0.75	−0.06
ONCL	10.08	10.03	5.88	0.00	0.84	0.80	−0.04
MNWL	10.08	10.00	4.98	0.08	0.77	0.77	0.00
MNBL	10.33	10.17	5.19	0.92	0.85	0.78	−0.08
MEEB	10.00	9.91	4.98	0.08	0.70	0.76	0.08
MEBP	10.58	10.50	5.38	0.00	0.66	0.76	0.14
NHDF	9.92	9.81	4.96	0.00	0.72	0.76	0.06
MASB	11.08	10.96	5.18	0.00	0.68	0.74	0.10
NYCM	13.33	13.17	6.26	0.00	0.74	0.81	0.10
PAOL	14.25	14.11	6.70	0.00	0.70	0.82	0.16
VABS	13.00	12.90	6.57	0.08	0.67	0.82	0.20
NCAV	15.25	14.99	6.60	0.25	0.66	0.82	0.21
Overall mean	10.47	10.36	4.99	0.12	0.68	0.74	0.10

A_N_, number of alleles per locus; A_R_, allelic richness A_E_, effective number of alleles per locus; A_P_, number of private alleles per locus; H_O_, observed heterozygosity; H_E_, expected heterozygosity; *F*_IS_, inbreeding (fixation) index. Detailed information on populations is provided in Table 1

**Table 3:** Genetic diversity parameters of eastern white pine populations based on chloroplast microsatellites

Populations	A_N_	I	H	uH
NLGL	3.33	0.70	0.37	0.39
NSMB	2.67	0.58	0.32	0.34
NSRL	3.00	0.66	0.37	0.39
NSDL	2.67	0.68	0.39	0.41
NSUM	2.67	0.63	0.36	0.38
NBPM	2.33	0.61	0.37	0.39
NBCI	2.33	0.55	0.32	0.34
NBCR	2.33	0.55	0.33	0.35
NBOP	2.67	0.58	0.33	0.34
QCTM	2.67	0.74	0.45	0.47
QCCT	2.67	0.84	0.53	0.55
QCSR	3.00	0.63	0.34	0.35
QCSS	2.67	0.53	0.31	0.32
QCLP	2.33	0.63	0.39	0.41
ONML	3.33	0.76	0.42	0.44
ONFR	3.33	0.89	0.50	0.52
ONHF	3.33	0.85	0.48	0.50
ONGR	3.67	0.94	0.52	0.55
ONMW	3.67	0.90	0.48	0.51
ONRC	3.67	0.83	0.45	0.47
ONWL	3.67	0.94	0.50	0.53
ONTO	3.33	0.93	0.53	0.56
ONCL	3.67	0.96	0.53	0.56
MNWL	4.33	1.11	0.61	0.64
MNBL	3.67	1.10	0.62	0.65
MEEB	3.00	0.78	0.44	0.47
MEBP	2.67	0.46	0.24	0.25
NHDF	3.00	0.77	0.44	0.46
MASB	3.00	0.93	0.57	0.60
NYCM	3.33	0.95	0.55	0.58
PAOL	3.33	0.92	0.52	0.55
VABS	4.00	1.01	0.56	0.59
NCAV	4.00	1.13	0.60	0.64
Overall mean	3.13	0.79	0.45	0.47

A_N_, number of alleles per locus; I, Shannon’s Information Index; H, haplotype diversity; uH, unbiased haplotype diversity. Detailed information on populations is provided in Table 1

**Table 4:** Allele composition of the most abundant chloroplast microsatellite haplotypes observed in eastern white pine populations

Chloroplast haplotype	Chloroplast microsatellite alleles		
	pt26081	pt63718	pt71936
Haplotype S	136	113	163
Haplotype V	136	114	163
Haplotype AG	136	116	161
Haplotype AJ	136	117	161
Haplotype AP	138	115	163

Allelic compositions of other chloroplast microsatellite haplotypes are provided in Additional file 3: Table S2

A geographic trend of decreasing genetic diversity from south to north was observed for both nuclear and chloroplast microsatellite markers (Tables 2 and 3). Populations in the southern portions of the eastern white pine range had higher A_N_, A_R_, A_E_ and heterozygosity than northern ones for nuclear markers (Table 2). Generally, the Asheville population from North Carolina showed the highest and the Saint Margarets Bay population from Nova Scotia the lowest nuclear microsatellite genetic diversity. Similar patterns were observed for the chloroplast microsatellite genetic diversity (Table 3). In general, populations in the northeast (New Hampshire-NH, Maine-ME, Massachusetts-MA, Nova Scotia-NS and New Brunswick-NB) had lower levels of average A_N_, A_R_, and A_E_ for nuclear markers and lower levels of average chloroplast haplotype diversity than the rest of the populations. The Newfoundland population GL showed somewhat higher levels of genetic diversity than the Nova Scotia and New Brunswick eastern white pine populations. Overall, the Nova Scotia populations had the lowest allelic diversity (Tables 2 and 3). Western populations (western Ontario and Minnesota) had, on average, slightly higher levels of heterozygosity at nuclear microsatellites. All of the genetic diversity parameters for the nuclear markers were inversely correlated with latitude: A_N_: *r* = −0.6699, *p* = 0.00002; A_R_: *r* = −0.6663, *p* = 0.00002; A_E_: *r* = −0.5308, *p* = 0.00148; H_O_: *r* = −0.1005, *p* = 0.5779; H_E_: *r* = -0.3895, *p* = 0.02506. Likewise all of the chloroplast microsatellite genetic diversity parameters were also negatively correlated with latitude: A_N_: *r* = −0.2411, *p* = 0.1765; I: *r* = −0.3318, *p* = 0.0592; H: *r* = −0.3207, *p* = 0.0688; uH: *r* = −0.3311, *p* = 0.05981. However, the number of private alleles did not show any such geographical patterns: latitude, *r* = 0.0458, *p* = 0.8002. The inbreeding coefficient (*F*_IS_) also showed a trend of decreasing from south to north for the nuclear microsatellite markers: *r* = −0.3738, *p* = 0.0326.

### Inter-population genetic differentiation

The overall mean *F*_ST_ among the populations was 0.104 from the nuclear microsatellite markers, and it was significantly higher than 0. Likewise, the AMOVA results revealed significant among-population variation of 10.38 %, which was consistent with the *F*_ST_ estimates. As expected for a gymnosperm forest tree species, the majority of genetic variation was explained by within-population variation (~90 %). The inter-population genetic differentiation from chloroplast microsatellites was lower than estimates from the nuclear markers (AMOVA = 6 %; G_ST_ = 0.035, R_ST_ = 0.045, N_ST_ = 0.075) but still significantly higher than 0.

### Population genetic structure

**Fig. 2:**
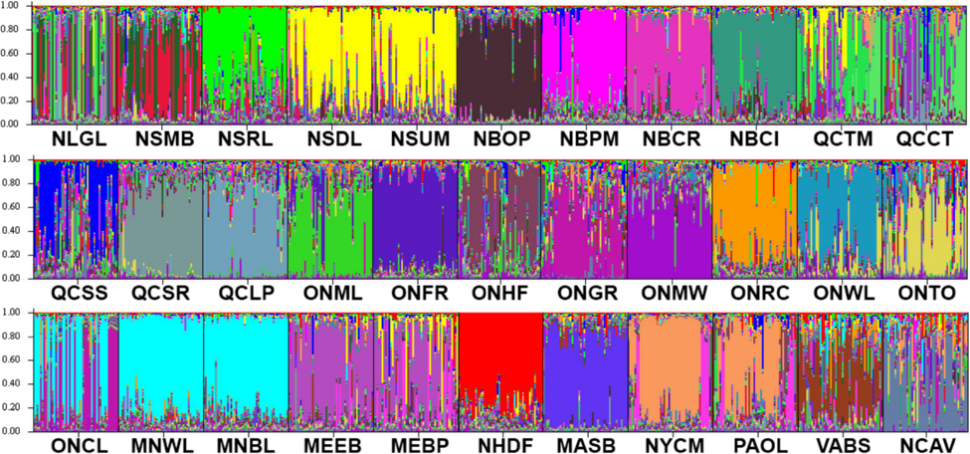
Summary bar plot of estimated membership coefficient (Q) of the studied individuals from 33 eastern white pine populations from STRUCTURE analysis for (*K* =) 30 clusters. Each individual is represented by a single vertical line while each colour represents one of the 30 clusters. The full names of the populations are provided in Table 1

The two Bayesian analyses of population genetic structure revealed significant levels of genetic structure of eastern white pine populations across its range, and the results were consistent between the two approaches with only slight differences. After performing Evanno et al. [42] adjustments in STRUCTURE HARVESTER [41], we observed a number of high *delta K* peaks, yet the most prominent was at *K* = 30 genetic groups (Additional file 4: Figure S2). As such, STRUCTURE revealed 30 genetic groups among 33 eastern white pine populations (Fig. 2). Two populations each from Nova Scotia (NSDL and NSUM), New Brunswick (NBPM and NBCR), and Minnesota (MNWL and MNBL) grouped together in the same group. Each of the rest of the 27 population formed its own individual group (Fig. 2). BAPS, with the addition of geographic coordinates, identified 26 genetic groups among 33 populations (Additional file 5: Figure S3). In all cases, populations that were clustered together were in close geographical proximity.

**Fig. 3:**
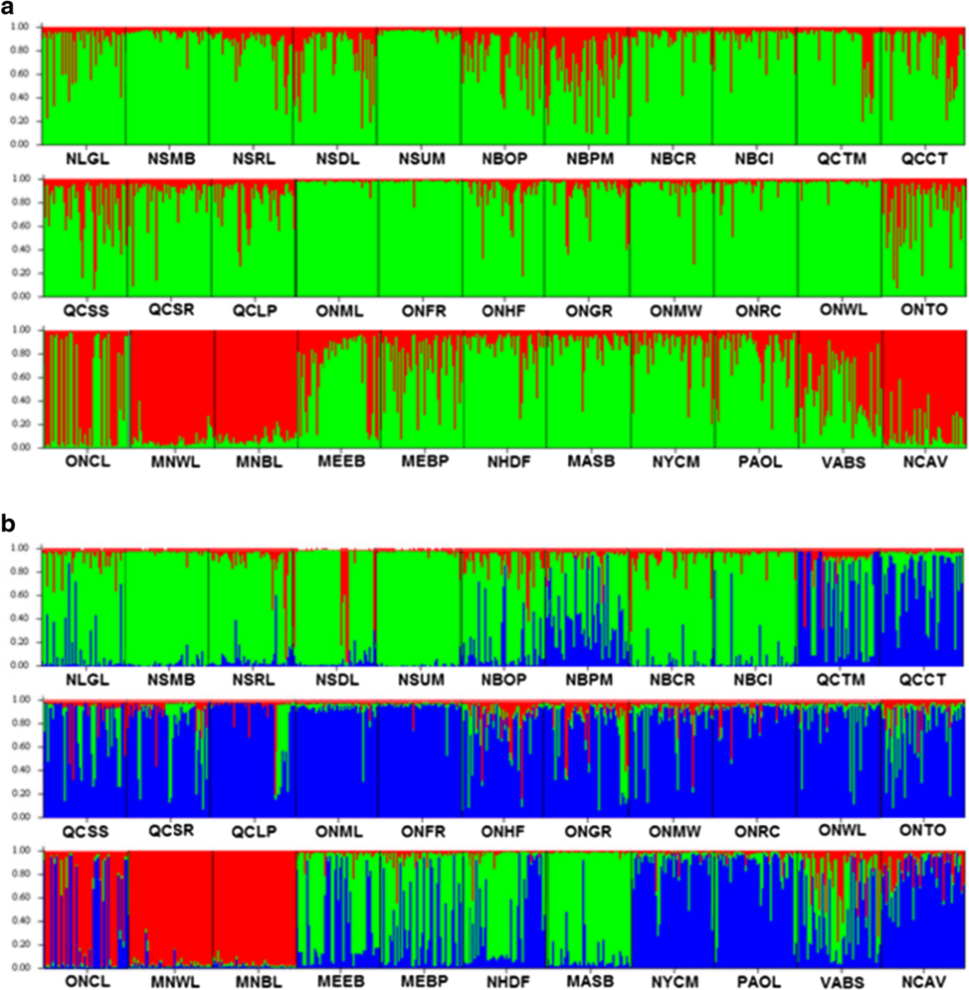
Summary bar plot of estimated membership coefficient (Q) of eastern white pine individuals from 33 populations from STRUCTURE analysis. Each individual is represented by a single vertical line. **a** *K* = 2: populations were clustered into a western (*red*) and a central/eastern (*green*) group. **b** *K* = 3: populations were clustered into a western (*red*), a central (*blue*) and an eastern (*green*) group, representing the three major phylogeographic lineages. The full names of the populations are provided in Table 1

Although STRUCTURE identified 30 distinct genetic groups (Fig. 2), when we examined the clustering of samples at lower *K* values, we observed what might be underlying phylogeographic patterns. For example, at *K* = 2, western populations (MNBL, MNWL, and ONCL) were clustered into a distinct group from the rest of the populations (Fig. 3a). At *K =* 3 an additional distinct division was observed between central samples (Pennsylvania- PA, New York-NY, Ontario-ON and Quebec-QC) and eastern samples (NH, ME, MA, NF, NS and NB) (Fig. 3b), resulting in three groups of populations. The fourth group consisted of the two southern populations from Virginia and North Carolina.

The results from the Barrier analysis were generally consistent with those of the STRUCTURE analysis with *K = 3*. We identified two barriers among the 33 sample populations from both the nuclear and chloroplast microsatellite markers that separated the populations into three groups (Additional file 5: Figure S3). The first barrier separated the 3 most western locations (ONCL, MNBL, and MNWL) from all others, and was supported by all 12 nuclear and 3 chloroplast microsatellite loci. The second barrier separated a central and southern (10 populations) from an eastern group (20 populations) and was supported by 10 nuclear and 3 chloroplast microsatellite loci.

The neighbour joining and maximum likelihood trees based on Nei’s genetic distances [48] or pairwise *F*_ST_ estimates generally supported the STRUCTURE *K* = 3 and barrier analyses results for three major groups among the 33 eastern white pine populations.

### Phylogeographic patterns

**Fig. 4:**
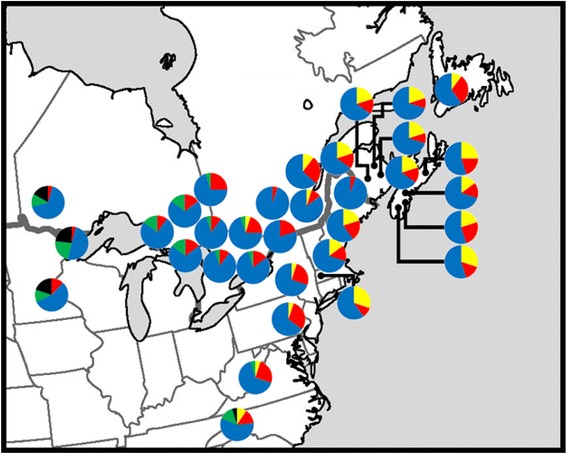
Geographic distribution of the most abundant chloroplast haplotypes in eastern white pine populations. Colours correspond to individual haplotype. Yellow, Haplotype S; Red, Haplotype V; Green, Haplotype AG; Black, Haplotype AJ; Blue, Haplotype AP. The allelic composition of the haplotype is provided in Table 4

The geographic distribution of the most common chloroplast haplotypes across the sampled range is presented in Fig. 4. The southernmost population NCAV from North Carolina had all five most common chloroplast microsatellite haplotypes, whereas the eastern populations had two or three of these chloroplast microsatellite haplotypes. From the haplotypes constitution and haplotypes sharing among populations, three phylogenetic lineages were apparent: western, central and eastern (Fig. 4). The Green (AG) haplotype was shared between the western and some central populations, indicating some sort of admixture between these apparent lineages.

**Fig. 5:**
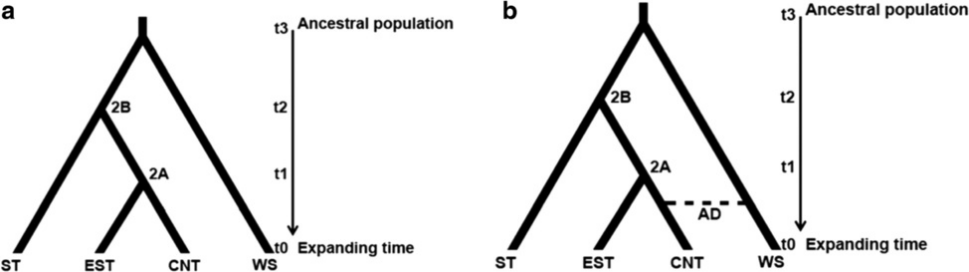
Highest probable ancestral connectivity observed using DIY Approximate Bayesian Computation (ABC) analysis between four population groups from **a** nuclear microsatellite, and **b** chloroplast microsatellite markers. Starting at t_0_ Group CNT and EST coalesced at t_1_ forming 2A; 2A coalesced with ST at t_2_ forming 2B; WS coalesced with 2B at t_3_. One admixture event was observed in the chloroplast data (AD, dash lines) between CNT and WS before t_1_. (ST: southern group. EST: eastern group. CNT: central group. WS: western group. t_0_-t_3_: divergence times. AD: admixture events. See Additional file 2: Table S1 for group information

The patterns of evolutionary divergence and phylogeographic lineages of eastern white pine populations revealed from the hypotheses testing using the ABC analysis were generally consistent between the nuclear and chloroplast markers (Table 5; Fig. 5). The best scenario of evolutionary divergence based on the highest posterior probabilities from the nuclear microsatellites was Sc1 (Table 5, Fig. 5a). This placed the first event (1-t_3_) of population divergence between the southern group (ST) and the western group (WS), the second split (1-t_2_) between the ST and the ancestral population of the central (CNT) and eastern (EST) groups, and the final split (1-t_1_) between the central and eastern groups (Fig. 5a). The best scenario of evolutionary divergence based on the highest posterior probabilities from the chloroplast microsatellites was Sc4 (Table 5; Fig. 5b), which is the same as observed from the nuclear microsatellite data but with the addition of an admixture event between the western and central groups (Table 5, Fig. 5b). The parameters (effective population size (N), divergence time in terms of the number of generations (t), and mutation rate (Mμ) estimated for the best evolutionary divergence scenarios are in Table 6, which showed similar patterns for the nuclear and chloroplast microsatellite data.

**Table 5:** Posterior probabilities for the hypothesized eastern white pine evolutionary scenarios from the ABC analysis

Evolutionary scenario	Nuclear microsatellites		Chloroplast microsatellites	
	Posterior probability of scenario	Confidence interval (95 %)	Posterior probability of scenario	Confidence interval (95 %)
Population divergence				
**Sc1**	**0.7145**	**[0.6226,0.8064]**	**0.6016**	**[0.5235,0.6696]**
Sc2	0.1810	[0.1119,0.2502]	0.3938	[0.3257,0.4619]
Sc3	0.1045	[0.0472,0.1617]	0.0046	[0.0001,0.0484]
Population admixture				
**Sc4**	**0.6667**	**[0.5467,0.7867]**	**0.5952**	**[0.4923,0.6981]**
Sc5	0.3333	[0.1736,0.5088]	0.4048	[0.2783,0.5289]
Best scenario				
**Sc1**	**0.5469**	**[0.4634,0.7145]**	0.4172	[0.1818,0.3997]
**Sc4**	0.4531	[0.2242,0.4487]	**0.5828**	**[0.4738,0.6840]**

Scenarios with the highest posterior probability for each test are represented in bold. The group information is provided in Additional file 1: Table S1 and the illustrations of the hypothesized scenarios are provided in the Additional file 2: Figure S1

**Table 6:** Parameter estimates for the best population divergence scenarios from the Approximate Bayesian Computation (ABC) analysis

Parameter	Nuclear markers (Sc1)			Chloroplast markers (Sc4)		
	Mean	q025	q975	Mean	q025	q975
N_WS_ (10^3^)	3.75	0.98	8.77	0.64	0.24	0.98
N_CNT_ (10^3^)	8.88	6.75	9.95	5.20	1.35	9.57
N_EST_ (10^3^)	7.76	4.64	9.82	2.66	0.62	7.54
N_ST_ (10^3^)	4.96	1.58	9.33	6.79	2.80	9.78
t_1_ (generations x10^2^)	1.08	0.30	2.29	0.63	0.09	2.26
t_2_ (generations x10^2^	2.56	0.78	6.97	1.97	0.36	5.96
t_3_ (generations x10^2^)	3.36	0.54	15.8	5.32	1.42	9.64
Mμ (10^−4^)	6.98	3.57	9.82	2.87	1.10	7.61

N: group effective populations size; t: divergent time; Mμ: mutation rate. Information on groups is provided in the text and Additional file 1: Table S1

## Discussion

In order to better understand the extant population genetic structure, the historical processes that shaped the current distribution and potential fate of a species in the future, especially under climate change conditions, and conservation of its genetic resources, knowledge of its postglacial phylogeography, evolution and expansion is important. Here we have examined the range-wide genetic diversity and population structure of widely distributed and heavily exploited keystone species, eastern white pine, using microsatellite markers of the nuclear and chloroplast genomes and inferred its postglacial evolution and migration testing various hypothetical evolutionary scenarios. We have demonstrated that the extant eastern white pine populations have relatively high genetic diversity, with south to north trend of reduced genetic diversity, and are significantly genetically structured across the range. The signals of postglacial phylogeography and evolution were disentangled from the effects of resource extraction of over the past century and half. Our results suggest a single southern refugium, two recolonization routes and three genetically distinguishable lineages in eastern white pine that we suggest be treated as separate Evolutionarily Significant Units.

### Population genetic diversity

Eastern white pine has relatively high nuclear microsatellite DNA genetic diversity over its range. We observed levels of nuclear microsatellite genetic diversity (allelic diversity and/or heterozygosity) in the sampled eastern white pine populations that were on average higher than the microsatellite diversity observed in studies of other widely distributed conifer species, including its sister species western white pine, *Pinus monticola* (A_N_ = 7.5, H_E_ = 0.67) [26], and lodgepole pine, *Pinus contorta* (A_N_ = 11.8, H_O_ = 0.46, H_E_ = 0.43 [49]. The observed nuclear microsatellite genetic diversity was also higher than that reported earlier for eastern white pine from Galloway Lake area, Ontario based on the same microsatellites (A_N_ = 9.4, H_O_ = 0.52, H_E_ = 0.60) [22], and Hartwick Pines State Park, Michigan (A_N_: 6.7, 7.3; H_O_: 0.47, 0.48; H_E_: 0.46, 0.49) [23], Menominee Reserve in Wisconsin (H_E_ = 0.49) [24] and in a study covering roughly one third of the species’ range in Canada (A_N_ = 6.6, H_O_ = 0.74 H_E_ = 0.80) [26] based on some of the same microsatellites. These observations of higher genetic diversity observed in our study is consistent with much larger range of eastern white pine studied in ours than previous studies. Also, our study included southern populations from Virginia and North Carolina that were found to be the most genetically diverse. The genetic diversity of the chloroplast microsatellites was in all cases lower than that for the nuclear markers. This is consistent with the lower mutation rate in chloroplast than nuclear microsatellites in *Pinus* [50] and other plants. Chloroplast microsatellites (cpSSR) have been previously used to test the somatic stability of the cloned material [51] and spatial genetic structure [52] in eastern white pine. This is the first report of chloroplast microsatellite genetic diversity across the range in eastern white pine. The haplotype diversity observed in our study is lower than that reported for four eastern white pine populations sampled from the Beaver Island Archipelago in Michigan (H = 0.80) [52]. The differences are likely due to the differences in the sample size and the number of cpSSRs used between the two studies. We used 20 individuals per population and three cpSSRs, whereas the average sample size in the Myers et al. [52] study was 78 and they used six cpSSRs with only one common between the two studies.

Our study clearly demonstrates the existence of south–north patterns in the genetic diversity levels, with the populations in Virginia and North Carolina having higher levels of genetic diversity than the northern populations. This is consistent with the possible repeated founder effects during post-glacial migration northward of eastern white pine from a southern Pleistocene refugium. The lower genetic diversity in the northern eastern white pine populations may also be due to divergent selection in response to south to north gradient in climate factors, such as temperature and moisture regimes, and range marginalization [27]. However, none of the microsatellite loci showed any signatures of divergent selection when we tested for outliers with respect to the magnitude of *F*_ST_ using BayeScan ver. 2.1 [53]. Somewhat higher genetic diversity in the Newfoundland population as compared to the New Brunswick and Nova Scotia populations may be due to its location in the Grand Lake Ecological Reserve (http://www.env.gov.nl.ca/env/publications/parks/little_grand_lake_web.pdf), where human impacts have been limited. On the other hand, the New Brunswick and Nova Scotia eastern white populations have been heavily exploited. The number of private alleles did not show any geographical patterns in our study. Private alleles may arise from population-specific new mutations and severely curtailed inter-population gene flow. Geographic patterns for private alleles will be expected if the mutation and gene flow rates were geographically structured among populations within a region: southern, northern, eastern central, and western. Apparently, this is not the case with the eastern white pine populations studied. However, a separate study is required to validate this assumption.

### Population genetic differentiation and structure

We observed 10.4 % interpopulation genetic differentiation based on the *F*_ST_ and AMOVA analyses, and 26 (BAPS) or 30 (STRUCTURE) groups of populations among the 33 eastern white pine populations. These results clearly suggest that significant population genetic structure and differentiation exist across the range of eastern white pine. The observed levels of genetic differentiation could be considered as low when compared to the plant kingdom as a whole but for the conifer trees, the levels are higher than the average of 0.073 (7.3 %) [54]. Significant inter-population genetic differentiation may be due to the reduction in population size and numbers and fragmentation resulting from heavy exploitation of this species over 150 years [11]. Mortality caused by invasive white pine blister rust (*Cronartium ribicola*) may have also reduced the population size and number of eastern white pine. Encroaching agriculture, grasslands and deciduous forests, and changing precipitation and wind patterns may negatively impact the distances over which seeds and pollen are dispersed between populations. All of the above factors may have reduced the levels of inter-population gene flow and increased inbreeding. However, eastern white pine has strong inbreeding depression [55], and selection against inbreds can occur at a very early stage in conifers [56]. Although eastern white pine is long-lived and has highly dispersed pollen [52]; these factors may not be enough to counterbalance the effects of anthropogenic and natural disturbances to sustain a homogenized genetic structure over its range.

The inter-population genetic differentiation of 10.4 % in our study is higher than that reported earlier for eastern white pine from its part of the Canadian range based on microsatellite (*F*_ST_ = 0.084) [26] and allozyme markers (*F*_ST_ = 0.061 [21], *F*_ST_ = 0.019 [19]). This may be the result of the large area covered by our study that, for the first time, included populations from the western and southern edges of the range. Within the smaller range, in particular the western populations, we observed similar levels of differentiation (*F*_ST_: 0.084; Phi variance: 0.071) as previously reported by Mehes et al. [26]. Chloroplast microsatellite genetic differentiation was lower than that for the nuclear markers. This is likely due to pollen-mediated paternal inheritance of the chloroplast genome in *Pinus* [15]) and long-distance gene dispersal via pollen as compared to that via seeds in conifers.

### Phylogeography and post-glacial evolution of eastern white pine

**Fig. 6:**
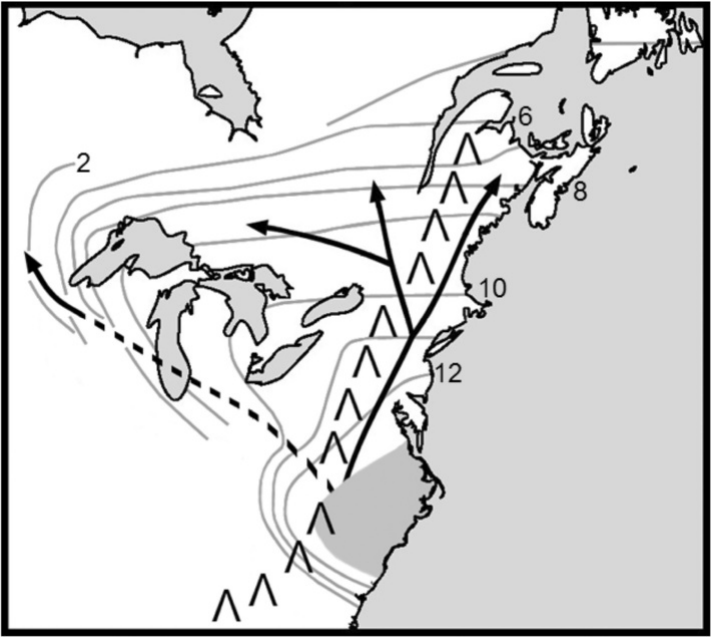
Probable eastern white pine post-glacial recolonization routes (arrows) from the glacial refuge (shaded grey area) based on the highest probable Approximate Bayesian Computation (ABC) scenarios observed from nuclear and chloroplast microsatellite data and available fossil pollen information. The Appalachian Mountain Range is shown by ˄. Dashed line indicates assumed route of colonization of western populations. The contour line represents approximate colonization time (x1000 ybp) based on fossil pollen and recolonization information from Davis [12]

The phylogeographic patterns emerged from the nuclear and chloroplast genetic markers were consistent between themselves and broadly consistent with the findings from previous fossil pollen studies [12]. The most parsimonious hypothesis and scenario from our genetic data and ABC model testing would be to suggest that eastern white pine likely expanded northward along two routes of post-glacial recolonization from a single southern refugium (Fig. 6) that coincides well with the fossil pollen data [57]. The highest probability scenario from the ABC analysis and earlier fossil pollen evaluation [12] suggest that this refugium likely existed on the mid-Atlantic plain from coastal Virginia to the southern cost of South Carolina. The Ashville population from North Carolina is the only sampled location from an area that contained eastern white pine pollen from the LGM. This population showed the highest genetic diversity for both nuclear and chloroplast microsatellite markers (Tables 2 and 3), containing all five most abundant chloroplast microsatellite haplotypes (Fig. 4). This is typical for populations of glacial refugia. Thus, it is highly likely that the North Carolina sample location is a remnant of the eastern white pine LGM refugia. From the ABC analysis (Fig. 5) we can infer that much of the species’ range, to the east of the Great Lakes, is the product of a recolonization route that moved along the eastern seaboard (Fig. 6). The evolutionary history and scenario from our ABC analyses and the fossil pollen findings of Davis [12] and Jacobson [14] suggest that populations to the west of the Great Lakes, particularly in Minnesota and western Ontario, are likely descended from a second recolonization route, which was separated approximately 12,000 year ago (divergence time from ABC – Table 6; pollen existence time from Davis [12]), west of the Appalachian Mountains and south of the Great Lakes (Fig. 6).

In either scenario, the main northward recolonization route on the eastern seaboard is the source of the four most abundant cpSSR haplotypes observed in the eastern United States, Ontario, Quebec and the Maritime Provinces (Fig. 4). This route, supported by nuclear microsatellite Bayesian population structure and ABC models for both the nuclear and chloroplast markers, remained to the east of the Appalachian Mountains. A single branching point was identified in the vicinity of the southern Hudson River Valley (Fig. 6). The ABC analysis and the results from fossil pollen studies place this divergence at roughly 11,000 years ago (Figs. 5 and 6; Davies [12]). This northward and eastward divergence resulted in discontinuities between the haplotypes of the coastal (NH, ME, MA, NL, NS, NB) and central (NY, ON, QC) populations. The population isolation due to the geographical characteristics of this region, including the lowlands at the mouth of the Hudson River and the continuation of the northern Appalachian Mountains in New England States, was likely responsible for this divergence. During the glacial recession, climate in the mountains may have remained inhospitable to forest growth after northern lowlands became favourable, resulting in migration around these mountains, as inferred from the ABC scenarios. The high mountain altitudes and restricted valley habitats of northern New England may still minimize migration between populations on either side of these mountains. This hypothesis is supported by genetic similarities between populations in the eastern Quebec (including Cap Tourment and Temiscouta), which may represent a region with low levels of admixture through northern New Brunswick. The population in Newfoundland shares similar haplotypes with those in the Maritime Provinces and thus is likely descended from migrants from that region.

Under our primary hypothesis assuming one glacial refuge, the major population break observed between eastern and western eastern white pine populations was likely the result of two major features of eastern white pine’s geographical context. Initially, high altitude environments in the Appalachian Mountain may have separated the ancestral western migrants from their counterparts to the east of the mountains. Further in north and west, the Great Lakes may have reduced or prevented the dispersal of seeds between populations on the southern and western shores from the populations in Ontario. The cpSSR haplotype and ABC simulations results do not support the possibility that the western (Minnesota and westernmost Ontario) populations are the descendants of populations in Ontario. This inference is supported by the presence of cpSSR haplotypes in the western populations that are not found in any Ontario population (e.g., Haplotype AJ, Fig. 4). Additional support for a second recolonization route comes from the previous fossil pollen data studies [12]. Between roughly 10,000 and 8000 years ago, eastern white pine inhabited a range south of the Great Lakes (Indiana and Illinois) [12]. Though eastern white pine populations no longer exist in these areas, this is the most likely route of migration into Minnesota and western Ontario.

Although the post-glacial migration and evolution scenarios for eastern white pine were consistent between the chloroplast and nuclear data, the chloroplast data provided additional details, in particular regarding pollen dispersal. We observed shared cpSSR haplotypes between the isolated western populations (Boot Lake and Whale Lake, Minnesota and Crow Lake, Ontario) and the populations in central Ontario (Fig. 4). This was supported by an admixture event between the western and central lineages identified by the ABC scenarios (Fig. 5), possibly from pollen dispersal across a historical expanded range to the north of the Great Lakes [13] or pollen dispersal through fragmented forests in the northern peninsula of Michigan and Wisconsin. The sharing of chloroplast haplotypes (e.g. Haplotype AG, Fig. 4) between western populations and central Ontario populations may also indicate that the west to east prevailing winds have facilitated, or continue to facilitate, pollen dispersal between these regions. The opposite may be the case in between the central and coastal eastern populations. Between these regions, pollen dispersal by west to east prevailing winds may be limited by the northern Appalachian Mountains, leading to strong cpSSR haplotype differentiation (Fig. 4) as also supported by the rejection of the admixture simulation model between these regions by the ABC analysis (Table 5, Fig. 5).

The post-glacial phylogeographic patterns and evolutionary history of eastern white pine inferred in our study appear to be unusual as compared with those of other widely distributed tree species in North America, in that eastern white pine appears to have had a single glacial refugium and multiple post-glacial recolonization routes. In particular, populations of jack pine (*Pinus banksiana*), black spruce (*Picea mariana*), white spruce (*Picea glauca*), and red maple (*Acer rubrum*), three species found throughout the northern United States and Canada, have been reported to have descended from multiple glacial refugia [6, 8, 9, 58]. Jack pine has two distinct lineages, separated into populations in the Maritime Provinces of Canada, which originated from a northern refugium, and the rest of the species’ range, which originated from a southern refugium [9]. For white spruce, across a range similar to that of eastern white pine, two lineages descended from two southern refugia, Appalachian and Mississippian and one northern refugium in Alaska [6, 7]. Red maple populations originated from at least two populations on the eastern seaboard, one near the glacial margin and another more southern [56]. Two southern refugia have been identified for black spruce [8] and three for *Chamaecyparis thyoides* [10].

Overall our results validate our hypothesis that eastern white pine had a single southern LGM refugium but it took different post-glacial recolonization routes separated by Appalachian Mountains and Great Lakes, and the current distribution and population structure reflects the post-glacial migration history of the species.

### Human and natural disturbances and phylogeography signals

The recent human and natural disturbances can affect the genetic structure of the extant forest tree populations. The resulting genetic information can blur the genetic signals of post-glacial phylogeography and evolution. This was the case with the results of the STRUCTURE analysis in our study, which revealed 30 groups (26 from the BAPS analysis) among the 33 eastern white pine populations sampled. Only when we set the *K* values at 2 and 3 based on the results from the geographic distribution of the cpSSR haplotypes and Barrier analysis, the postglacial phylogeographic signals emerged from the STRUCTURE analysis. The ABC simulation analysis confirmed the phylogeogaphic patterns emerged. Thus, the cpSSR, ABC and Barrier analyses disentangled the blurring effects of human and natural disturbances from the genetic signals of postglacial evolution and expansion of eastern white pine populations. Hence our study highlights the necessity to disentangle the confounding effects of human and natural disturbances on the contemporary genetic structure from that due to post-glacial phylogeography and evolution.

### Evolutionary significant units and their genetic conservation implications

Our results indicate that eastern white pine populations have significant levels of genetic structure and differentiation across the species’ range. We have inferred three postglacial lineages in eastern white pine originating from a southern glacial refugium: eastern, central and western. Localized conservation and management strategies may be required in at least two and perhaps all three regions. The westernmost populations (Minnesota and western Ontario) represent a distinctive lineage and should be the focus of further study to determine if these populations contain adaptive traits for local conditions. As such, this lineage may represent a single Evolutionary Significant Unit (ESU) separated from the central and eastern populations. The divergence observed between the central and eastern coastal populations suggests that these lineages represent at least two additional ESUs. According to Ryder [59], who gave the concept of ESU, the ESUs are geographically and genetically diverged for both neutral genetic markers and adaptive traits. The three ESUs that we have identified in eastern white pine are geographically distinct and genetically diverged for presumably neutral genetic markers. We have not examined the variation in adaptive traits, which should be examined in future. Nevertheless, we have examined range-wide variation in SNPs in candidate genes putatively involved in controlling traits for local adaptation.

### Genetic resource conservation and climate change

As stated earlier in this paper, eastern white pine has been heavily harvested over the past 150 years [11], and consequently there are concerns about conservation of its genetic resources. Despite heavy exploitation over its range, and significant but low inter-population genetic differentiation, eastern white pine has maintained relatively high genetic diversity. This is likely due to presumably long distance gene flow and high inbreeding depression in eastern white pine [55], including selection against inbreds at a very early stage, as also reported for sympatric conifer white spruce [56]. We have examined genetic diversity in eastern white pine using only a handful of nuclear and chloroplast microsatellite markers, which by no means represent the whole nuclear and chloroplast genomic diversity. However, if the genetic diversity at the studied markers is a random sample of the species’ genetic diversity, genetic resources of eastern white pine are likely in good shape and could be conserved and sustainably managed in the extant natural populations, provided no further genetic degradation occurs. Therefore, the current and future harvesting practices should be genetically sound to maintain healthy genetic resources of this species, see [20, 22].

Eastern white pine has also gone through multiple episodes of post-glacial range expansion and retraction [18], encountering oscillation in climatic (such as temperature and moisture regimes) and topographical factors over time and space. Despite experiencing all of these events, eastern white pine has maintained genetic diversity, which provides raw material for species, populations and individuals to adapt and evolve, especially under changed climate, environment and disease conditions. This species is expected to migrate northwards under the predicted climate change conditions. Based on its past history of post-glacial migration and evolution, eastern white pine may be able to cope with the anticipated climate change conditions. Its marginal populations, especially at the northern margins of its range, will likely play a major role in its northward range expansion. We have examined genetic diversity at the microsatellite markers, which are considered to be selectively neutral. We suggest that genetic diversity of range-wide as well as marginal populations should be studied at a large number of markers from genes under selection (such as SNPs).

## Conclusions

Eastern white pine has relatively high magnitude of genetic diversity, and significant differentiation and genetic structure across its natural range. Its contemporary population genetic structure shows the signatures of post-glacial migration and evolution as well as effects of natural and human disturbances. The current distribution of eastern white pine is the result of at least two post-glacial recolonization routes from a southern single glacial refugium. The two regions of greatest genetic differentiation corresponding to post-glacial recolonization routes are: (1) west of the Great Lakes and (2) along the eastern seaboard. However, it cannot be determined from the markers used in our study whether any of the geographic patterns in population genetic structure is adaptive. We have identified three ESUs (western, central and eastern) in eastern white pine which should be taken into account in conserving and managing the species’ genetic resources. If future work also finds evidence for adaptive differentiation among the identified western, central and eastern coastal genetic lineages, eastern white pine conservation and genetic resource management plans should be made specific to each of these three regions, especially under the climate change conditions. In order to better delineate genetic lineages resulting from post-glacial migration, it is necessary to disentangle the confounding genetic signatures of human and natural disturbances on the contemporary genetic structure from that due to post-glacial phylogeography and evolution.

### Availability of supporting data

The raw nuclear and chloroplast microsatellite data are provided in Additional file 6: Table S3 and Additional file 7: Table S4. The supporting results and data are provided in the Additional files 1, 2, 3, 4 and 5.

## Additional files

Additional file 1: Table S1. Definition and prior distribution of parameters used in the ABC tests of various eastern white pine phylogeographic divergence scenarios for four groups. (DOCX 15 kb) Additional file 2: Figure S1. Competing phylogeographic scenarios of eastern white pine population group divergence and admixture. Sc1, Sc2, Sc3: Scenarios without admixture. Sc4 and Sc5: Scenarios with admixture. ST: southern group. EST: eastern group. CNT: central group. WS: western group. t_0_-t_3_: divergence times. AD: admixture events. Information on groups is provided in Additional file 1: Table S1. (DOCX 799 kb) Additional file 3: Table S2. Allele composition of all chloroplast microsatellite haplotypes (A-BH) derived from three chloroplast microsatellite markers. (DOCX 16 kb) Additional file 4: Figure S2. Summary scatterplot of Delta *K* values for eastern white pine populations testing (*K=*) 1 – 33 clusters calculated from the STRUCTURE results using the Evanno et al. [42] method. (DOCX 24 kb) Additional file 5: Figure S3. Geographic patterns of genetic variation among populations from BAPS analysis. Locations contained within a box represent sampled locations that were clustered into a single population by Bayesian algorithms (BAPS, Corander et al., 2004 [43]). Genetic barriers, identified by Monmonier’s algorithms, are represented by solid lines labeled A (supported by 12 nuclear microsatellite markers, and 3 chloroplast microsatellite markers) and B (supported by 10 nuclear microsatellite markers and 3 chloroplast microsatellite markers). See Fig. 1 and Table 1 for the population names and details. (DOCX 208 kb) Additional file 6: Table S3. Nuclear microsatellite genotype data. (PDF 996 kb) Additional file 7: Table S4. Chloroplast microsatellite genotype data. (PDF 138 kb)
