# Supplementary material for: Post-glacial phylogeography and evolution of a wide-ranging highly-exploited keystone forest tree, eastern white pine (Pinus strobus) in North America: single refugium, multiple routes
Source: BMC Evol Biol. 2016 Mar 2;16:56. doi: 10.1186/s12862-016-0624-1 (PMC4774161; doi:10.1186/s12862-016-0624-1)
Supplement: Additional file 1: Table S1. — Definition and prior distribution of parameters used in the ABC tests of various eastern white pine phylogeographic divergence scenarios for four groups. (DOCX 15 kb) [file 12862_2016_624_MOESM1_ESM.docx]

**Table S1**: Definition and prior distribution of parameters used in the Approximate Bayesian Computation (ABC) tests of eastern white pine phylogeographic divergence scenarios for four groups

| **Parameter name** | **Parameter abbreviation** | | **Distribution** |  | |
| --- | --- | --- | --- | --- | --- |
|  |  | | **Type** | **Interval** | |
| **Effective sizes** |  | |  |  | |
| Effective sizes of the ancestral population | N_A_ | | Uniform | [10-1000000] | |
| Effective size - Western group  ONCL, MNBL, MNWL | N_WS_ | | Uniform | [10-500000] | |
| Effective size - Central group | N_CNT_ | | Uniform | [10-500000] | |
| QCTM, QCCT, QCSR, QCSS, QCLP, ONLM, ONFR, ONHF, ONGR, ONMW, ONRC, ONWL, ONTO, NYCM, PAOL |  |  | | |  |
| Effective size - Eastern group | N_EST_ | | Uniform | [10-500000] | |
| NLGL, NSMB, NSRL, NSDL, NSUM, NBPH, NBCI, NBCR, NBOP, MEEB, MEBP, NHDF, MASB |  |  | | |  |
| Effective size - Southern group | N_ST_ | | Uniform | [10-500000] | |
| NCAV, VABS |  |  | | |  |
|  |  | |  |  | |
| **Time Parameters** |  | |  |  | |
| Expansion event | t_0_, t_1_, t_2_, t_3_ | | Uniform | [10-50000] | |
| Admixture event | t_adm1,_ t_adm2_ | | Uniform | [10-100000] | |
| Time of the ancestral divergence | Varies (t_0_-t_3_) | | Uniform | [100-1000000] | |
| Mean mutation rate (site/generation) | Mµ | | Uniform | [10^-5^ -10^-3^] | |
| Rate of admixture | ra | | Uniform | [0.01–0.99] | |
