# Supplementary material for: Post-glacial phylogeography and evolution of a wide-ranging highly-exploited keystone forest tree, eastern white pine (Pinus strobus) in North America: single refugium, multiple routes
Source: BMC Evol Biol. 2016 Mar 2;16:56. doi: 10.1186/s12862-016-0624-1 (PMC4774161; doi:10.1186/s12862-016-0624-1)
Supplement: Additional file 2: Figure S1. — Competing phylogeographic scenarios of eastern white pine population group divergence and admixture. Sc1, Sc2, Sc3: Scenarios without admixture. Sc4 and Sc5: Scenarios with admixture. ST: southern group. EST: eastern group. CNT: central group. WS: western group. t0-t3: divergence times. AD: admixture events. Information on groups is provided in Additional file 1: Table S1. (DOCX 799 kb) [file 12862_2016_624_MOESM2_ESM.docx]

**
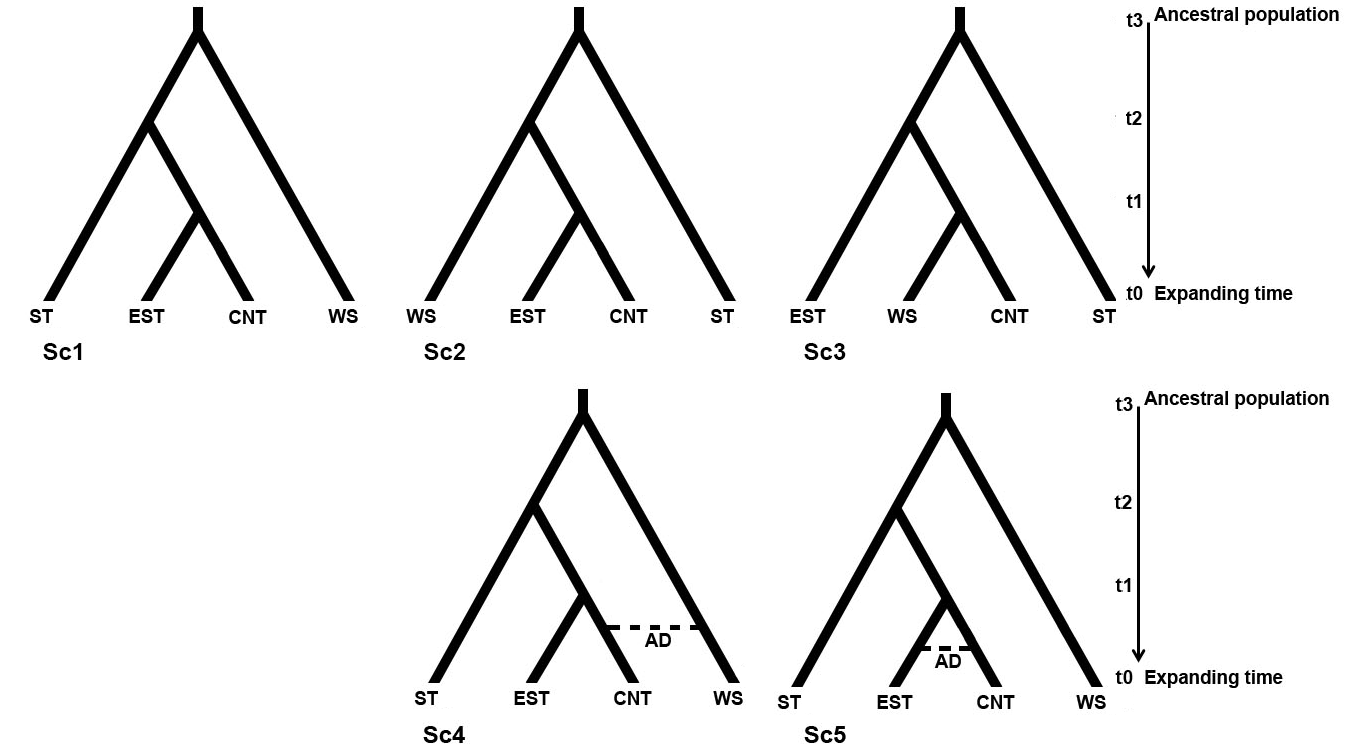
**

**Figure S1**: Competing phylogeographic scenarios of eastern white pine regional group divergence and admixture. Sc1, Sc2, Sc3: Scenarios without admixture. Sc4 and Sc5: Scenarios with admixture. ST: southern group. EST: eastern group. CNT: central group. WS: western group. t_0_-t_3_: divergence times. AD: admixture events. Information on groups is provided in Additional File 1: Table S1.
