# Supplementary material for: Post-glacial phylogeography and evolution of a wide-ranging highly-exploited keystone forest tree, eastern white pine (Pinus strobus) in North America: single refugium, multiple routes
Source: BMC Evol Biol. 2016 Mar 2;16:56. doi: 10.1186/s12862-016-0624-1 (PMC4774161; doi:10.1186/s12862-016-0624-1)
Supplement: Additional file 3: Table S2. — Allele composition of all chloroplast microsatellite haplotypes (A-BH) derived from three chloroplast microsatellite markers. (DOCX 16 kb) [file 12862_2016_624_MOESM3_ESM.docx]

**Table S2 A**llele composition of all chloroplast microsatellite haplotypes (A-BH)

derived from three chloroplast microsatellite markers.

| Haplotype name | | Allelic composition  pt26081 pt63718 pt71936 | | |
| --- | --- | --- | --- | --- |
| Haplotype | A | 126 | 113 | 161 |
| Haplotype | B | 126 | 115 | 159 |
| Haplotype | C | 126 | 114 | 161 |
| Haplotype | D | 126 | 113 | 163 |
| Haplotype | E | 126 | 114 | 163 |
| Haplotype | F | 126 | 114 | 165 |
| Haplotype | G | 126 | 114 | 167 |
| Haplotype | H | 126 | 115 | 161 |
| Haplotype | I | 126 | 115 | 163 |
| Haplotype | J | 126 | 115 | 165 |
| Haplotype | K | 126 | 115 | 169 |
| Haplotype | L | 126 | 116 | 163 |
| Haplotype | M | 128 | 115 | 161 |
| Haplotype | N | 128 | 115 | 163 |
| Haplotype | O | 132 | 113 | 163 |
| Haplotype | P | 134 | 114 | 163 |
| Haplotype | Q | 134 | 115 | 163 |
| Haplotype | R | 136 | 113 | 161 |
| Haplotype | S | 136 | 113 | 163 |
| Haplotype | T | 136 | 113 | 165 |
| Haplotype | U | 136 | 114 | 161 |
| Haplotype | V | 136 | 114 | 163 |
| Haplotype | W | 136 | 114 | 165 |
| Haplotype | X | 136 | 114 | 167 |
| Haplotype | Y | 136 | 114 | 169 |
| Haplotype | Z | 136 | 115 | 159 |
| Haplotype | AA | 136 | 115 | 161 |
| Haplotype | AB | 136 | 115 | 163 |
| Haplotype | AC | 136 | 115 | 165 |
| Haplotype | AD | 136 | 115 | 167 |
| Haplotype | AE | 136 | 115 | 169 |
| Haplotype | AF | 136 | 116 | 159 |
| Haplotype | AG | 136 | 116 | 161 |
| Haplotype | AH | 136 | 116 | 163 |
| Haplotype | AI | 136 | 116 | 169 |
| Haplotype | AJ | 136 | 117 | 161 |
| Haplotype | AK | 138 | 113 | 163 |
| Haplotype | AL | 138 | 114 | 163 |
| Haplotype | AM | 138 | 114 | 169 |
| Haplotype | AN | 138 | 115 | 159 |
| Haplotype | AO | 138 | 115 | 161 |
| Haplotype | AP | 138 | 115 | 163 |
| Haplotype | AQ | 138 | 115 | 165 |
| Haplotype | AR | 138 | 115 | 167 |
| Haplotype | AS | 138 | 115 | 169 |
| Haplotype | AT | 138 | 115 | 177 |
| Haplotype | AU | 138 | 116 | 159 |
| Haplotype | AV | 138 | 116 | 161 |
| Haplotype | AW | 138 | 116 | 163 |
| Haplotype | AX | 138 | 116 | 169 |
| Haplotype | AY | 142 | 115 | 159 |
| Haplotype | AZ | 140 | 115 | 151 |
| Haplotype | BA | 140 | 115 | 159 |
| Haplotype | BB | 140 | 115 | 161 |
| Haplotype | BC | 140 | 115 | 163 |
| Haplotype | BD | 140 | 115 | 167 |
| Haplotype | BE | 140 | 116 | 159 |
| Haplotype | BF | 140 | 116 | 161 |
| Haplotype | BG | 140 | 116 | 163 |
| Haplotype | BH | 142 | 115 | 163 |
