# Supplementary material for: Post-glacial phylogeography and evolution of a wide-ranging highly-exploited keystone forest tree, eastern white pine (Pinus strobus) in North America: single refugium, multiple routes
Source: BMC Evol Biol. 2016 Mar 2;16:56. doi: 10.1186/s12862-016-0624-1 (PMC4774161; doi:10.1186/s12862-016-0624-1)
Supplement: Additional file 4: Figure S2. — Summary scatterplot of Delta K values for eastern white pine populations testing (K=) 1 – 33 clusters calculated from the STRUCTURE results using the Evanno et al. [42] method. (DOCX 24 kb) [file 12862_2016_624_MOESM4_ESM.docx]

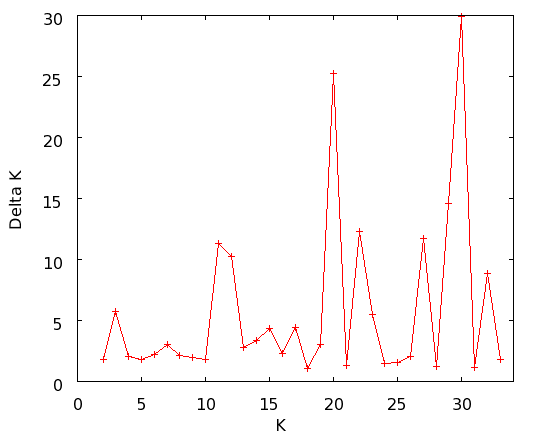


**Figure S2** Summary scatterplot of Delta *K* values for eastern white pine populations testing (*K=*) 1 – 33 cluster calculated from the STRUCTURE results using the Evanno *et al.* (2005) method.
