# Supplementary material for: Post-glacial phylogeography and evolution of a wide-ranging highly-exploited keystone forest tree, eastern white pine (Pinus strobus) in North America: single refugium, multiple routes
Source: BMC Evol Biol. 2016 Mar 2;16:56. doi: 10.1186/s12862-016-0624-1 (PMC4774161; doi:10.1186/s12862-016-0624-1)
Supplement: Additional file 5: Figure S3. — Geographic patterns of genetic variation among populations from BAPS analysis. Locations contained within a box represent sampled locations that were clustered into a single population by Bayesian algorithms (BAPS, Corander et al., 2004 [43]). Genetic barriers, identified by Monmonier’s algorithms, are represented by solid lines labeled A (supported by 12 nuclear microsatellite markers, and 3 chloroplast microsatellite markers) and B (supported by 10 nuclear microsatellite markers and 3 chloroplast microsatellite markers). See Fig. 1 and Table 1 for the population names and details. (DOCX 208 kb) [file 12862_2016_624_MOESM5_ESM.docx]

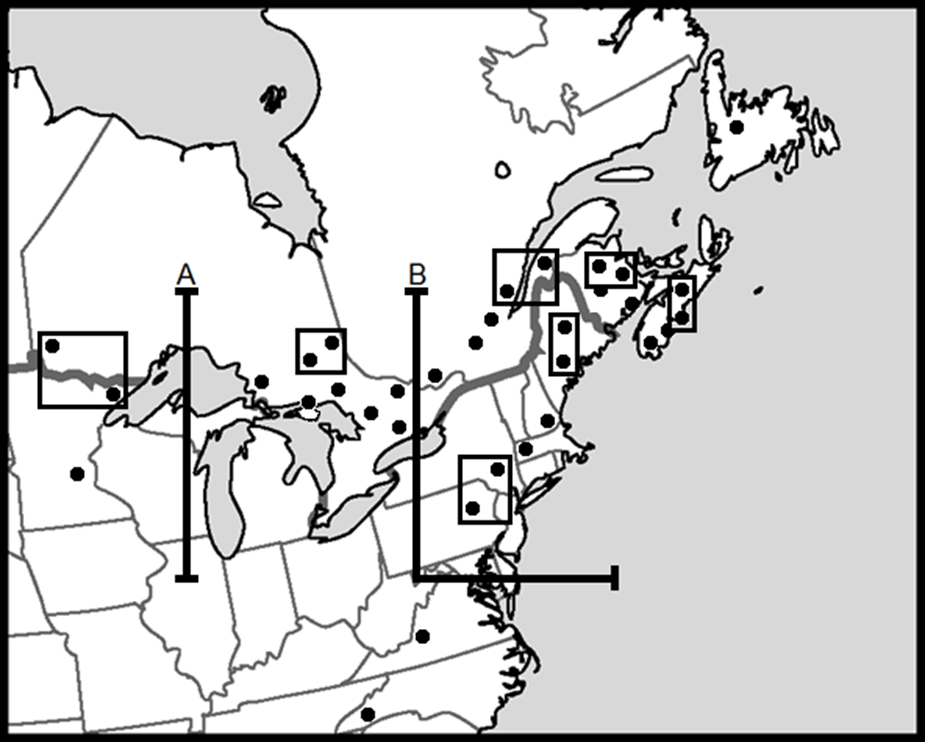


**Figure S3** Geographic patterns of genetic variation among populations from BAPS analysis. Locations contained within a box represent sampled locations that were clustered into a single population by Bayesian algorithms (BAPS, Corander et al., 2004 [43]). Genetic barriers, identified by Monmonier’s algorithms, are represented by solid lines labeled A (supported by 12 nuclear microsatellite markers, and 3 chloroplast microsatellite markers) and B (supported by 10 nuclear markers and 3 chloroplast markers). See Figure 1 and Table 1 for the population names and details.
