# Supplementary material for: Post-glacial phylogeography and evolution of a wide-ranging highly-exploited keystone forest tree, eastern white pine (Pinus strobus) in North America: single refugium, multiple routes
Source: BMC Evol Biol. 2016 Mar 2;16:56. doi: 10.1186/s12862-016-0624-1 (PMC4774161; doi:10.1186/s12862-016-0624-1)
Supplement: Additional file 6: Table S3. — Nuclear microsatellite genotype data. (PDF 996 kb) [file 12862_2016_624_MOESM6_ESM.pdf]

Additional File 3

Post-glacial phylogeography and evolution of a wide-ranging highly-exploited keystone forest tree, eastern white pine (Pinus strobus) in North America: Single refugium, multiple routes

John W. R. Zinck and Om P. Rajora

Table S3. Nuclear microsatellite genotype data

	RSP1		RSP2		RSP12		RSP20		RSP25		RSP34b		RSP39		RSP50		RSP60		RSP127		RSP118		RSP119	
NFGL01	230	232	183	183	204	204	158	164	128	132	176	184	193	195	190	194	255	259	212	214	139	145	163	165
NFGL02	228	228	175	189	204	204	158	170	126	128	174	176	193	193	202	204	255	261	212	214	139	145	163	165
NFGL03	228	228	181	183	194	206	158	158	136	136	192	192	189	191	190	192	257	257	214	214	139	147	165	165
NFGL04	230	230	183	185	206	208	152	152	128	130	168	170	193	193	186	194	255	271	214	216	139	147	165	167
NFGL05	230	234	183	183	210	210	158	160	130	132	170	170	191	191	164	188	257	271	214	214	139	145	165	165
NFGL06	238	238	183	185	210	224	164	164	130	130	168	168	189	191	188	190	255	273	212	214	137	147	163	165
NFGL07	230	234	171	187	208	208	152	158	128	132	166	166	189	191	164	164	255	271	214	214	139	145	165	165
NFGL08	230	234	183	191	190	210	148	158	128	130	166	168	191	203	184	192	251	263	214	216	139	145	165	167
NFGL09	232	232	183	185	188	204	158	158	130	130	192	192	189	191	190	190	255	273	214	216	139	145	165	167
NFGL10	232	234	183	183	206	206	158	172	128	132	156	166	191	191	164	194	255	271	192	216	139	145	167	167
NFGL11	226	228	175	187	196	212	160	160	126	132	174	178	189	191	192	194	251	269	212	216	139	145	163	167
NFGL12	228	228	183	185	208	208	158	158	126	128	170	172	189	193	190	190	253	253	214	214	139	145	165	165
NFGL13	230	232	183	195	190	220	152	152	132	132	168	168	189	189	186	190	255	271	214	214	139	145	165	165
NFGL14	228	228	187	187	208	208	172	172	128	130	174	176	195	195	198	200	255	261	214	214	139	145	165	165
NFGL15	228	228	183	185	204	204	162	176	136	136	186	188	191	193	190	190	267	271	214	218	139	145	165	169
NFGL16	228	228	185	191	192	192	176	176	134	134	186	188	191	191	196	196	251	251	214	216	139	145	165	167
NFGL17	228	228	185	185	206	208	168	168	128	132	174	176	191	191	202	202	255	255	216	218	139	145	167	169
NFGL18	228	228	171	183	182	184	158	162	130	130	188	188	179	193	188	198	253	257	212	214	139	145	163	165
NFGL19	230	234	183	195	204	208	152	168	130	130	176	184	193	195	190	190	255	259	212	216	139	145	163	167
NFGL20	228	234	183	197	212	214	160	176	130	132	156	168	191	191	188	192	257	271	214	216	139	145	165	167
NFGL21	230	238	185	185	210	216	154	162	134	136	144	162	181	181	164	188	255	271	214	216	139	145	165	167
NFGL22	228	228	183	187	190	206	160	176	132	136	190	190	193	193	192	200	253	271	212	216	139	145	163	167
NFGL23	230	232	183	191	206	210	158	160	130	132	168	168	189	189	188	190	257	269	170	214	139	145	165	165
NFGL24	232	234	183	183	208	208	160	174	128	130	168	168	191	191	184	190	257	271	192	216	139	145	167	167
NFGL25	228	228	183	183	194	208	158	158	130	134	186	188	177	185	188	190	255	255	214	214	139	145	165	165
NFGL26	230	234	187	197	210	210	154	158	132	134	166	166	189	199	190	190	255	271	214	216	137	145	165	167
NFGL27	228	228	183	185	194	208	160	176	134	134	188	188	189	193	190	200	269	271	212	216	139	145	163	167
NFGL28	228	228	181	185	202	202	174	174	128	130	170	176	191	193	190	198	255	257	214	214	139	145	165	165
NFGL29	228	228	185	185	206	206	172	172	122	122	174	176	195	195	198	200	255	261	198	214	139	145	149	165
NFGL30	232	238	181	181	208	210	160	176	124	128	166	184	191	193	190	192	255	271	214	218	139	145	165	169
NFGL31	222	222	169	185	206	206	158	174	122	124	170	176	185	193	188	200	253	253	214	214	139	145	165	165
NFGL32	228	234	185	197	188	188	160	174	130	130	156	166	187	189	186	194	255	271	214	214	137	145	165	165
NFGL33	228	228	183	183	202	206	174	174	132	136	190	190	191	199	188	194	257	269	212	218	141	145	163	169
NFGL34	228	228	187	189	196	210	160	176	118	120	172	174	189	193	190	200	255	271	212	216	137	145	163	167
NFGL35	228	228	179	185	200	210	148	158	132	132	166	166	189	189	190	190	255	271	214	214	139	145	165	165
NFGL36	230	232	183	195	186	188	146	158	130	130	166	186	185	193	190	190	257	271	212	214	139	145	163	165
NFGL37	222	222	189	191	208	210	176	176	132	134	174	176	187	191	188	200	253	257	214	214	139	145	165	165
NFGL38	228	228	187	187	206	206	162	172	136	138	174	176	193	193	198	200	255	261	198	214	141	147	149	165
NFGL39	228	228	181	183	204	208	158	164	126	128	176	178	191	193	188	190	253	253	214	214	141	147	165	165
NFGL40	230	234	183	197	210	210	158	174	130	130	166	168	189	191	190	198	255	271	214	214	137	145	165	165
NFGL41	228	234	183	185	208	208	158	172	124	126	176	178	195	195	194	194	253	253	216	216	139	145	167	167
NFGL42	228	228	183	185	198	202	158	160	140	140	188	188	189	193	188	200	253	253	214	216	139	147	165	167
NFGL43	228	228	187	189	194	194	176	176	130	132	170	174	191	191	196	196	251	251	214	216	139	145	165	167
NFGL44	228	228	183	183	204	204	158	158	132	132	192	192	189	191	188	188	255	257	214	216	139	145	165	167
NFGL45	222	228	183	183	194	194	150	150	134	134	188	188	187	191	198	198	253	277	212	218	139	145	163	169
NFGL46	230	232	183	183	210	228	158	168	130	132	168	168	193	193	186	190	255	271	214	214	139	145	165	165
NFGL47	226	228	181	185	194	210	160	160	134	134	190	192	189	191	192	194	257	269	212	216	141	145	163	167
NFGL48	230	234	181	195	206	208	160	176	130	134	184	184	191	193	164	190	255	271	216	216	139	145	167	167
NFGL49	228	234	181	183	208	210	144	158	130	132	174	176	191	193	190	192	253	255	214	216	141	145	165	167
NFGL50	230	234	183	183	204	210	164	164	130	130	144	166	177	191	164	188	255	271	212	216	139	145	163	167
NBPMH01	230	232	183	183	204	204	158	164	128	132	176	184	193	195	190	194	255	259	212	214	139	147	163	165
NBPMH02	230	234	183	195	204	208	152	168	130	130	176	184	193	195	190	190	255	259	212	216	139	145	163	167
NBPMH03	230	232	183	195	186	188	146	158	130	130	166	186	185	193	190	190	257	271	212	214	139	145	163	165
NBPMH04	228	234	183	197	212	214	160	176	130	132	156	168	191	191	188	192	257	271	214	216	139	145	165	167
NBPMH05	230	230	185	197	208	208	158	176	128	132	164	168	191	193	192	196	255	275	170	216	139	145	167	167
NBPMH06	230	230	183	183	204	214	160	174	132	132	168	168	191	191	192	196	255	273	214	216	139	145	165	167
NBPMH07	230	230	183	185	208	210	150	150	130	130	166	170	179	191	190	192	255	271	198	214	139	145	149	165
NBPMH08	230	232	183	185	208	210	152	152	132	132	146	164	189	191	190	192	241	253	214	214	139	147	165	165
NBPMH09	230	230	183	183	174	174	158	174	132	160	166	166	189	201	188	192	255	269	170	214	139	145	165	165
NBPMH10	230	234	187	187	192	210	152	162	130	134	156	166	189	191	190	190	255	271	212	214	137	145	163	165
NBPMH11	230	238	185	185	210	216	154	162	134	136	144	162	181	181	164	188	255	271	214	216	139	145	165	167
NBPMH12	230	234	187	197	210	210	154	158	132	134	166	166	189	199	190	190	255	271	214	216	139	145	165	167
NBPMH13	228	228	179	185	200	210	148	158	132	132	166	166	189	189	190	190	255	271	214	214	139	145	165	165
NBPMH14	230	234	171	187	208	208	152	158	128	132	166	166	189	191	164	164	255	271	214	214	137	145	165	165
NBPMH15	234	240	183	185	188	210	164	164	128	132	166	182	175	189	190	190	239	263	212	216	139	145	163	167
NBPMH16	232	238	185	199	172	210	148	156	134	134	166	166	189	191	188	206	255	271	214	214	139	145	165	165
NBPMH17	232	234	185	185	204	216	146	158	130	130	166	166	191	191	194	194	255	273	170	214	139	147	165	165
NBPMH18	232	232	183	183	204	216	166	174	126	132	144	162	175	189	196	198	255	271	212	216	139	145	163	167
NBPMH19	232	234	183	183	190	224	154	174	130	132	156	166	175	193	188	200	255	271	212	216	137	145	163	167
NBPMH20	232	232	183	197	208	210	158	172	130	130	166	168	189	191	164	190	255	271	192	214	139	145	165	165
NBPMH21	232	234	183	183	206	206	158	172	128	132	156	166	191	191	164	194	255	271	192	216	139	145	167	167
NBPMH22	230	234	183	183	204	210	164	164	130	130	144	166	177	191	164	188	255	271	212	216	139	145	163	167
NBPMH23	228	234	185	197	188	188	160	174	130	130	156	166	187	189	186	194	255	271	214	214	139	145	165	165
NBPMH24	230	234	183	197	210	210	158	174	130	130	166	168	189	191	190	198	255	271	214	214	139	145	165	165
NBPMH25	232	238	181	181	208	210	160	176	124	128	166	184	191	193	190	192	255	271	214	218	139	147	165	169
NBPMH26	230	234	181	195	206	208	160	176	130	134	184	184	191	193	164	190	255	271	216	216	139	145	167	167
NBPMH27	226	234	183	193	188	204	160	174	130	134	184	184	189	191	164	192	257	271	216	216	139	145	167	167
NBPMH28	216	232	183	195	188	208	160	174	128	132	174	184	191	191	190	200	257	271	214	214	139	145	165	165
NBPMH29	232	232	183	195	210	210	152	152	128	130	174	184	191	193	192	198	257	271	192	214	139	145	165	165
NBPMH30	232	238	193	195	188	208	158	158	130	130	174	184	191	193	190	198	255	259	214	216	139	145	165	167
NBPMH31	230	234	183	191	210	210	158	176	130	132	174	184	191	193	188	194	257	269	214	216	139	145	165	167
NBPMH32	230	232	169	191	182	210	160	176	130	132	174	184	191	193	190	196	257	269	192	216	139	145	167	167
NBPMH33	230	232	183	191	206	210	158	160	130	132	168	168	189	189	188	190	257	269	170	214	139	145	165	165
NBPMH34	238	238	183	185	210	224	164	164	130	130	168	168	189	191	188	190	255	273	212	214	139	145	163	165
NBPMH35	230	232	183	195	190	220	152	152	132	132	168	168	189	189	186	190	255	271	214	214	139	145	165	165
NBPMH36	230	232	183	183	210	228	158	168	130	132	168	168	193	193	186	190	255	271	214	214	137	145	165	165
NBPMH37	230	230	183	185	206	208	152	152	128	130	168	170	193	193	186	194	255	271	214	216	139	145	165	167
NBPMH38	230	234	183	183	208	208	160	176	126	132	168	168	191	191	186	190	255	273	216	216	139	145	167	167
NBPMH39	230	232	183	191	190	208	164	174	128	130	166	166	193	201	188	196	255	273	212	216	139	145	163	167
NBPMH40	230	232	185	195	202	228	148	158	126	126	166	168	191	193	190	190	255	273	214	214	139	145	165	165
NBPMH41	218	234	183	183	182	190	160	176	126	126	166	166	191	193	194	204	257	271	214	218	139	147	165	169
NBPMH42	232	232	193	197	208	210	160	164	128	130	168	168	191	193	164	194	257	273	192	214	139	145	165	165
NBPMH43	232	232	183	185	206	210	160	164	128	130	168	168	193	193	164	164	257	273	198	214	139	145	149	165
NBPMH44	230	234	183	191	190	210	148	158	128	130	166	168	191	203	184	192	251	263	214	216	137	145	165	167
NBPMH45	232	234	183	183	208	208	160	174	128	130	168	168	191	191	184	190	257	271	192	216	139	145	167	167
NBPMH46	230	234	183	183	210	210	158	160	130	132	170	170	191	191	164	188	257	271	214	214	139	145	165	165
NBPMH47	232	232	185	191	190	210	162	166	132	134	168	168	193	193	186	190	257	271	214	214	139	145	165	165
NBPMH48	230	234	183	185	206	206	168	168	126	130	168	168	191	193	160	190	257	271	214	216	139	145	165	167
NBPMH49	230	234	185	195	190	210	158	176	132	132	166	168	191	193	196	198	255	273	212	216	139	145	163	167
NBPMH50	218	232	183	185	210	210	140	156	130	134	168	168	201	201	196	206	255	273	212	216	139	145	163	167
NBCI01	204	228	183	183	210	210	156	170	130	130	164	168	191	193	190	190	255	255	212	212	139	147	167	167
NBCI02	230	230	183	183	190	204	154	172	122	124	166	170	191	193	188	190	255	259	212	214	139	145	167	169
NBCI03	228	230	177	191	190	210	148	156	128	130	166	168	179	193	188	198	257	257	212	214	139	145	167	169
NBCI04	230	230	179	181	190	210	158	172	138	138	164	168	193	203	188	190	257	257	212	212	139	145	167	167
NBCI05	228	232	181	181	206	206	156	172	146	146	164	166	177	193	194	196	257	257	212	212	139	145	167	167
NBCI06	230	230	183	185	208	208	156	172	0	0	164	166	193	193	190	200	255	255	212	212	137	145	167	167
NBCI07	232	246	181	183	214	214	168	168	128	130	156	166	179	191	188	188	257	261	212	212	139	145	167	167
NBCI08	230	230	167	181	208	216	156	156	126	134	146	160	185	185	200	200	257	261	212	214	139	145	167	169
NBCI09	228	230	181	191	190	206	156	156	130	134	164	166	177	191	186	190	255	273	212	214	139	147	167	169
NBCI10	232	244	173	181	202	208	168	170	132	132	164	166	177	191	188	188	257	259	212	212	141	145	167	167
NBCI11	230	246	169	185	206	210	152	160	126	128	164	168	185	195	196	196	259	259	212	214	139	145	167	169
NBCI12	230	246	183	183	192	210	156	156	128	132	164	168	191	193	198	198	257	261	212	212	139	145	167	167
NBCI13	230	234	169	183	204	204	156	160	132	132	156	166	191	193	200	200	259	261	214	214	139	145	169	169
NBCI14	232	246	183	183	186	208	168	172	130	130	164	164	189	193	192	192	259	259	212	212	139	147	167	167

NBCI15	230	230	169	183	206	208	158	172	128	130	164	168	183	193	188	194	259	259	212	212	139	147	167	167
NBCI16	228	228	183	183	206	206	156	172	130	130	156	166	183	193	230	252	255	259	212	214	139	147	167	169
NBCI17	228	228	183	183	214	214	156	158	130	132	156	166	191	191	230	252	255	261	212	214	139	147	167	169
NBCI18	228	228	183	183	208	210	158	170	126	130	164	168	191	201	190	194	259	273	212	212	139	145	167	167
NBCI19	228	228	183	183	190	218	156	156	134	134	164	166	179	193	180	188	255	257	216	216	139	145	171	171
NBCI20	228	228	181	191	206	206	156	156	128	132	156	166	191	191	188	190	255	255	212	214	139	145	167	169
NBCI21	228	228	169	183	208	208	156	156	128	128	144	166	193	193	190	190	255	255	212	214	137	145	167	169
NBCI22	194	194	183	183	206	208	156	156	132	132	144	156	191	193	190	190	259	259	210	214	139	145	165	169
NBCI23	228	232	183	183	206	206	158	174	128	128	164	166	193	193	196	204	259	265	212	212	139	145	167	167
NBCI24	228	228	181	181	190	190	160	170	130	132	156	164	193	193	188	188	259	259	212	212	139	145	167	167
NBCI25	228	228	181	185	180	190	158	172	130	130	156	164	193	201	200	200	255	255	212	212	139	145	167	167
NBCI26	228	228	183	185	172	202	156	162	130	130	166	170	191	201	200	200	257	259	216	216	139	145	171	171
NBCI27	230	244	177	183	190	210	152	160	128	128	168	170	181	193	190	194	257	259	212	212	139	145	167	167
NBCI28	230	232	183	191	186	210	160	172	128	130	168	168	191	193	188	190	255	255	212	212	139	145	167	167
NBCI29	230	230	183	183	172	172	158	160	128	130	166	168	191	193	190	190	255	255	216	216	137	145	171	171
NBCI30	230	230	183	185	192	192	172	172	130	130	166	168	189	193	182	188	255	255	212	212	139	145	167	167
NBCI31	230	230	183	183	208	210	158	170	130	130	166	168	189	191	192	192	255	255	210	212	139	145	165	167
NBCI32	228	228	183	209	192	208	154	158	132	132	166	168	189	191	188	188	255	255	212	214	139	145	167	169
NBCI33	228	228	183	183	186	208	156	156	132	132	166	166	201	201	190	192	255	255	212	214	139	147	167	169
NBCI34	224	232	185	185	184	204	158	158	130	132	166	166	189	189	194	200	253	257	212	214	139	145	167	169
NBCI35	226	236	185	191	212	216	150	158	122	128	166	168	185	189	194	200	255	259	0	0	139	145	0	0
NBCI36	226	236	183	183	192	204	172	174	130	132	166	168	191	201	188	204	257	261	212	214	135	145	167	169
NBCI37	226	232	183	183	192	204	156	156	132	132	156	166	191	191	194	206	255	259	212	212	139	145	167	167
NBCI38	228	228	183	191	180	192	172	184	132	136	166	166	189	189	178	188	255	255	204	204	139	145	159	159
NBCI39	228	232	183	185	208	212	158	172	130	130	166	166	189	191	190	190	255	255	212	212	139	145	167	167
NBCI40	232	232	185	185	208	210	156	168	128	136	166	166	189	189	190	200	255	255	212	212	139	145	167	167
NBCI41	232	232	183	183	210	216	170	172	130	132	156	166	189	189	194	200	255	255	212	214	139	145	167	169
NBCI42	232	232	183	185	206	206	150	164	130	130	164	166	187	189	188	200	261	261	212	216	139	145	167	171
NBCI43	230	230	169	185	196	208	150	158	130	132	164	166	177	191	192	202	261	261	212	212	139	145	167	167
NBCI44	238	238	181	183	192	214	158	158	132	132	156	166	187	189	196	198	261	261	212	212	139	145	167	167
NBCI45	232	232	183	185	192	212	158	160	128	128	156	166	191	197	194	200	257	261	214	214	139	145	169	169
NBCI46	230	232	183	183	192	212	158	160	128	130	156	184	189	191	200	200	259	277	212	216	139	145	167	171
NBCI47	232	232	183	183	208	212	158	166	130	132	156	166	191	191	188	200	257	257	212	216	135	145	167	171
NBCI48	234	234	183	183	188	212	158	158	130	130	166	166	189	191	194	198	257	257	212	212	139	145	167	167
NBCI49	230	230	181	181	212	212	158	158	132	132	166	166	189	191	190	190	257	257	212	212	139	145	167	167
NBCI50	224	230	183	185	210	210	156	156	134	138	156	166	175	189	188	190	255	259	212	212	139	145	167	167
NBCR01	230	234	185	187	212	214	144	160	124	128	168	168	191	193	192	196	251	251	214	214	139	147	165	165
NBCR02	230	230	185	187	198	212	148	162	126	128	168	168	193	193	190	190	251	251	216	216	139	145	167	167
NBCR03	230	232	175	187	206	210	148	162	124	130	168	170	189	191	188	202	251	255	170	214	139	145	165	165
NBCR04	232	232	185	187	210	212	150	162	124	130	146	170	193	201	160	200	251	251	198	214	139	145	149	165
NBCR05	232	232	175	189	208	210	148	160	122	126	162	166	191	191	188	198	251	251	198	214	139	147	149	165
NBCR06	232	232	175	187	210	210	158	168	126	130	142	166	191	191	192	194	251	251	212	216	137	147	163	167
NBCR07	232	234	175	185	210	214	158	160	126	130	144	166	191	191	188	190	251	251	198	212	139	145	149	163
NBCR08	234	234	175	187	210	214	144	158	122	122	144	168	191	191	194	196	251	253	198	198	139	145	149	165
NBCR09	246	246	189	189	210	212	156	158	132	136	166	166	191	191	204	206	251	251	214	214	139	147	165	165
NBCR10	214	214	175	187	202	208	160	174	130	130	166	168	189	191	198	206	251	251	214	214	141	145	165	165
NBCR11	226	236	173	189	194	208	140	158	118	122	166	168	191	191	202	202	253	257	212	212	139	145	163	163
NBCR12	236	236	175	189	206	210	158	158	122	126	166	166	191	193	200	206	257	257	216	218	139	145	167	169
NBCR13	238	238	187	189	204	208	158	176	122	126	162	166	191	191	190	194	253	253	212	214	139	145	163	165
NBCR14	238	238	187	191	206	212	158	174	118	120	164	166	191	193	188	196	251	253	214	214	139	147	165	165
NBCR15	238	238	175	187	192	200	168	168	106	120	164	170	189	195	160	200	253	257	210	216	139	147	161	167
NBCR16	238	238	175	189	200	206	172	176	120	124	162	166	189	195	194	194	251	255	198	214	139	147	149	165
NBCR17	222	222	189	191	192	208	160	174	122	124	166	168	189	195	190	194	251	251	198	214	139	147	149	165
NBCR18	222	224	189	191	192	208	158	174	126	126	166	170	191	193	192	192	251	251	214	214	139	145	165	165
NBCR19	224	224	187	189	172	190	160	174	126	126	162	166	191	193	200	200	251	251	198	214	139	145	149	165
NBCR20	222	224	187	189	192	210	158	182	122	122	166	168	191	193	198	198	251	251	214	216	139	145	165	167
NBCR21	222	222	175	187	192	210	174	174	130	132	154	168	191	193	198	198	251	251	214	214	137	145	165	165
NBCR22	222	222	175	187	206	206	174	174	130	132	158	172	191	193	204	206	253	253	214	214	139	145	165	165
NBCR23	214	214	175	187	196	208	160	174	130	132	164	168	191	193	188	198	253	253	212	216	139	145	163	167
NBCR24	222	222	187	189	208	218	158	174	130	132	164	168	189	193	188	188	251	251	214	216	139	145	165	167
NBCR25	246	246	187	189	208	208	166	166	130	132	168	172	195	195	170	188	251	251	214	214	139	145	165	165
NBCR26	222	222	187	189	206	216	164	166	130	132	166	168	191	193	190	190	253	255	214	214	139	145	165	165
NBCR27	222	224	175	187	182	216	166	166	130	132	166	166	189	193	190	190	255	255	214	214	139	145	165	165
NBCR28	230	246	175	189	216	218	146	162	120	124	168	168	191	191	194	194	253	253	214	214	139	145	165	165
NBCR29	222	222	189	191	218	220	160	160	122	124	168	170	191	191	190	192	253	253	216	216	135	145	167	167
NBCR30	246	246	187	189	194	210	174	176	130	132	168	168	191	193	160	200	253	253	214	216	139	145	165	167
NBCR31	222	222	175	185	188	192	158	166	120	124	168	170	189	191	176	190	253	253	198	214	139	145	149	165
NBCR32	244	244	187	187	208	210	144	158	122	124	156	156	189	191	190	196	253	253	214	214	139	145	165	165
NBCR33	220	220	189	193	206	210	170	172	130	132	166	166	191	191	192	192	255	257	214	214	139	147	165	165
NBCR34	246	246	175	187	190	208	172	180	120	124	166	166	189	191	188	188	253	253	198	214	139	145	149	165
NBCR35	214	214	175	189	176	176	146	174	122	124	166	166	187	191	188	188	253	253	0	0	139	145	0	0
NBCR36	218	230	175	187	198	208	158	158	130	132	156	166	199	199	188	188	253	253	214	214	135	145	165	165
NBCR37	224	230	175	187	208	210	158	158	120	124	168	168	197	197	190	190	253	255	212	214	139	145	163	165
NBCR38	214	214	175	189	180	188	158	158	122	124	156	166	189	191	192	192	253	253	214	214	139	145	165	165
NBCR39	246	246	187	189	210	210	158	180	130	132	166	168	189	191	192	198	253	253	198	214	135	145	149	165
NBCR40	246	246	189	189	208	218	158	160	120	124	168	168	195	197	192	192	253	253	214	218	139	145	165	169
NBCR41	246	246	187	191	202	204	158	160	122	124	166	168	175	195	160	200	253	255	198	214	139	145	149	165
NBCR42	246	246	189	191	182	206	156	158	128	136	164	168	191	199	202	204	253	255	216	216	139	145	167	167
NBCR43	246	246	189	189	188	210	158	158	122	122	164	168	191	193	202	202	253	255	212	214	139	145	163	165
NBCR44	222	222	187	187	210	212	158	158	122	122	166	166	193	193	202	202	251	251	214	214	139	145	165	165
NBCR45	222	222	177	177	210	220	154	158	130	130	164	168	195	195	202	202	253	253	198	214	139	145	149	165
NBCR46	214	214	177	177	208	208	154	158	128	132	156	166	193	193	202	202	253	255	198	214	139	145	149	165
NBCR47	214	214	175	189	208	210	158	160	126	128	156	166	195	195	190	190	251	251	214	216	135	147	165	167
NBCR48	238	238	187	187	208	210	158	160	124	128	166	168	191	191	202	204	253	255	198	214	139	145	149	165
NBCR49	244	244	187	187	210	210	158	174	122	122	164	166	191	193	198	200	251	251	214	216	139	145	165	167
NBCR50	232	236	187	187	208	220	158	160	122	122	166	168	191	191	160	200	253	253	214	218	139	145	165	169
NBOP01	230	234	185	187	208	212	172	174	158	176	164	166	193	195	196	196	251	251	214	214	139	147	165	165
NBOP02	230	230	185	187	210	216	158	168	132	136	164	166	191	193	190	190	251	251	216	216	137	145	167	167
NBOP03	230	232	185	187	208	210	158	174	130	132	164	166	191	193	188	202	251	255	170	214	139	145	165	165
NBOP04	232	232	185	187	208	210	156	160	134	138	166	168	193	193	196	200	251	251	198	214	139	145	149	165
NBOP05	232	232	175	189	208	212	158	174	152	158	166	168	191	195	188	198	253	253	198	214	139	145	149	165
NBOP06	232	232	175	185	208	210	156	158	130	134	166	168	191	191	192	194	251	251	212	216	137	145	163	167
NBOP07	232	234	175	185	202	206	158	160	118	130	164	168	191	211	188	190	251	253	198	212	139	145	149	163
NBOP08	232	234	175	185	208	210	160	178	128	132	166	168	191	193	194	196	251	253	198	198	139	145	149	151
NBOP09	232	246	185	189	208	214	162	174	120	130	166	168	191	193	204	206	251	251	214	214	139	147	165	165
NBOP10	214	214	175	187	186	210	158	172	134	136	164	166	191	193	198	206	251	251	214	214	141	145	165	165
NBOP11	232	236	173	189	210	214	158	174	124	132	164	166	191	193	202	202	253	257	212	212	139	145	163	163
NBOP12	236	236	175	189	208	212	176	178	110	130	166	168	189	191	200	206	257	257	216	218	139	145	167	169
NBOP13	238	238	187	189	206	212	158	160	128	130	166	170	175	191	190	202	253	253	212	214	135	145	163	165
NBOP14	238	238	187	191	204	208	160	178	132	134	164	166	189	191	188	196	251	253	214	214	139	147	165	165
NBOP15	238	238	185	187	208	212	158	162	132	134	166	168	191	193	160	200	253	257	210	216	139	147	161	167
NBOP16	238	238	175	189	208	212	162	172	118	134	164	168	193	193	194	194	253	255	198	214	139	147	149	165
NBOP17	222	232	189	191	190	206	162	176	148	152	166	168	191	193	190	194	253	255	198	214	139	147	149	165
NBOP18	222	224	189	191	188	190	144	158	110	130	166	168	191	193	188	192	253	255	214	214	139	145	165	165
NBOP19	224	224	187	189	188	206	158	176	134	136	166	168	191	193	192	200	251	251	198	214	139	145	149	165
NBOP20	222	224	187	189	190	194	158	172	106	130	166	168	189	193	198	198	253	253	214	216	139	145	165	167
NBOP21	222	222	185	187	190	194	158	170	142	144	166	170	191	191	198	198	251	251	214	214	137	145	165	165
NBOP22	222	222	187	187	200	208	106	160	130	142	164	166	189	191	204	206	253	253	214	214	139	147	165	165
NBOP23	214	214	175	187	210	212	158	168	128	130	166	170	189	191	188	198	253	253	212	216	139	147	163	167
NBOP24	222	222	187	189	172	178	106	158	144	146	164	168	189	191	188	188	251	251	214	216	139	145	165	167
NBOP25	246	246	187	189	194	206	158	178	130	132	164	168	191	191	188	188	251	251	214	214	139	145	165	165
NBOP26	222	222	187	189	208	214	106	158	128	130	164	166	191	193	190	190	253	255	214	214	139	145	165	165
NBOP27	222	224	175	187	206	208	106	158	128	134	166	170	191	201	190	190	255	255	214	214	139	145	165	165
NBOP28	230	246	175	189	192	208	106	160	128	128	164	166	191	193	194	194	253	253	214	214	139	141	165	165
NBOP29	222	222	189	191	202	206	156	158	134	138	164	168	189	191	192	192	253	253	216	216	137	145	167	167
NBOP30	246	246	187	189	202	208	170	174	106	130	164	166	191	193	160	200	253	253	214	216	139	145	165	167
NBOP31	222	222	175	185	190	194	158	174	130	130	166	168	189	191	176	190	251	253	198	214	139	145	149	165
NBOP32	244	244	187	187	210	214	160	176	136	146	164	168	193	201	190	196	251	253	214	214	139	145	165	165
NBOP33	232	234	189	193	186	188	162	172	128	130	166	168	191	193	192	192	251	257	214	214	139	147	165	165
NBOP34	232	234	175	187	190	192	156	158	132	136	164	168	191	191	188	188	253	253	198	214	139	145	149	165
NBOP35	232	246	175	189	192	196	158	172	118	132	164	166	191	191	188	188	253	253	0	0	139	145	0	0
NBOP36	214	214	187	187	188	216	160	176	132	136	164	168	191	193	188	188	253	253	214	214	135	145	165	165
NBOP37	232	236	187	187	190	210	160	176	132	134	164	166	191	191	186	190	253	255	212	214	139	145	163	165

NBOP38	236	236	175	189	204	210	106	158	132	136	164	168	193	193	192	192	251	253	214	214	139	145	165	165
NBOP39	238	238	187	189	210	214	144	160	132	134	166	168	193	193	192	198	253	253	198	214	139	145	149	165
NBOP40	238	238	189	189	208	208	158	176	132	132	166	170	193	193	192	192	253	253	214	218	139	145	165	169
NBOP41	238	238	187	191	200	204	158	174	130	134	164	166	191	191	160	200	253	255	198	214	139	145	149	165
NBOP42	246	246	189	191	174	178	106	158	118	150	164	168	189	193	202	204	253	255	216	216	139	145	167	167
NBOP43	246	246	189	189	174	210	160	178	134	136	166	168	189	191	202	202	253	255	212	214	139	145	163	165
NBOP44	222	222	187	187	200	204	160	174	108	134	166	168	193	193	200	202	251	251	214	214	139	145	165	165
NBOP45	222	222	177	177	206	208	160	176	130	154	166	168	191	191	200	202	253	253	198	214	139	145	149	165
NBOP46	214	214	175	177	190	194	158	174	120	134	162	166	191	191	202	202	253	255	198	214	139	145	149	165
NBOP47	214	214	175	189	190	212	162	172	134	134	166	168	193	193	186	190	251	251	214	216	135	145	165	167
NBOP48	238	238	187	187	190	192	162	182	134	138	164	168	193	193	202	204	253	255	198	214	139	145	149	165
NBOP49	244	244	187	187	202	204	160	176	136	144	162	166	193	193	198	200	251	251	214	216	135	145	165	167
NBOP50	232	236	187	187	190	212	160	174	122	138	166	168	193	193	160	200	253	253	214	218	135	145	165	169
NSSMB01	230	236	183	185	204	206	172	174	134	136	154	178	191	201	196	210	251	251	214	214	139	145	165	165
NSSMB02	230	230	181	185	208	210	158	168	124	130	154	154	193	193	194	206	253	253	214	214	139	145	165	165
NSSMB03	230	230	185	185	206	208	156	160	130	136	182	182	191	191	196	212	253	269	214	216	139	145	165	167
NSSMB04	230	230	185	185	206	210	156	160	128	132	154	156	191	201	206	206	253	271	214	216	139	145	165	167
NSSMB05	230	230	181	185	206	210	156	158	132	134	134	154	191	191	196	196	253	253	214	216	139	145	165	167
NSSMB06	230	234	185	185	206	210	160	172	136	138	154	154	195	195	194	194	251	253	214	214	137	145	165	165
NSSMB07	230	230	183	185	206	210	158	162	136	138	152	152	191	191	196	204	253	267	214	214	139	145	165	165
NSSMB08	230	232	183	183	182	212	158	172	132	134	150	154	193	193	200	212	253	267	214	216	139	145	165	167
NSSMB09	228	230	181	183	208	208	160	172	134	138	152	152	191	193	196	204	251	255	214	214	137	141	165	165
NSSMB10	232	232	183	185	204	210	160	166	132	132	154	154	191	191	196	204	253	269	214	214	139	145	165	165
NSSMB11	232	232	183	185	206	210	158	174	128	130	150	158	193	193	196	204	253	253	214	214	139	145	165	165
NSSMB12	232	232	183	185	206	210	168	174	130	132	154	154	191	193	198	200	253	253	214	214	139	145	165	165
NSSMB13	232	232	183	191	174	210	158	174	130	132	154	154	191	191	206	212	253	253	214	216	139	145	165	167
NSSMB14	232	238	185	185	174	188	158	162	128	132	154	154	191	193	212	212	253	269	214	214	139	145	165	165
NSSMB15	232	232	183	185	188	192	158	160	128	134	154	154	197	201	196	204	255	271	214	214	141	141	165	165
NSSMB16	230	230	183	183	188	188	156	174	130	134	154	154	191	191	204	204	253	269	214	214	139	145	165	165
NSSMB17	232	232	183	185	188	188	158	158	130	134	154	154	191	193	194	196	253	253	214	214	139	145	165	165
NSSMB18	232	238	183	183	200	210	162	172	134	142	150	154	189	191	192	194	255	255	214	214	139	145	165	165
NSSMB19	226	232	185	185	200	210	158	164	130	134	154	154	189	191	194	206	253	253	216	216	145	145	167	167
NSSMB20	222	232	181	185	200	210	158	174	132	134	154	154	191	191	196	198	253	253	214	216	139	145	165	167
NSSMB21	234	234	183	185	176	200	160	174	136	140	152	154	191	191	200	200	253	253	214	216	139	145	165	167
NSSMB22	232	232	183	183	198	200	162	170	140	142	154	156	189	191	198	204	253	269	214	214	139	145	165	165
NSSMB23	232	232	183	185	206	208	158	162	134	136	154	156	189	193	196	196	253	269	214	214	135	145	165	165
NSSMB24	222	232	183	185	202	208	158	162	132	134	154	154	191	193	198	204	253	269	214	214	139	145	165	165
NSSMB25	232	232	181	183	188	192	160	176	134	136	154	156	189	193	196	208	253	253	214	214	139	145	165	165
NSSMB26	230	236	183	185	206	208	158	172	134	134	154	154	189	193	196	200	253	253	214	216	139	145	165	167
NSSMB27	232	234	181	183	188	208	164	164	134	138	152	158	189	193	200	204	253	253	214	240	139	145	165	165
NSSMB28	230	234	183	185	204	206	160	164	132	136	154	158	189	193	194	194	253	253	214	216	139	145	165	167
NSSMB29	230	232	181	183	204	208	156	164	132	132	134	156	191	195	196	204	253	253	214	286	139	145	165	165
NSSMB30	230	232	183	185	204	208	160	164	132	134	152	158	189	193	194	196	253	253	214	214	137	141	165	165
NSSMB31	230	230	185	185	188	208	172	176	134	136	152	154	189	193	194	204	253	253	214	216	139	145	165	167
NSSMB32	230	232	183	185	200	202	164	168	130	130	152	154	191	195	196	204	253	269	214	216	139	145	165	167
NSSMB33	228	232	183	185	184	204	160	166	132	134	152	156	189	193	194	204	253	253	214	216	139	145	165	167
NSSMB34	230	230	183	183	202	204	158	168	134	138	158	178	193	195	194	202	255	271	214	214	139	145	165	165
NSSMB35	230	230	181	183	208	208	158	172	134	134	134	156	195	195	194	200	253	253	216	216	139	145	167	167
NSSMB36	230	230	183	183	188	204	158	174	120	132	154	156	191	195	198	204	253	267	214	216	139	145	165	167
NSSMB37	230	230	169	185	188	204	156	174	134	134	154	156	189	193	196	196	253	269	214	214	139	145	165	165
NSSMB38	228	228	169	185	200	204	158	158	134	134	154	154	191	193	194	196	253	269	216	218	139	145	167	169
NSSMB39	228	228	169	183	188	188	158	176	132	132	154	154	195	195	196	204	253	253	214	214	139	145	165	165
NSSMB40	228	228	181	183	188	204	156	158	128	130	154	156	191	193	196	196	253	267	214	214	139	145	165	165
NSSMB41	228	232	183	185	188	188	158	174	132	134	152	152	189	191	196	198	255	269	214	216	137	145	165	167
NSSMB42	228	230	183	185	188	188	170	174	132	134	154	154	189	191	196	198	255	269	214	214	137	145	165	165
NSSMB43	230	230	183	183	210	210	174	186	130	134	154	154	191	191	196	198	255	269	214	214	139	145	165	165
NSSMB44	230	230	171	171	174	174	156	186	130	130	154	154	189	191	200	200	255	269	216	216	139	145	167	167
NSSMB45	230	230	185	193	190	190	156	186	130	132	152	152	189	191	202	204	253	269	214	216	139	145	165	167
NSSMB46	228	228	185	185	190	190	158	172	130	132	154	154	193	195	194	198	253	253	214	214	137	137	165	165
NSSMB47	228	228	185	187	206	208	156	156	128	132	142	156	189	193	196	198	253	253	216	216	139	145	167	167
NSSMB48	228	234	185	185	188	190	158	158	132	132	154	154	191	195	196	200	253	269	214	214	139	145	165	165
NSSMB49	228	230	183	183	190	190	158	172	128	130	154	154	195	195	196	206	253	269	214	214	139	145	165	165
NSSMB50	228	228	181	183	208	210	168	172	130	132	154	154	191	191	208	210	253	267	214	214	139	145	165	165
NSRL01	228	228	183	185	204	208	160	160	134	136	166	168	191	193	196	196	255	259	212	212	139	145	163	163
NSRL02	228	228	183	191	182	208	160	162	128	152	168	168	191	193	184	186	257	257	212	212	137	145	163	163
NSRL03	228	228	185	185	186	208	162	164	128	128	166	166	191	193	186	196	255	259	212	212	139	145	163	163
NSRL04	226	226	185	187	212	212	160	176	128	130	166	166	191	191	184	184	255	259	212	212	139	145	163	163
NSRL05	228	228	185	187	182	212	160	160	132	136	164	166	191	191	194	196	255	259	212	212	137	145	163	163
NSRL06	222	228	185	185	202	210	160	160	128	130	166	168	189	193	194	196	257	257	212	212	139	145	163	163
NSRL07	228	228	185	187	208	208	160	162	122	126	166	166	191	191	192	196	257	273	212	212	139	145	163	163
NSRL08	228	228	183	185	182	182	160	174	128	130	166	166	191	201	182	196	257	273	212	212	139	145	163	163
NSRL09	228	228	185	185	204	208	162	166	128	134	166	166	191	201	184	184	255	273	212	214	141	145	163	165
NSRL10	228	228	183	187	212	212	162	176	130	134	166	166	191	193	186	186	257	273	214	216	139	145	165	167
NSRL11	222	222	185	185	182	206	162	162	130	132	166	166	191	191	184	188	255	259	214	216	139	145	165	167
NSRL12	228	228	185	187	202	208	160	160	130	132	166	166	191	195	192	196	255	259	214	214	139	145	165	165
NSRL13	228	228	185	187	204	208	160	160	128	130	166	166	193	193	192	194	257	259	212	214	141	145	163	165
NSRL14	228	232	185	185	204	208	162	162	126	130	166	166	193	193	192	198	257	273	214	214	139	145	165	165
NSRL15	228	234	185	193	182	206	158	160	122	128	166	166	193	193	184	184	255	261	214	214	139	145	165	165
NSRL16	232	232	165	185	204	210	178	178	128	130	166	166	193	195	184	198	255	273	214	214	139	145	165	165
NSRL17	228	228	183	185	182	202	162	162	128	130	166	166	193	193	184	202	255	259	214	216	137	145	165	167
NSRL18	228	228	183	183	170	208	162	170	132	142	148	166	193	193	186	192	235	257	212	214	139	145	163	165
NSRL19	228	228	173	173	182	202	162	164	130	134	166	166	193	203	202	204	255	259	214	214	139	145	165	165
NSRL20	228	228	185	193	208	208	160	164	130	132	166	166	193	195	184	196	255	259	214	214	139	145	165	165
NSRL21	228	228	183	183	182	212	160	176	128	128	148	166	193	193	184	204	235	257	214	216	139	145	165	167
NSRL22	228	228	191	191	176	214	162	162	132	136	166	168	193	195	194	194	255	273	214	214	137	145	165	165
NSRL23	228	228	183	191	206	208	162	162	126	130	166	166	195	195	186	198	255	255	214	214	139	145	165	165
NSRL24	228	228	183	191	210	222	178	178	128	132	166	166	197	197	184	186	255	255	214	214	139	145	165	165
NSRL25	228	234	185	187	168	208	160	162	132	136	166	166	195	197	198	198	257	257	212	216	139	145	163	167
NSRL26	228	228	185	189	182	182	160	160	126	130	148	166	195	195	198	198	255	255	212	214	139	145	163	165
NSRL27	222	222	185	185	182	184	160	162	128	130	166	166	195	197	188	198	257	259	214	214	137	145	165	165
NSRL28	228	228	185	185	182	208	160	160	134	134	168	168	197	199	186	198	257	261	214	214	139	145	165	165
NSRL29	226	230	185	185	218	222	160	172	128	130	166	168	195	197	186	206	235	259	212	214	139	145	163	165
NSRL30	228	228	187	187	192	212	160	172	134	134	168	168	199	199	186	198	235	257	214	214	139	145	165	165
NSRL31	228	228	185	185	210	210	176	178	130	132	168	168	197	197	198	198	257	257	214	214	139	145	165	165
NSRL32	228	228	185	193	170	206	160	176	128	130	168	168	197	199	186	194	257	257	212	214	139	145	163	165
NSRL33	222	228	185	185	168	212	162	174	126	128	166	170	197	197	188	188	235	257	214	216	137	145	165	167
NSRL34	222	228	185	193	206	212	160	176	128	130	166	170	197	197	188	188	255	259	214	216	139	145	165	167
NSRL35	228	228	185	185	194	200	172	178	128	130	168	168	197	199	188	190	235	259	212	214	139	145	163	165
NSRL36	228	228	185	185	206	212	160	160	128	130	166	168	197	197	186	186	257	273	214	214	139	145	165	165
NSRL37	228	228	183	189	206	212	160	176	128	130	168	168	199	199	190	194	259	259	214	214	139	145	165	165
NSRL38	228	234	183	193	168	212	160	174	102	130	168	170	199	199	192	194	257	257	214	214	137	145	165	165
NSRL39	234	234	183	193	206	212	160	160	128	130	166	166	199	201	196	196	257	257	214	216	139	145	165	167
NSRL40	228	228	185	187	206	212	160	160	130	130	168	168	199	201	242	242	257	257	214	216	139	145	165	167
NSRL41	228	228	185	193	206	214	170	176	126	130	168	168	199	199	202	202	257	273	216	216	139	145	167	167
NSRL42	228	228	185	189	168	212	158	174	124	126	146	168	195	199	186	186	257	259	214	214	137	137	165	165
NSRL43	228	228	185	193	182	182	158	178	124	128	170	170	197	197	188	188	257	273	214	214	139	145	165	165
NSRL44	222	228	185	187	168	212	158	168	124	124	150	168	195	197	188	200	235	259	214	216	139	145	165	167
NSRL45	228	228	185	187	182	202	158	158	128	130	166	168	197	199	186	186	259	273	214	214	139	145	165	165
NSRL46	228	228	193	195	208	212	158	160	128	136	168	168	199	201	196	198	257	257	212	214	137	141	163	165
NSRL47	228	228	193	195	208	208	156	156	126	126	168	168	199	207	194	194	257	257	214	214	139	145	165	165
NSRL48	228	234	191	193	206	212	156	156	130	134	168	168	199	207	186	186	255	259	214	214	139	145	165	165
NSRL49	228	228	185	187	206	212	156	174	0	0	170	170	197	197	192	198	235	257	214	216	139	141	165	167
NSRL50	228	228	185	187	206	210	156	156	134	138	152	168	195	197	192	198	235	259	214	216	139	145	165	167
NSDL01	226	226	183	183	192	210	154	154	126	130	166	168	189	189	186	194	249	253	214	216	139	141	165	167
NSDL02	226	226	181	187	178	178	142	150	126	130	166	168	191	191	188	192	251	251	214	218	139	145	165	169
NSDL03	226	228	185	187	208	214	140	140	132	132	166	166	189	189	188	190	249	251	216	216	139	145	167	167
NSDL04	226	228	185	187	188	208	138	138	128	128	166	166	189	191	190	192	251	251	218	218	137	145	169	169
NSDL05	228	228	179	185	210	210	154	154	128	130	166	168	191	191	190	192	251	253	218	218	139	145	169	169
NSDL06	226	226	175	185	176	208	154	154	130	132	166	166	187	191	188	192	253	255	214	216	139	145	165	167
NSDL07	228	228	183	185	206	210	138	138	130	132	166	166	191	191	180	194	251	253	216	220	137	145	167	171
NSDL08	226	228	181	181	186	208	138	152	130	130	166	166	189	189	192	198	251	253	214	216	139	145	165	167
NSDL09	236	236	183	183	208	208	138	138	126	126	166	166	189	191	196	198	251	255	216	216	139	145	167	167
NSDL10	228	230	185	185	206	208	138	180	126	140	166	166	191	191	196	198	253	253	214	214	139	145	165	165

NSDL11	228	228	183	187	190	212	142	152	128	128	166	184	187	191	198	204	253	255	214	214	139	145	165	165
NSDL12	228	230	183	187	202	202	138	138	128	128	166	166	187	191	194	198	253	255	214	216	139	145	165	167
NSDL13	228	230	185	185	208	210	138	138	124	130	166	166	191	191	190	196	251	253	194	214	139	145	165	165
NSDL14	228	228	183	183	206	208	148	148	126	144	166	166	189	189	188	192	253	255	216	216	135	145	167	167
NSDL15	228	230	181	181	186	186	136	140	128	128	166	166	187	191	186	190	251	253	214	216	139	145	165	167
NSDL16	228	230	183	183	190	190	138	154	124	126	164	166	189	191	186	188	253	253	212	214	139	145	163	165
NSDL17	228	230	183	183	182	210	136	154	126	126	166	166	187	189	190	190	255	255	214	214	139	145	165	165
NSDL18	226	226	185	189	206	210	142	142	126	128	164	164	189	193	190	194	255	255	214	216	135	141	165	167
NSDL19	228	234	179	185	190	190	140	140	126	128	166	166	189	189	184	190	253	255	214	216	139	145	165	167
NSDL20	228	238	183	183	202	210	140	146	144	144	166	166	189	189	190	198	253	255	212	216	139	145	163	167
NSDL21	230	234	183	183	204	206	140	142	110	130	166	166	189	199	190	194	253	253	212	216	139	145	163	167
NSDL22	230	234	183	183	206	206	140	140	126	126	166	166	189	199	188	194	251	253	214	214	139	145	165	165
NSDL23	228	232	181	181	206	206	140	140	126	128	166	166	191	191	182	190	251	253	214	216	135	145	165	167
NSDL24	228	228	167	181	182	210	138	154	130	132	168	168	189	191	188	192	251	253	214	214	139	145	165	165
NSDL25	226	230	187	189	190	190	148	152	126	130	168	168	189	193	196	198	251	253	212	216	139	145	163	167
NSDL26	228	230	183	183	184	188	140	152	126	130	166	168	189	189	190	196	247	255	214	216	139	145	165	167
NSDL27	228	230	183	187	192	212	140	148	124	130	166	166	189	189	190	194	251	253	214	214	139	141	165	165
NSDL28	228	230	183	183	210	210	140	140	124	126	166	166	187	189	190	194	251	253	214	216	139	141	165	167
NSDL29	228	230	185	187	210	210	140	140	124	126	166	166	187	189	188	192	253	253	214	214	139	145	165	165
NSDL30	226	230	183	183	192	212	144	152	124	128	166	166	189	191	188	188	253	253	214	216	139	145	165	167
NSDL31	228	228	185	189	210	210	140	154	110	130	166	184	189	189	190	194	251	253	214	216	139	145	165	167
NSDL32	226	238	185	189	206	210	140	140	122	122	170	170	189	199	190	194	251	253	214	214	137	145	165	165
NSDL33	226	228	181	181	206	206	140	142	128	128	166	166	191	191	190	194	251	253	214	216	139	145	165	167
NSDL34	226	228	183	183	212	212	140	140	126	126	166	166	189	189	192	198	253	261	214	216	139	145	165	167
NSDL35	240	242	181	185	208	208	140	140	124	126	166	166	189	191	194	196	253	253	216	216	139	145	167	167
NSDL36	240	242	183	187	204	204	140	142	130	130	166	166	191	191	192	194	253	255	216	216	139	145	167	167
NSDL37	240	242	183	187	208	214	140	142	128	130	166	166	191	191	190	194	253	253	214	218	135	145	165	169
NSDL38	240	242	183	183	212	212	140	140	138	138	166	166	189	189	192	194	251	253	216	218	139	145	167	169
NSDL39	240	242	183	187	192	210	140	140	126	128	166	166	189	189	190	196	251	253	214	218	139	145	165	169
NSDL40	240	242	183	187	210	212	140	152	130	130	166	166	189	189	188	192	253	253	216	222	137	145	167	173
NSDL41	228	228	183	187	204	204	140	140	122	122	166	166	189	189	194	198	251	253	214	216	139	145	165	167
NSDL42	226	230	185	185	208	212	144	154	194	194	166	166	191	193	192	196	253	255	216	216	137	145	167	167
NSDL43	228	232	183	185	194	210	140	150	128	130	166	166	191	191	192	196	251	253	214	218	139	145	165	169
NSDL44	228	230	183	183	190	214	138	140	124	126	166	166	189	191	188	204	253	255	216	216	139	145	167	167
NSDL45	228	228	183	185	212	212	140	146	126	128	166	166	193	193	192	196	251	255	216	216	139	145	167	167
NSDL46	228	228	185	191	220	220	140	140	132	132	166	166	191	191	200	204	253	255	216	216	139	145	167	167
NSDL47	228	228	181	181	212	212	142	154	132	132	164	164	191	193	190	194	253	255	218	218	139	145	169	169
NSDL48	228	228	181	185	204	210	140	140	124	124	166	166	191	193	190	194	251	253	216	216	137	145	167	167
NSDL49	228	230	179	183	204	204	142	140	132	132	166	166	191	191	192	198	253	255	218	218	139	145	169	169
NSDL50	228	230	181	181	192	208	140	140	128	128	168	168	191	191	192	194	255	257	216	216	139	145	167	167
NSUM01	230	236	187	199	210	210	160	160	130	134	182	182	191	191	190	192	251	251	214	216	137	145	165	167
NSUM02	230	230	187	199	200	210	158	162	130	134	182	182	187	187	190	190	251	251	214	216	139	145	165	167
NSUM03	230	230	181	183	206	210	160	176	130	132	182	182	187	187	188	198	251	267	214	214	139	145	165	165
NSUM04	230	230	181	185	202	204	160	176	132	136	182	184	191	191	188	190	251	269	214	214	139	145	165	165
NSUM05	230	230	181	183	210	210	162	162	130	134	180	180	191	191	190	190	251	251	212	214	139	145	163	165
NSUM06	230	234	179	183	206	206	160	172	128	130	180	180	187	189	188	190	251	251	212	214	139	145	163	165
NSUM07	230	230	181	183	190	216	158	160	128	132	182	182	187	187	188	194	251	267	212	214	139	145	163	165
NSUM08	230	232	181	183	200	204	156	160	130	132	180	182	187	187	190	194	251	267	214	214	135	145	165	165
NSUM09	230	230	183	185	194	202	156	160	128	134	180	182	187	187	188	188	251	251	212	214	137	145	163	165
NSUM10	232	232	183	185	206	206	158	158	128	134	182	182	189	189	190	190	253	267	212	214	135	145	163	165
NSUM11	232	232	183	185	194	216	160	176	126	130	180	180	187	187	194	194	253	253	212	214	135	145	163	165
NSUM12	232	232	183	183	194	216	160	160	128	132	182	182	187	189	190	198	253	253	210	214	139	145	161	165
NSUM13	232	232	181	183	190	218	176	176	130	130	180	180	189	189	188	188	253	271	214	214	139	145	165	165
NSUM14	232	238	183	185	196	196	160	176	130	134	178	180	189	199	196	198	253	253	212	214	139	145	163	165
NSUM15	232	232	183	185	206	206	160	160	132	136	180	180	189	199	188	198	253	269	210	214	137	145	161	165
NSUM16	230	232	183	185	208	210	160	160	132	134	180	180	187	189	190	198	255	271	214	216	139	145	165	167
NSUM17	232	232	183	185	208	210	160	174	132	134	178	178	185	199	188	196	255	255	212	216	139	145	163	167
NSUM18	232	238	181	183	208	208	158	174	132	134	176	178	185	201	188	192	255	255	212	214	139	145	163	165
NSUM19	228	232	185	185	188	210	158	160	134	134	176	178	185	185	192	198	255	255	214	216	139	145	165	167
NSUM20	222	232	183	183	174	206	160	174	132	134	178	180	185	185	194	200	255	255	212	214	139	145	163	165
NSUM21	234	234	183	185	206	210	160	160	130	130	176	178	185	199	188	194	255	255	214	214	137	145	165	165
NSUM22	232	232	185	185	206	210	158	158	130	130	178	178	185	185	188	188	255	271	214	214	139	145	165	165
NSUM23	232	232	183	183	206	206	158	158	128	130	154	178	155	187	188	192	255	271	214	216	139	145	165	167
NSUM24	232	232	165	173	206	206	158	158	130	132	178	180	185	187	190	192	255	271	214	214	137	145	165	165
NSUM25	232	232	183	183	210	210	160	160	134	134	178	178	185	185	188	198	253	253	214	214	139	145	165	165
NSUM26	232	236	181	183	190	206	160	160	134	140	156	178	187	189	194	196	253	253	214	214	139	145	165	165
NSUM27	228	234	181	185	176	190	160	160	130	130	170	180	185	189	198	202	253	253	214	214	139	145	165	165
NSUM28	230	232	181	183	206	212	160	162	130	130	162	174	189	189	192	206	253	253	214	216	139	145	165	167
NSUM29	230	232	185	187	178	214	160	164	136	136	156	178	161	187	198	200	253	253	214	214	137	145	165	165
NSUM30	230	232	183	187	210	210	162	178	138	138	176	180	155	187	194	198	253	253	210	214	139	145	161	165
NSUM31	230	230	147	147	208	212	158	174	128	128	180	180	185	185	186	188	253	253	214	214	139	145	165	165
NSUM32	228	232	183	189	190	212	140	160	136	136	178	180	161	187	196	206	253	269	214	214	139	145	165	165
NSUM33	228	232	169	183	208	208	158	176	136	136	180	182	185	199	196	198	253	253	212	216	141	145	163	167
NSUM34	230	230	183	185	204	210	158	158	136	136	178	178	185	189	188	198	253	269	212	214	139	145	163	165
NSUM35	230	230	183	183	206	212	160	160	132	132	178	178	185	185	200	200	251	251	212	214	139	145	163	165
NSUM36	230	230	183	185	210	210	158	158	136	156	180	180	185	189	188	198	253	267	214	216	139	145	165	167
NSUM37	230	230	183	187	210	210	160	164	156	156	180	180	185	189	194	194	251	267	214	214	135	145	165	165
NSUM38	228	232	183	185	208	210	158	174	130	132	180	180	189	189	190	198	253	269	212	214	139	145	163	165
NSUM39	230	230	183	185	208	210	156	160	130	132	182	182	185	185	196	196	251	251	212	214	139	145	163	165
NSUM40	230	230	183	183	190	190	158	174	130	134	178	178	183	185	190	194	253	267	214	214	139	145	165	165
NSUM41	230	232	183	183	190	210	158	174	132	136	178	182	183	185	188	198	253	267	214	216	137	141	165	167
NSUM42	230	232	185	185	198	210	158	160	136	138	170	180	185	187	188	192	253	267	214	216	139	145	165	167
NSUM43	230	232	183	187	198	210	158	174	128	128	180	180	187	199	188	190	253	267	214	214	139	145	165	165
NSUM44	230	230	183	197	190	210	158	158	128	128	180	180	185	199	190	190	253	267	214	214	139	145	165	165
NSUM45	228	230	183	197	208	210	160	168	134	136	180	180	187	189	190	198	253	267	214	214	139	145	165	165
NSUM46	224	230	185	197	210	210	160	172	136	136	182	182	187	189	190	190	251	251	214	216	139	141	165	167
NSUM47	230	230	183	185	208	218	160	160	130	134	182	182	183	187	180	188	253	253	214	214	139	145	165	165
NSUM48	228	234	181	197	208	208	160	162	132	132	182	182	185	187	194	194	253	267	214	216	139	145	165	167
NSUM49	228	230	183	183	204	208	158	174	132	134	174	182	185	187	180	188	253	267	214	214	139	145	165	165
NSUM50	230	230	183	183	208	208	158	174	126	128	182	182	185	187	192	198	253	267	214	216	137	141	165	167
PQTM01	230	234	183	183	206	206	158	158	132	134	176	178	189	191	188	188	257	257	214	216	139	145	165	167
PQTM02	230	230	181	183	196	208	144	158	126	128	176	178	189	191	190	192	257	257	214	214	145	145	165	165
PQTM03	230	232	183	185	190	206	158	158	128	130	176	178	189	191	190	190	255	257	214	216	145	145	165	167
PQTM04	232	232	183	183	172	190	160	160	128	130	174	180	189	189	190	198	253	253	210	216	139	145	161	167
PQTM05	232	232	185	185	210	210	158	174	124	126	172	176	189	191	194	198	253	253	170	214	139	145	165	165
PQTM06	232	232	183	185	206	208	164	174	124	128	174	176	177	191	202	202	253	253	198	214	139	145	149	165
PQTM07	232	234	181	181	204	212	170	170	124	128	178	180	191	193	192	198	253	255	198	214	139	145	149	165
PQTM08	234	234	185	187	196	208	158	172	124	130	168	176	175	191	186	188	255	271	212	216	141	145	163	167
PQTM09	246	246	169	185	190	208	158	172	128	130	174	176	189	191	186	188	255	255	198	212	141	145	149	163
PQTM10	214	214	185	187	206	210	158	158	130	132	176	178	189	191	188	192	255	255	198	198	139	145	149	165
PQTM11	226	236	183	183	206	206	176	176	126	128	172	176	193	201	188	190	255	255	214	214	139	145	165	165
PQTM12	236	236	183	187	206	210	158	176	126	132	170	176	191	191	160	200	255	273	214	214	139	145	165	165
PQTM13	238	238	185	187	192	202	158	176	126	132	174	176	191	191	188	198	255	255	212	212	137	145	163	163
PQTM14	238	238	183	185	208	208	158	160	126	128	174	176	191	191	192	194	253	253	216	218	139	145	167	169
PQTM15	238	238	175	187	200	206	158	162	128	134	172	174	191	191	188	190	253	253	212	214	139	145	163	165
PQTM16	238	238	175	189	192	208	160	176	128	134	174	176	191	191	194	196	253	271	214	214	135	145	165	165
PQTM17	222	222	189	191	204	208	158	174	132	134	174	176	189	191	188	194	255	269	210	216	139	145	161	167
PQTM18	222	224	189	191	206	206	162	176	130	132	170	174	191	193	190	190	267	271	198	214	139	145	149	165
PQTM19	228	228	187	189	194	194	176	176	130	132	170	174	191	191	196	196	251	251	214	216	137	145	165	167
PQTM20	228	228	187	189	196	210	160	176	118	120	172	174	189	193	190	200	255	271	212	216	139	145	163	167
PQTM21	226	228	175	187	196	212	160	160	126	132	174	178	189	191	192	194	251	269	212	216	139	145	163	167
PQTM22	228	228	175	187	196	210	158	158	130	132	170	174	177	185	188	190	255	255	214	214	141	145	165	165
PQTM23	222	228	175	187	196	196	150	150	136	138	172	174	187	191	198	198	253	277	212	218	141	145	163	169
PQTM24	228	228	187	189	208	218	158	160	128	130	172	174	189	193	188	200	253	253	214	216	141	141	165	167
PQTM25	226	228	187	189	208	212	158	160	128	138	172	208	177	187	190	190	251	251	214	214	139	145	165	165
PQTM26	226	230	187	189	206	206	160	160	126	132	172	174	189	195	192	198	251	255	214	214	139	145	165	165
PQTM27	230	246	175	187	182	208	176	176	132	134	172	200	177	185	190	198	251	251	216	216	137	145	167	167
PQTM28	228	230	175	189	206	208	160	160	130	134	172	174	189	193	192	192	251	251	214	214	139	145	165	165
PQTM29	222	222	189	191	208	210	176	176	132	134	174	176	187	191	188	200	253	257	214	214	139	145	165	165
PQTM30	222	222	169	185	206	206	158	174	122	124	170	176	185	193	188	200	253	253	214	214	135	145	165	165
PQTM31	228	228	181	183	204	208	158	164	126	128	176	178	191	193	188	190	253	253	214	214	141	145	165	165
PQTM32	228	228	181	185	202	202	174	174	128	130	170	176	191	193	190	198	255	257	214	214	139	145	165	165
PQTM33	228	228	183	185	208	208	158	158	126	128	170	172	189	193	190	190	253	253	214	214	141	145	165	165

PQTM34	228	234	183	185	208	208	158	172	124	126	176	178	195	195	194	194	253	253	216	216	139	145	167	167
PQTM35	228	234	181	183	208	210	144	158	130	132	174	176	191	193	190	192	253	255	214	216	139	145	165	167
PQTM36	214	228	169	185	208	208	168	172	124	126	170	176	189	193	160	200	253	261	198	214	141	145	149	165
PQTM37	226	228	181	183	206	210	158	162	130	132	170	176	191	191	176	190	253	255	214	214	141	145	165	165
PQTM38	226	228	183	185	204	206	160	174	126	128	172	176	191	191	188	198	253	255	214	214	141	145	165	165
PQTM39	226	228	183	185	204	208	158	158	130	132	174	176	191	193	188	200	253	255	214	226	139	145	165	177
PQTM40	226	228	185	185	208	208	158	158	126	128	202	204	189	191	188	190	253	255	216	218	139	145	167	169
PQTM41	228	228	169	181	204	210	144	158	134	136	170	176	189	191	194	200	251	251	216	220	139	145	167	171
PQTM42	228	228	185	185	190	202	158	174	126	128	174	176	189	191	204	204	253	253	218	222	141	145	169	173
PQTM43	214	228	183	183	208	210	158	178	128	130	172	178	189	191	198	200	253	255	216	222	139	145	167	173
PQTM44	228	228	185	185	190	200	158	178	128	130	174	176	191	193	202	202	251	251	218	222	139	145	169	173
PQTM45	228	228	185	185	206	208	168	168	128	132	174	176	191	191	202	202	255	255	216	218	141	145	167	169
PQTM46	228	228	177	177	204	208	170	174	128	132	174	176	191	193	190	190	255	255	216	220	139	145	167	171
PQTM47	228	228	175	189	204	204	158	170	126	128	174	176	193	193	202	204	255	261	212	214	139	145	163	165
PQTM48	228	228	187	187	208	208	172	172	128	130	174	176	195	195	198	200	255	261	214	214	141	145	165	165
PQTM49	228	228	187	187	206	206	162	172	136	138	174	176	193	193	198	200	255	261	198	214	139	145	149	165
PQTM50	228	228	185	185	206	206	172	172	122	122	174	176	195	195	198	200	255	261	198	214	139	145	149	165
PQCT01	228	228	183	183	204	204	158	158	132	132	192	192	189	191	188	188	255	257	214	216	139	145	165	167
PQCT02	228	228	181	183	194	206	158	158	136	136	192	192	189	191	190	192	257	257	214	214	139	145	165	165
PQCT03	232	232	183	185	188	204	158	158	130	130	192	192	189	191	190	190	255	273	214	216	139	145	165	167
PQCT04	228	232	183	183	170	188	160	160	132	132	190	194	189	189	190	198	255	257	210	216	137	143	161	167
PQCT05	222	228	185	185	194	206	174	174	132	132	188	190	189	191	194	198	255	271	212	216	137	145	163	167
PQCT06	228	230	183	185	204	206	164	174	128	128	190	190	177	191	202	202	255	273	214	216	137	145	165	167
PQCT07	228	230	181	181	202	210	170	170	128	130	194	194	191	193	192	198	255	257	214	216	141	145	165	167
PQCT08	226	230	185	187	194	206	158	172	128	130	184	190	175	191	186	188	257	271	206	216	141	145	157	167
PQCT09	228	230	169	185	188	206	158	172	128	132	190	190	189	191	186	188	257	255	212	216	141	145	163	167
PQCT10	228	232	185	187	204	208	158	158	132	132	192	192	191	191	188	192	255	273	214	214	141	141	165	165
PQCT11	234	234	183	183	204	204	176	176	134	134	188	190	175	191	188	190	255	255	214	214	141	145	165	165
PQCT12	228	230	183	187	204	208	182	182	130	130	186	190	177	191	198	204	269	273	212	214	141	145	163	165
PQCT13	228	232	185	187	190	200	158	176	130	134	190	190	189	195	188	200	269	269	214	214	139	145	165	165
PQCT14	228	228	183	185	206	206	158	160	130	134	190	190	189	193	190	190	253	253	214	218	139	145	165	169
PQCT15	228	228	171	183	182	184	158	162	130	130	188	188	179	193	188	198	253	257	212	214	139	145	163	165
PQCT16	228	228	183	187	190	206	160	176	132	136	190	190	193	193	192	200	253	271	212	216	141	145	163	167
PQCT17	228	228	183	183	202	206	174	174	132	136	190	190	191	199	188	194	257	269	212	218	141	145	163	169
PQCT18	228	228	183	185	204	204	162	176	136	136	186	188	191	193	190	190	267	271	214	218	139	145	165	169
PQCT19	228	228	185	191	192	192	176	176	134	134	186	188	191	191	196	196	251	251	214	216	139	145	165	167
PQCT20	228	228	183	185	194	208	160	176	134	134	188	188	189	193	190	200	269	271	212	216	135	145	163	167
PQCT21	226	228	181	185	194	210	160	160	134	134	190	192	189	191	192	194	257	269	212	216	139	145	163	167
PQCT22	228	228	183	183	194	208	158	158	130	134	186	188	177	185	188	190	255	255	214	214	141	145	165	165
PQCT23	222	228	183	183	194	194	150	150	134	134	188	188	187	191	198	198	253	277	212	218	141	145	163	169
PQCT24	228	228	183	185	198	202	158	160	140	140	188	188	189	193	188	200	253	253	214	216	141	141	165	167
PQCT25	226	228	183	185	188	188	144	160	132	132	188	222	177	187	190	190	255	257	214	214	141	145	165	165
PQCT26	226	230	183	185	204	204	160	160	132	140	188	188	189	195	192	198	253	255	214	214	139	141	165	165
PQCT27	226	228	169	183	180	206	176	176	130	134	188	214	177	185	190	198	253	271	216	216	139	145	167	167
PQCT28	228	230	183	185	204	206	160	160	136	136	188	188	189	193	192	192	253	253	214	214	141	145	165	165
PQCT29	226	230	181	181	206	208	176	176	134	136	190	190	187	191	188	200	253	257	214	214	141	145	165	165
PQCT30	228	230	169	185	204	204	174	174	136	136	186	190	185	193	188	200	257	253	214	214	139	145	165	165
PQCT31	228	228	181	183	202	206	158	164	134	134	192	192	185	193	188	190	253	253	214	216	139	145	165	167
PQCT32	228	228	181	185	200	200	174	174	130	130	186	190	187	193	190	198	255	257	212	218	139	145	163	169
PQCT33	228	228	183	185	206	206	158	158	132	132	186	186	189	189	190	190	253	253	214	216	139	145	165	167
PQCT34	228	234	183	185	206	206	158	172	130	130	192	192	189	191	192	192	253	257	214	218	141	145	165	169
PQCT35	228	234	181	183	206	208	144	158	128	128	190	190	189	191	192	194	253	253	214	218	139	145	165	169
PQCT36	228	228	169	185	206	206	168	172	134	134	186	190	189	199	188	190	253	261	214	216	141	145	165	167
PQCT37	226	228	181	183	204	208	162	162	128	128	186	190	191	191	198	198	253	255	214	216	141	145	165	167
PQCT38	226	228	183	185	202	204	160	174	134	134	188	190	191	191	188	198	255	271	214	216	141	145	165	167
PQCT39	226	228	183	185	202	206	158	158	130	130	190	190	189	193	188	200	255	255	214	226	141	145	165	177
PQCT40	226	228	185	185	206	206	158	158	134	134	218	218	191	201	188	190	255	273	216	218	139	145	167	169
PQCT41	228	228	169	181	202	208	158	158	130	130	186	190	189	191	194	200	255	269	216	220	139	145	167	171
PQCT42	228	228	185	185	188	200	174	174	138	138	190	190	189	191	204	204	255	255	218	222	139	145	169	173
PQCT43	228	228	183	183	206	208	158	178	130	130	188	192	189	191	198	200	255	255	216	222	139	145	167	173
PQCT44	228	228	185	185	188	198	158	178	132	132	190	190	191	193	198	200	255	271	218	222	139	145	169	173
PQCT45	228	228	185	185	204	206	168	168	132	132	190	190	191	191	198	200	255	269	216	218	139	145	167	169
PQCT46	228	228	185	185	202	206	170	174	132	134	190	190	191	191	198	200	255	255	216	220	139	145	167	171
PQCT47	228	228	185	185	202	202	170	170	132	134	190	190	189	189	198	200	255	261	214	216	141	145	165	167
PQCT48	228	228	185	185	206	206	172	172	130	130	190	190	191	191	198	200	255	261	212	216	141	145	163	167
PQCT49	228	228	185	185	204	204	172	172	132	132	190	190	191	193	198	200	255	261	214	216	139	145	165	167
PQCT50	228	228	185	185	204	204	172	172	140	140	190	190	195	195	198	200	255	261	214	216	139	145	165	167
PQSR01	228	230	183	183	206	206	164	176	132	132	150	150	195	195	186	190	255	257	214	214	139	141	165	165
PQSR02	226	228	183	183	196	208	162	170	132	132	162	168	195	195	186	190	257	259	214	214	139	141	165	165
PQSR03	226	230	183	183	190	206	176	176	132	132	156	170	185	191	184	192	255	259	212	212	139	145	163	163
PQSR04	228	232	185	185	172	190	156	176	128	130	156	166	189	195	190	198	255	271	212	216	137	143	163	167
PQSR05	226	230	185	185	196	208	160	170	130	132	164	168	191	191	190	198	255	259	214	214	139	145	165	165
PQSR06	228	232	183	189	206	208	160	176	132	136	166	168	191	191	184	192	255	259	214	214	141	145	165	165
PQSR07	228	232	183	183	204	212	160	170	132	132	168	168	191	197	184	192	255	257	214	214	141	145	165	165
PQSR08	230	230	183	183	196	208	174	176	132	132	156	168	189	189	186	194	253	261	214	214	141	145	165	165
PQSR09	228	232	183	183	190	208	160	160	134	134	166	166	189	191	186	192	257	273	214	214	141	145	165	165
PQSR10	226	232	183	183	206	210	162	176	132	136	166	166	189	189	188	196	255	259	214	214	139	141	165	165
PQSR11	230	230	185	185	206	206	160	176	132	132	158	166	189	189	190	196	257	273	214	214	141	145	165	165
PQSR12	230	230	183	183	206	210	160	160	134	134	166	166	189	191	190	190	255	259	214	214	141	145	165	165
PQSR13	228	230	183	183	192	202	160	160	136	144	166	166	191	195	192	192	257	257	214	214	139	145	165	165
PQSR14	228	230	183	185	208	208	160	174	136	144	166	168	193	193	192	198	255	259	214	216	139	145	165	167
PQSR15	230	230	183	183	184	186	148	160	134	136	164	166	189	191	188	190	257	293	214	214	139	145	165	165
PQSR16	228	232	183	183	192	208	160	162	134	136	166	166	191	191	196	196	255	255	214	214	141	141	165	165
PQSR17	228	230	185	185	204	208	160	160	134	136	166	166	193	193	200	204	255	261	214	216	141	141	165	167
PQSR18	228	230	185	185	206	206	160	176	132	134	154	166	187	193	188	190	255	267	214	216	139	145	165	167
PQSR19	228	228	185	187	194	194	136	140	126	134	166	166	191	191	188	198	255	259	214	214	139	145	165	165
PQSR20	228	230	169	183	196	210	158	160	130	134	166	166	191	193	188	198	257	263	214	214	135	145	165	165
PQSR21	230	230	183	185	196	212	164	176	132	136	166	166	191	193	160	192	255	259	214	214	139	145	165	165
PQSR22	228	230	181	203	196	210	174	176	130	134	156	168	189	189	198	198	255	259	214	216	141	145	165	167
PQSR23	228	230	173	203	196	210	160	174	134	134	166	166	191	191	198	198	257	257	214	216	141	145	165	167
PQSR24	226	230	183	185	200	204	162	162	130	130	166	166	197	197	192	202	255	255	216	216	139	141	167	167
PQSR25	226	228	183	183	190	190	150	152	130	130	170	180	201	203	0	0	255	255	214	214	141	145	165	165
PQSR26	228	230	183	183	206	206	160	162	132	134	170	180	197	197	188	194	253	253	214	216	139	141	165	167
PQSR27	228	228	185	185	182	208	160	176	132	132	170	178	191	191	196	200	253	253	214	214	139	145	165	165
PQSR28	228	230	183	183	206	208	162	162	134	134	176	180	191	195	192	200	253	253	216	216	141	145	167	167
PQSR29	226	232	183	183	208	210	160	160	130	130	156	168	189	189	164	208	253	253	216	216	139	145	167	167
PQSR30	228	230	183	183	206	206	162	176	132	136	168	180	191	193	168	204	253	271	216	216	141	145	167	167
PQSR31	226	230	185	185	204	208	164	164	132	132	168	172	191	207	166	200	255	269	216	216	141	145	167	167
PQSR32	228	230	183	185	202	202	160	162	132	134	168	168	201	205	166	192	267	271	214	216	139	145	165	167
PQSR33	214	228	181	183	208	208	148	160	130	136	164	166	191	191	164	194	251	251	212	214	139	145	163	165
PQSR34	228	228	183	183	208	208	176	176	130	130	166	166	191	191	162	192	255	271	214	216	141	143	165	167
PQSR35	226	228	183	183	208	210	160	170	132	134	150	150	191	191	162	192	251	269	214	214	139	145	165	165
PQSR36	226	230	183	185	208	208	160	162	132	132	166	166	201	201	160	194	255	255	214	214	141	145	165	165
PQSR37	226	228	185	185	206	210	160	162	130	132	168	168	191	201	160	194	253	277	214	214	141	143	165	165
PQSR38	228	230	183	183	204	206	162	176	108	134	166	168	191	193	160	192	253	253	214	214	141	145	165	165
PQSR39	226	230	183	185	204	208	166	176	134	134	166	166	193	193	162	194	253	253	214	216	141	141	165	167
PQSR40	228	230	181	187	208	208	174	176	142	142	166	166	191	191	194	194	253	255	214	216	139	145	165	167
PQSR41	228	228	183	183	204	210	160	162	134	134	166	166	193	193	160	194	253	253	214	214	139	145	165	165
PQSR42	228	228	183	183	190	202	166	170	134	142	166	168	191	191	160	192	253	253	214	216	139	145	165	167
PQSR43	228	228	183	183	208	210	160	162	136	136	166	166	191	191	194	200	253	257	214	214	139	145	165	165
PQSR44	228	234	183	183	190	200	162	166	132	132	166	166	191	193	164	192	253	253	214	216	139	145	165	167
PQSR45	228	234	183	183	206	208	160	178	134	134	166	166	191	191	194	208	253	253	216	216	139	145	167	167
PQSR46	228	228	185	185	204	208	160	162	132	132	166	166	191	191	190	196	255	257	216	216	139	145	167	167
PQSR47	214	228	181	183	204	204	160	178	132	132	144	166	193	201	190	190	253	253	218	216	141	145	169	167
PQSR48	228	228	183	187	208	208	158	160	130	132	166	166	191	191	186	190	253	253	214	214	141	145	165	165
PQSR49	232	232	183	183	206	206	162	162	132	132	144	166	191	191	186	190	253	255	214	214	139	145	165	165
PQSR50	226	226	183	183	206	206	166	178	140	140	168	168	191	191	190	192	253	261	214	214	139	145	165	165
PQSS01	228	228	181	183	206	210	146	162	130	130	156	166	191	199	188	194	257	257	214	214	139	145	165	165
PQSS02	228	228	181	181	172	210	148	156	130	130	158	162	187	193	194	198	257	257	214	216	139	145	165	167
PQSS03	234	234	181	183	190	214	144	158	126	132	154	164	187	195	186	188	255	273	214	214	139	145	165	165
PQSS04	216	216	181	181	182	212	144	168	130	132	160	164	193	199	188	190	257	257	216	216	137	143	167	167
PQSS05	222	228	183	191	192	208	154	154	130	132	162	166	191	197	196	198	255	255	214	216	137	145	165	167
PQSS06	228	228	183	187	208	210	156	156	130	132	154	164	193	195	190	206	255	273	212	214	137	145	163	165

PQSS07	228	228	175	185	212	212	152	162	120	134	164	164	187	193	186	190	255	255	212	216	141	145	163	167
PQSS08	226	228	183	183	208	210	154	160	120	130	160	166	187	193	190	194	255	271	212	214	141	145	163	165
PQSS09	228	228	183	183	206	210	142	162	132	132	154	164	193	203	190	194	255	255	212	214	141	145	163	165
PQSS10	228	234	175	185	210	214	158	162	132	132	162	164	187	193	188	200	255	255	212	214	141	141	163	165
PQSS11	234	234	183	183	210	210	156	156	120	130	158	166	193	197	188	190	255	255	214	216	141	145	165	167
PQSS12	228	228	183	185	194	194	144	158	130	132	158	158	191	193	188	192	255	273	214	214	141	145	165	165
PQSS13	228	248	183	183	190	210	152	174	130	130	160	160	205	209	190	196	255	255	214	214	139	145	165	165
PQSS14	228	228	183	183	210	210	144	154	128	132	156	166	191	195	190	194	255	255	214	214	139	145	165	165
PQSS15	228	228	175	175	190	212	144	156	120	136	164	164	193	207	190	194	255	255	214	216	139	145	165	167
PQSS16	228	228	183	185	196	198	142	176	130	130	154	166	193	193	190	202	255	255	214	216	141	145	165	167
PQSS17	228	228	183	183	210	214	144	156	130	132	162	166	191	191	190	192	255	255	214	214	141	145	165	165
PQSS18	228	228	183	183	198	202	150	150	120	134	160	166	195	207	174	194	255	259	214	216	139	145	165	167
PQSS19	228	228	183	185	214	214	144	156	134	136	158	164	193	199	190	192	255	259	214	216	139	145	165	167
PQSS20	228	228	175	185	176	208	142	174	130	130	164	166	189	193	190	196	255	255	216	216	135	145	167	167
PQSS21	226	228	175	185	182	196	144	170	130	130	158	166	189	193	190	202	255	255	214	216	139	145	165	167
PQSS22	228	228	183	185	208	212	146	164	120	134	160	166	189	193	190	200	255	255	214	214	141	145	165	165
PQSS23	222	228	183	183	212	212	142	152	130	130	150	158	189	191	194	196	255	261	214	214	141	145	165	165
PQSS24	228	228	183	183	192	214	146	158	120	130	154	164	189	191	190	194	255	273	214	216	141	141	165	167
PQSS25	228	228	181	181	212	214	142	142	120	136	152	158	189	191	160	190	255	271	214	216	141	145	165	167
PQSS26	226	228	181	187	176	212	142	142	120	136	154	164	187	189	202	206	255	261	216	216	139	141	167	167
PQSS27	228	228	179	179	188	188	142	160	130	134	156	160	189	191	190	192	255	261	214	214	139	145	165	165
PQSS28	228	228	181	181	212	212	144	156	130	134	152	160	191	195	192	200	255	273	214	214	141	145	165	165
PQSS29	228	228	183	183	192	210	142	156	130	134	166	168	191	195	196	208	255	255	214	214	141	145	165	165
PQSS30	228	228	181	185	192	206	142	148	128	128	152	160	191	193	196	206	255	255	214	214	139	145	165	165
PQSS31	226	228	183	183	210	212	144	154	128	132	160	160	187	191	190	194	257	261	214	216	139	145	165	167
PQSS32	226	228	181	183	192	192	146	164	132	132	152	158	189	193	192	202	257	275	212	214	139	145	163	165
PQSS33	226	228	185	185	194	194	156	156	118	134	162	168	185	191	192	206	257	257	214	214	139	145	165	165
PQSS34	226	228	183	183	170	190	144	156	120	132	164	166	189	191	190	192	257	257	214	214	141	145	165	165
PQSS35	226	228	183	183	204	204	144	156	130	130	164	166	189	189	190	208	257	257	214	214	139	145	165	165
PQSS36	226	228	183	183	194	202	142	156	130	130	158	168	189	191	192	194	255	255	214	214	141	145	165	165
PQSS37	226	228	183	183	196	206	156	156	120	128	156	166	185	191	192	194	255	255	212	214	141	145	163	165
PQSS38	226	228	183	183	196	200	142	156	130	130	158	166	189	189	192	196	255	255	212	214	141	145	163	165
PQSS39	226	228	169	183	196	196	144	154	130	130	156	166	185	189	174	208	255	255	212	214	141	145	163	165
PQSS40	226	228	183	183	186	208	144	156	130	130	156	166	185	189	190	202	255	273	212	214	139	145	163	165
PQSS41	228	228	183	183	192	210	142	156	120	134	162	168	187	191	192	194	255	255	190	214	139	145	165	165
PQSS42	228	228	185	187	196	212	154	154	130	130	158	166	189	191	192	202	255	255	216	216	139	145	167	167
PQSS43	228	228	183	187	204	216	142	156	130	130	186	186	201	201	192	200	255	255	214	214	139	145	165	165
PQSS44	228	228	183	183	196	200	144	158	130	130	164	170	187	191	192	200	255	271	216	216	139	145	167	167
PQSS45	228	228	183	183	196	200	144	158	130	130	168	168	187	191	192	198	255	255	214	216	139	145	165	167
PQSS46	228	228	175	183	200	206	142	172	130	130	168	168	185	189	188	196	255	255	214	214	139	145	165	165
PQSS47	228	228	183	183	214	214	142	140	130	130	168	186	185	189	192	198	255	261	214	214	141	145	165	165
PQSS48	228	228	175	175	194	202	146	146	130	130	168	186	191	191	192	196	255	261	212	214	141	145	163	165
PQSS49	228	228	183	183	186	192	144	158	130	130	164	168	187	191	190	196	255	261	214	214	139	145	165	165
PQSS50	228	228	175	175	204	214	154	156	130	130	160	166	193	197	194	196	255	261	212	214	139	145	163	165
PQLP01	226	228	181	183	208	208	160	160	122	122	188	192	191	195	196	196	255	259	212	214	139	145	165	167
PQLP02	228	234	183	183	198	210	176	178	130	136	188	188	189	191	190	202	255	275	212	212	141	141	165	165
PQLP03	228	228	183	183	192	208	174	188	138	138	188	192	191	197	186	196	255	273	212	214	141	141	165	167
PQLP04	226	230	179	181	174	192	160	170	130	130	188	192	189	191	188	198	253	257	212	212	141	145	165	165
PQLP05	228	228	171	183	198	210	160	160	132	132	178	192	191	193	186	188	255	257	214	214	143	145	167	167
PQLP06	228	228	173	183	208	210	162	162	136	140	188	188	191	191	190	192	255	271	212	212	139	145	165	165
PQLP07	228	228	171	183	206	214	160	160	138	138	186	190	171	193	186	192	255	255	214	214	141	145	167	167
PQLP08	226	230	173	183	198	210	176	176	140	140	180	180	191	193	186	188	255	259	212	214	139	145	165	167
PQLP09	228	228	171	183	192	210	162	178	134	134	188	188	191	193	186	192	255	261	212	214	141	145	165	167
PQLP10	228	228	183	183	208	212	162	178	134	138	188	188	191	191	188	188	253	257	214	214	139	145	167	167
PQLP11	228	228	181	187	208	208	164	164	134	134	188	188	191	193	188	188	255	259	212	212	141	147	165	165
PQLP12	228	230	181	189	208	212	176	176	130	130	188	188	191	193	196	204	255	259	214	214	139	145	167	167
PQLP13	228	234	173	183	194	204	162	162	120	120	188	188	189	191	186	192	255	257	214	214	141	145	167	167
PQLP14	228	228	171	183	210	210	166	176	130	134	188	192	189	191	188	190	257	257	212	212	141	145	165	165
PQLP15	228	244	183	185	186	188	162	162	130	134	176	188	191	193	188	188	255	269	212	214	141	145	165	167
PQLP16	226	228	183	187	194	210	172	176	128	128	188	188	189	191	186	188	257	273	212	212	139	145	165	165
PQLP17	226	228	181	185	206	210	162	164	126	132	184	186	189	191	190	198	255	273	212	214	143	145	165	167
PQLP18	212	228	185	191	208	208	164	176	126	132	184	188	199	201	186	188	257	263	214	214	143	145	167	167
PQLP19	226	230	181	185	196	196	174	178	130	130	188	188	191	193	186	196	255	273	212	212	139	145	165	165
PQLP20	228	234	181	185	198	212	174	178	130	130	188	188	191	193	186	196	253	275	212	214	141	145	165	167
PQLP21	228	228	183	183	198	214	176	176	130	132	188	188	189	191	192	192	255	257	214	216	141	145	167	169
PQLP22	226	228	183	185	198	212	162	162	130	130	188	188	189	191	186	188	253	261	0	0	143	147	0	0
PQLP23	200	214	183	191	198	212	162	164	130	130	186	188	189	191	186	190	255	259	214	214	143	145	167	167
PQLP24	226	228	181	185	202	206	162	162	136	136	186	188	191	193	186	196	253	257	214	214	141	145	167	167
PQLP25	228	246	181	185	192	192	160	160	120	136	184	188	191	193	192	196	253	273	214	214	141	145	167	167
PQLP26	228	230	183	183	208	208	160	188	120	136	188	188	191	193	186	196	257	273	214	214	141	145	167	167
PQLP27	228	228	183	185	184	210	164	178	134	134	188	188	193	193	188	204	255	259	212	212	139	145	165	165
PQLP28	230	230	183	185	208	210	176	176	130	130	188	188	191	193	158	188	255	255	212	214	141	145	165	167
PQLP29	226	228	183	185	210	212	162	178	134	134	188	188	191	191	186	188	255	255	212	212	143	145	165	165
PQLP30	228	228	181	185	208	208	162	162	134	134	184	188	189	191	196	202	257	257	214	214	139	145	167	167
PQLP31	222	228	169	183	206	210	166	166	136	136	186	192	189	193	186	188	257	257	212	214	137	145	165	167
PQLP32	228	234	169	183	204	204	172	176	138	140	188	190	193	195	186	188	257	273	212	214	139	145	165	167
PQLP33	228	244	179	185	210	210	158	176	138	138	188	188	191	193	190	192	255	273	212	212	141	145	165	165
PQLP34	228	228	169	183	210	210	160	184	134	134	188	188	189	191	194	204	255	273	212	214	141	145	165	167
PQLP35	228	230	169	183	210	212	160	184	138	138	188	188	189	193	194	204	255	259	212	214	139	145	165	167
PQLP36	228	228	185	185	210	210	162	178	132	136	188	188	191	191	190	202	255	259	212	216	143	145	165	169
PQLP37	228	228	183	183	208	212	160	160	130	130	186	188	193	193	202	202	247	255	212	214	139	145	165	167
PQLP38	228	228	185	185	206	208	160	186	130	130	188	188	189	191	192	204	247	257	212	212	143	145	165	165
PQLP39	226	228	171	183	206	210	162	162	134	134	186	190	189	191	186	190	255	271	212	214	141	145	165	167
PQLP40	226	228	183	185	210	210	160	176	132	132	188	188	191	191	186	188	247	259	214	214	143	145	167	167
PQLP41	228	228	173	183	206	212	160	176	138	138	188	190	191	191	186	188	247	259	0	0	143	145	0	0
PQLP42	230	240	183	187	192	204	162	162	130	138	186	188	189	193	186	188	255	259	214	214	139	145	167	167
PQLP43	226	230	183	187	210	212	160	176	134	134	188	188	189	193	196	202	255	273	212	214	143	145	165	167
PQLP44	228	228	183	187	192	202	160	178	128	132	188	192	191	193	196	196	249	259	212	212	141	145	165	165
PQLP45	226	230	183	187	208	210	160	162	128	132	190	190	191	193	192	194	255	273	212	214	139	145	165	167
PQLP46	228	228	185	189	206	210	160	160	128	134	190	190	189	193	186	188	255	275	212	214	145	145	165	167
PQLP47	216	228	185	187	206	206	162	162	130	130	190	192	191	191	204	204	255	255	214	214	139	145	167	167
PQLP48	228	230	185	187	210	210	164	170	130	130	190	190	191	191	196	196	255	255	214	214	145	145	167	167
PQLP49	228	230	179	185	208	208	164	170	128	128	188	188	191	191	196	196	255	255	214	214	139	145	167	167
PQLP50	228	228	183	183	208	208	160	174	130	130	188	188	189	191	186	196	249	259	214	214	143	147	167	167
ONML01	228	228	183	189	196	200	166	172	130	130	166	168	195	197	188	190	253	261	214	214	139	145	167	167
ONML02	220	228	175	185	186	188	170	172	130	132	164	168	193	193	190	200	253	261	214	216	141	141	167	169
ONML03	228	228	183	185	210	212	170	172	132	142	166	172	195	197	190	198	251	261	214	214	143	143	167	167
ONML04	228	228	183	183	196	198	182	190	134	134	168	172	201	203	190	198	255	261	214	216	143	147	167	169
ONML05	228	228	181	183	180	206	172	188	130	132	168	170	189	191	192	198	255	261	214	214	141	145	167	167
ONML06	192	228	185	187	202	210	166	188	130	134	166	170	193	195	188	194	261	261	216	216	141	145	169	169
ONML07	228	228	173	187	202	206	170	184	134	134	166	170	191	195	190	202	253	261	214	214	143	143	167	167
ONML08	228	228	173	185	200	206	170	196	132	132	168	170	191	193	190	192	253	263	214	214	143	147	167	167
ONML09	194	228	185	187	190	212	166	186	128	132	168	186	189	191	192	198	253	261	214	216	139	143	167	169
ONML10	194	228	183	185	208	210	166	184	132	132	172	186	189	191	160	198	253	261	214	214	141	143	167	167
ONML11	232	232	183	185	190	214	166	184	132	132	168	172	193	195	160	190	253	263	214	214	141	143	167	167
ONML12	228	228	183	185	172	182	166	188	134	134	172	190	193	195	188	198	253	261	214	214	143	143	167	167
ONML13	228	228	173	185	190	192	166	184	134	134	170	174	189	193	190	190	253	261	214	216	143	145	167	169
ONML14	228	228	181	185	192	200	166	196	130	130	172	190	189	195	188	208	253	261	214	216	141	145	167	169
ONML15	234	234	185	191	190	220	166	184	132	132	172	178	193	193	192	194	251	263	214	214	143	145	167	167
ONML16	228	234	183	185	180	208	168	180	134	134	168	174	191	195	190	206	253	273	214	216	143	147	167	169
ONML17	228	228	185	187	208	210	166	188	130	134	170	180	191	195	186	190	253	261	214	214	143	147	167	167
ONML18	226	228	185	187	208	210	174	174	134	138	168	172	193	195	190	194	255	257	214	216	141	147	167	169
ONML19	226	226	183	187	200	204	168	178	132	132	166	172	193	195	190	198	253	261	214	214	143	147	167	167
ONML20	228	228	173	185	194	204	166	172	136	136	170	174	189	189	188	190	251	261	214	214	141	143	167	167
ONML21	228	228	183	185	210	212	170	178	136	140	168	172	195	195	188	192	261	261	214	216	141	143	167	169
ONML22	228	228	183	185	186	224	168	174	136	136	168	170	191	193	190	194	247	263	214	216	143	143	167	169
ONML23	228	228	173	173	190	210	170	172	130	130	170	174	191	193	190	194	259	259	214	214	143	145	167	167
ONML24	226	228	183	185	216	216	168	196	130	140	166	184	193	199	190	198	259	259	214	214	141	147	167	167
ONML25	228	228	183	185	204	206	170	184	130	130	168	170	201	203	192	194	259	271	212	216	143	145	165	169
ONML26	228	228	181	181	190	202	166	166	130	132	168	170	201	203	188	192	259	259	216	216	143	145	169	169
ONML27	228	228	187	187	192	208	166	166	130	132	166	170	201	203	192	198	257	271	214	214	143	147	167	167
ONML28	228	228	185	189	208	210	174	182	130	130	164	170	201	203	190	190	255	257	214	214	141	147	167	167
ONML29	234	234	183	187	202	210	166	200	130	132	168	172	191	193	190	190	255	257	214	214	143	145	167	167

ONML30	228	228	181	185	206	210	166	170	130	130	166	172	189	193	194	202	257	273	214	216	139	145	167	169
ONML31	228	228	185	187	206	210	166	170	132	136	166	170	191	193	188	198	259	271	214	216	141	143	167	169
ONML32	228	228	185	187	202	206	168	172	128	132	168	170	193	193	188	192	259	271	214	214	141	145	167	167
ONML33	228	228	171	185	188	206	164	170	132	134	168	170	189	191	188	192	255	273	214	214	143	143	167	167
ONML34	228	228	183	185	216	216	164	170	130	132	166	174	189	193	200	202	259	271	214	214	143	145	167	167
ONML35	228	228	181	187	204	218	164	200	132	134	166	172	187	191	190	194	259	271	216	216	141	145	169	169
ONML36	226	228	173	185	190	208	164	170	132	136	166	170	189	191	190	198	253	257	214	216	143	145	167	169
ONML37	228	228	181	183	186	186	164	168	130	132	168	172	187	189	188	190	257	271	216	216	143	145	169	169
ONML38	228	228	173	185	188	196	164	182	132	132	168	172	189	189	186	192	251	259	214	216	143	143	167	169
ONML39	226	228	183	185	174	190	168	174	134	134	166	172	187	189	186	190	251	259	214	214	141	143	167	167
ONML40	228	228	173	185	188	210	164	168	132	134	168	172	181	191	194	198	253	261	216	216	143	145	169	169
ONML41	228	228	183	191	206	212	164	200	132	132	166	172	181	191	194	198	253	261	216	216	143	147	169	169
ONML42	228	228	183	185	190	190	170	174	130	132	170	174	189	193	188	190	253	261	214	214	139	145	167	167
ONML43	228	228	185	189	190	208	164	198	132	132	170	192	181	191	200	202	259	259	214	214	143	147	167	167
ONML44	228	228	183	187	218	218	164	180	130	132	170	184	179	193	200	202	253	259	214	214	143	147	167	167
ONML45	228	228	173	185	190	200	166	166	132	132	168	172	189	191	190	198	253	271	214	214	143	147	167	167
ONML46	228	228	183	187	210	210	164	164	126	130	168	172	193	197	198	198	269	269	216	218	143	145	169	171
ONML47	228	228	173	185	182	206	166	184	132	132	166	172	179	195	192	192	271	271	214	214	143	145	167	167
ONML48	228	228	183	185	206	206	168	184	132	132	164	170	183	193	192	192	253	271	214	214	145	145	167	167
ONML49	228	228	175	187	206	210	166	184	132	132	166	170	191	191	190	208	253	257	214	214	141	147	167	167
ONML50	228	228	185	189	210	210	174	182	132	134	164	170	191	193	198	204	259	259	214	216	143	147	167	169
ONFR01	228	230	183	185	188	208	174	186	134	138	168	172	195	197	188	190	243	259	214	214	141	145	167	167
ONFR02	226	230	183	185	180	206	180	180	140	140	168	168	191	195	192	206	243	259	214	214	141	141	167	167
ONFR03	226	228	171	177	180	208	168	168	124	136	170	172	191	191	190	196	243	259	214	214	143	143	167	167
ONFR04	226	230	171	185	204	206	170	182	134	138	168	172	191	191	188	196	243	261	214	214	143	147	167	167
ONFR05	228	230	179	183	170	208	174	178	136	138	166	166	191	191	188	190	245	261	214	214	141	145	167	167
ONFR06	226	228	183	185	176	206	162	182	136	138	166	166	191	191	184	186	243	261	216	216	141	145	169	169
ONFR07	228	228	181	185	206	206	190	192	138	140	168	168	189	193	184	186	243	259	216	216	143	143	169	169
ONFR08	226	228	183	185	176	210	178	186	134	138	168	168	191	191	170	184	243	261	216	216	143	143	169	169
ONFR09	226	228	185	189	188	220	190	192	138	138	168	168	191	193	186	206	243	261	214	214	139	143	167	167
ONFR10	226	230	183	187	180	210	176	186	138	140	168	168	191	193	190	196	243	261	214	214	141	143	167	167
ONFR11	226	228	183	187	198	200	168	180	120	138	168	168	193	193	196	202	243	261	214	214	141	143	167	167
ONFR12	226	230	185	189	170	172	174	176	138	140	168	168	193	193	184	196	245	261	0	0	143	143	0	0
ONFR13	226	228	171	189	188	210	174	190	138	142	168	168	193	193	196	198	245	263	212	216	143	147	165	169
ONFR14	228	230	183	185	206	208	184	184	142	144	170	170	191	191	186	196	245	261	214	214	141	145	167	167
ONFR15	226	230	183	187	188	210	170	184	138	144	168	168	191	191	186	196	247	261	214	216	143	145	167	169
ONFR16	226	228	183	185	188	188	166	174	130	134	166	166	187	191	188	204	245	261	214	216	145	147	167	169
ONFR17	222	228	181	185	186	206	176	192	136	140	168	168	189	191	186	206	247	259	214	214	143	143	167	167
ONFR18	228	232	185	189	198	202	170	170	136	140	168	172	191	191	184	188	251	259	214	216	141	143	167	169
ONFR19	214	228	177	181	188	188	184	190	140	140	170	170	191	195	188	192	251	261	214	216	143	145	167	169
ONFR20	226	230	183	185	206	210	174	174	138	142	170	174	193	205	186	192	243	263	216	216	141	147	169	169
ONFR21	224	228	171	183	188	206	174	188	136	140	168	168	195	195	170	192	249	261	214	214	141	143	167	167
ONFR22	226	230	183	187	180	202	142	140	140	142	170	170	193	207	194	206	245	263	214	216	143	143	167	169
ONFR23	226	228	181	185	188	204	174	188	138	140	164	164	187	191	186	190	249	259	214	214	143	145	167	167
ONFR24	222	230	177	181	188	210	186	190	134	136	166	166	189	193	188	190	249	259	214	216	141	145	167	169
ONFR25	226	230	185	189	202	210	174	184	140	142	168	170	195	201	188	200	251	257	214	214	143	145	167	167
ONFR26	222	228	177	183	188	206	172	180	136	142	170	172	193	199	186	198	253	257	214	214	143	147	167	167
ONFR27	226	230	181	183	188	226	188	188	138	142	168	172	195	197	186	186	251	259	214	214	143	143	167	167
ONFR28	228	230	183	185	208	216	174	192	136	138	168	170	193	197	186	204	249	259	214	214	141	143	167	167
ONFR29	228	228	183	185	208	216	172	176	134	134	168	172	195	197	186	186	247	263	214	216	143	147	167	169
ONFR30	228	234	183	185	208	216	166	166	136	138	170	172	195	197	190	196	245	245	214	216	139	145	167	169
ONFR31	228	232	185	187	188	208	170	180	120	142	170	174	195	195	182	188	243	243	214	216	141	143	167	169
ONFR32	226	230	183	185	206	226	176	184	140	142	168	170	197	203	184	192	243	263	214	214	141	147	167	167
ONFR33	226	230	185	189	194	208	186	190	142	142	168	168	191	193	186	198	243	269	214	214	141	143	167	167
ONFR34	226	230	183	185	202	204	170	176	138	140	168	168	195	203	186	194	247	261	214	214	141	143	167	167
ONFR35	226	230	183	185	188	210	180	180	136	140	168	172	191	195	186	196	247	261	214	216	143	145	167	169
ONFR36	222	228	183	185	184	186	172	190	142	142	170	170	191	195	184	188	245	263	214	216	143	147	167	169
ONFR37	226	228	183	185	180	210	168	178	136	140	168	174	191	193	188	188	245	245	214	214	139	145	167	167
ONFR38	228	232	183	185	204	204	172	190	136	136	168	170	193	193	184	190	249	261	214	214	143	145	167	167
ONFR39	228	234	183	185	204	206	168	168	136	138	166	170	191	191	186	196	249	261	214	214	141	145	167	167
ONFR40	226	228	185	187	204	218	166	168	138	142	168	170	189	189	170	186	255	259	214	214	143	147	167	167
ONFR41	226	228	171	187	180	180	168	176	110	138	168	168	189	191	184	188	253	257	214	216	143	147	167	169
ONFR42	228	232	183	187	188	188	168	174	134	136	166	168	189	193	196	206	253	257	214	214	139	145	167	167
ONFR43	226	230	183	187	208	210	168	174	138	140	166	168	191	193	190	190	251	255	214	214	143	147	167	167
ONFR44	228	230	183	185	188	194	170	174	138	138	164	170	193	205	186	188	245	245	214	216	143	147	167	169
ONFR45	228	230	183	185	208	208	192	198	136	140	170	170	193	195	172	186	245	263	214	214	143	147	167	167
ONFR46	228	230	171	185	204	206	190	190	144	146	168	168	191	195	184	186	245	263	214	214	143	145	167	167
ONFR47	226	228	187	191	188	188	174	190	140	144	170	170	191	195	172	188	245	261	214	214	141	145	167	167
ONFR48	228	230	183	185	214	216	142	180	138	142	168	168	191	193	186	196	247	261	214	216	145	145	167	169
ONFR49	226	230	183	187	188	206	174	192	140	142	166	166	189	193	186	196	247	261	214	214	141	147	167	167
ONFR50	222	228	183	185	204	206	180	194	142	146	166	170	187	191	188	196	247	261	214	214	143	147	167	167
ONHF01	228	228	185	187	198	208	162	176	134	136	180	180	195	195	188	192	255	269	214	214	139	145	167	167
ONHF02	228	236	183	183	208	212	148	160	134	136	176	176	191	195	188	190	255	255	214	216	141	143	167	169
ONHF03	226	230	171	171	208	212	148	160	132	134	176	176	193	201	192	192	255	255	214	216	143	143	167	169
ONHF04	230	236	189	207	192	210	148	160	134	134	170	178	181	191	188	188	249	255	214	214	143	147	167	167
ONHF05	228	228	171	185	210	212	148	158	132	134	142	166	177	191	190	200	255	269	212	212	143	145	165	165
ONHF06	230	230	189	189	208	212	148	158	130	134	166	166	179	191	196	196	253	253	214	214	141	143	167	167
ONHF07	230	230	185	209	208	208	150	178	130	132	154	166	185	185	192	200	255	271	214	214	143	145	167	167
ONHF08	230	230	185	187	184	190	158	160	130	132	164	168	179	189	192	206	249	257	214	216	143	145	167	169
ONHF09	228	230	185	185	206	210	158	172	128	130	164	164	189	191	190	190	253	253	0	0	143	143	0	0
ONHF10	230	230	185	207	206	206	146	160	126	128	164	166	181	191	190	190	255	255	214	214	141	145	167	167
ONHF11	230	230	185	205	206	210	160	174	132	132	164	166	187	187	194	200	255	255	214	216	143	147	167	169
ONHF12	230	230	185	187	200	210	148	160	132	134	166	166	189	191	190	190	253	253	214	214	143	145	167	167
ONHF13	230	230	185	185	190	208	148	158	128	128	166	166	191	191	158	190	253	253	214	214	143	143	167	167
ONHF14	230	230	185	205	200	210	148	160	130	134	164	166	187	191	190	190	255	255	214	214	141	145	167	167
ONHF15	228	230	187	205	208	210	148	160	128	130	160	170	181	191	194	204	259	271	214	214	143	143	167	167
ONHF16	222	228	169	183	188	208	150	158	128	128	166	166	187	191	190	198	255	255	214	214	141	143	167	167
ONHF17	222	228	169	183	206	206	150	160	134	136	164	166	181	189	192	194	253	253	214	214	143	143	167	167
ONHF18	226	228	169	183	180	208	152	160	130	134	166	168	201	201	192	194	249	257	216	216	143	143	169	169
ONHF19	228	228	183	191	174	208	158	160	130	132	164	168	181	189	192	196	255	255	214	214	141	143	167	167
ONHF20	228	228	183	203	170	186	148	158	130	132	166	166	189	189	190	190	253	269	214	216	141	143	167	169
ONHF21	228	228	185	203	206	206	160	172	132	136	156	166	189	189	192	206	253	271	214	214	141	143	167	167
ONHF22	228	228	201	203	206	206	160	178	130	132	156	166	189	189	184	190	249	257	216	216	143	143	169	169
ONHF23	222	228	181	181	186	196	160	170	130	132	154	166	183	191	192	198	253	269	216	216	143	145	169	169
ONHF24	228	228	185	201	206	206	158	172	130	132	166	166	179	189	194	196	253	269	214	216	141	145	167	169
ONHF25	226	228	181	199	196	206	146	162	130	132	166	170	191	191	198	202	249	255	214	216	143	145	167	169
ONHF26	228	230	181	199	188	188	156	166	130	136	166	172	191	191	210	210	253	253	214	214	143	145	167	167
ONHF27	228	228	181	181	200	206	150	164	134	134	166	166	191	191	194	208	253	273	214	214	143	143	167	167
ONHF28	228	228	171	183	176	208	150	158	134	136	166	166	191	191	192	196	253	255	214	214	143	143	167	167
ONHF29	228	228	181	183	202	204	144	170	132	132	166	166	191	193	190	192	253	255	214	214	143	145	167	167
ONHF30	226	228	183	199	202	204	148	160	130	132	166	166	193	193	192	192	249	257	214	214	139	145	167	167
ONHF31	226	228	185	199	190	208	146	158	130	130	164	166	191	191	190	206	251	257	214	216	141	143	167	169
ONHF32	228	228	185	199	206	206	154	182	130	130	164	166	181	193	198	200	255	255	214	216	141	145	167	169
ONHF33	228	228	183	201	190	210	158	174	128	130	166	166	189	199	190	194	255	255	214	216	143	147	167	169
ONHF34	226	228	183	185	184	210	160	174	128	130	142	166	179	189	192	198	251	257	214	216	141	143	167	169
ONHF35	228	228	183	183	210	210	166	166	134	140	142	166	187	191	192	198	253	253	214	214	139	145	167	167
ONHF36	228	228	183	199	202	206	158	166	128	130	142	166	191	191	192	194	255	255	214	216	143	147	167	169
ONHF37	226	228	183	201	202	206	146	158	126	128	166	166	189	191	196	200	255	255	214	214	139	145	167	167
ONHF38	228	228	183	201	190	208	158	172	128	128	166	166	189	189	194	202	255	255	214	214	143	145	167	167
ONHF39	226	228	185	201	178	208	158	178	126	128	168	184	189	189	194	198	257	257	214	214	141	145	167	167
ONHF40	226	230	185	201	206	206	146	156	132	134	142	164	187	189	190	194	255	255	214	214	143	147	167	167
ONHF41	228	230	183	183	190	210	148	158	132	134	166	166	179	189	190	210	253	257	214	216	143	147	167	169
ONHF42	228	228	185	185	188	216	146	158	132	134	166	166	189	193	194	198	255	271	214	214	139	145	167	167
ONHF43	224	230	183	185	218	218	146	158	132	132	166	166	189	189	202	206	255	271	214	214	143	147	167	167
ONHF44	230	230	183	201	190	210	158	172	130	132	166	166	191	191	190	202	251	257	216	216	143	147	169	169
ONHF45	190	230	201	203	210	210	146	158	130	132	166	166	187	191	190	202	253	257	216	216	143	147	169	169
ONHF46	230	230	183	203	210	210	156	174	128	128	166	166	179	191	194	200	251	255	214	214	143	145	167	167
ONHF47	230	230	185	203	178	206	156	170	128	130	164	168	187	191	190	202	255	271	214	214	141	147	167	167
ONHF48	230	236	183	185	178	206	162	172	128	130	166	166	187	191	202	202	255	255	216	216	145	145	169	169
ONHF49	230	232	183	185	190	210	164	176	132	136	144	168	187	191	194	206	255	255	214	214	141	147	167	167
ONHF50	228	228	183	185	180	206	160	174	130	130	168	168	189	189	190	190	255	255	214	214	143	147	167	167
ONGR01	230	232	185	185	206	206	160	166	130	134	164	166	191	191	190	192	255	259	214	214	143	147	167	167
ONGR02	230	230	181	185	206	208	160	174	130	132	164	166	195	201	190	192	255	261	214	214	143	143	167	167

ONGR03	230	232	179	183	216	216	164	172	132	134	166	166	193	193	188	190	257	259	214	214	145	145	167	167
ONGR04	230	234	179	185	206	206	156	166	130	132	162	164	191	191	190	190	257	259	214	214	145	149	167	167
ONGR05	232	234	183	187	204	204	156	156	130	134	164	166	193	193	192	196	259	261	214	214	143	147	167	167
ONGR06	230	232	181	187	206	210	148	148	132	136	130	168	193	193	190	198	259	261	214	214	143	147	167	167
ONGR07	232	246	185	189	206	206	158	172	128	132	164	166	191	191	188	192	257	275	214	214	143	145	167	167
ONGR08	204	232	183	187	206	210	158	158	132	134	164	166	193	209	160	186	263	265	214	216	141	145	167	169
ONGR09	232	232	183	187	204	208	158	174	128	146	166	168	193	193	188	188	259	271	212	216	141	145	165	169
ONGR10	232	232	185	187	204	208	156	158	132	134	166	166	191	191	188	198	259	279	214	216	143	145	167	169
ONGR11	232	234	185	187	206	210	174	178	134	136	166	166	191	201	190	192	259	263	214	216	143	145	167	169
ONGR12	232	232	183	185	208	212	160	186	132	134	162	164	191	193	188	194	263	263	214	216	145	145	167	169
ONGR13	232	232	175	187	206	212	156	160	126	128	166	192	191	191	190	192	259	263	214	216	143	149	167	169
ONGR14	232	232	183	187	208	212	162	164	126	128	166	192	193	201	192	194	259	279	214	216	143	147	167	169
ONGR15	232	232	183	187	206	208	172	172	124	128	166	168	193	193	192	194	263	265	216	218	145	147	169	171
ONGR16	232	238	183	185	206	210	158	174	126	146	166	168	191	191	190	196	261	267	216	218	143	149	169	171
ONGR17	232	232	185	185	206	208	154	158	130	132	166	166	189	189	190	194	267	269	214	218	141	145	167	171
ONGR18	228	232	183	185	204	208	156	156	128	132	166	168	191	193	192	196	265	269	216	220	143	145	169	173
ONGR19	228	232	179	187	204	206	158	158	130	132	166	168	191	191	194	196	259	259	218	222	145	147	171	175
ONGR20	230	232	183	183	202	210	156	174	130	140	166	166	193	193	188	192	261	277	214	218	143	149	167	171
ONGR21	230	230	183	187	204	208	156	174	128	128	166	166	191	195	188	192	263	265	214	218	143	145	167	171
ONGR22	228	230	187	187	204	208	158	174	128	132	166	166	191	193	186	188	261	267	214	220	145	145	167	173
ONGR23	228	230	183	183	210	212	158	158	130	132	166	166	189	189	190	192	263	267	214	218	145	147	167	171
ONGR24	230	230	183	187	206	210	164	176	126	130	164	166	191	191	188	192	265	269	216	218	143	147	169	171
ONGR25	230	230	181	187	194	210	154	158	130	132	166	166	189	193	190	194	265	267	214	216	145	147	167	169
ONGR26	228	230	185	187	208	214	158	162	130	132	166	166	195	195	188	190	257	263	214	216	145	149	167	169
ONGR27	230	230	185	193	204	210	156	158	122	130	166	168	193	201	188	196	259	265	214	216	145	145	167	169
ONGR28	230	230	185	191	206	206	178	180	130	130	168	168	193	193	190	192	259	263	214	218	143	145	167	171
ONGR29	232	236	185	187	208	208	162	168	130	134	166	168	191	195	188	190	259	263	214	216	143	149	167	169
ONGR30	230	230	185	187	202	204	160	168	132	132	164	166	191	191	188	192	257	273	214	216	141	147	167	169
ONGR31	228	230	171	185	182	192	158	172	130	132	164	166	191	193	162	188	257	267	214	218	143	145	167	171
ONGR32	230	230	181	185	190	206	160	170	128	132	144	166	189	191	186	190	265	269	214	216	141	149	167	169
ONGR33	222	230	181	183	204	204	158	176	134	134	162	166	191	193	186	188	267	271	210	214	141	145	163	167
ONGR34	230	230	171	185	204	208	156	176	134	134	166	166	191	193	186	188	265	269	214	216	143	145	167	169
ONGR35	228	230	183	187	204	204	162	168	130	132	140	166	191	191	186	190	255	257	214	216	145	147	167	169
ONGR36	228	230	187	187	206	208	160	174	108	128	164	168	193	201	186	190	257	261	214	218	145	149	167	171
ONGR37	230	230	187	187	208	212	156	172	128	128	148	166	193	193	160	188	257	263	214	218	141	147	167	171
ONGR38	230	230	181	183	196	210	156	172	130	146	144	166	191	195	180	186	255	259	214	216	145	147	167	169
ONGR39	228	230	171	183	196	210	158	174	128	132	166	166	191	193	186	190	259	265	214	216	143	147	167	169
ONGR40	228	230	183	185	188	210	170	170	130	130	164	166	193	195	186	192	255	265	212	214	143	149	165	167
ONGR41	230	232	181	185	210	210	158	172	130	130	164	166	191	193	186	192	261	265	214	216	143	149	167	169
ONGR42	230	230	179	187	206	210	160	164	130	134	168	168	191	199	186	190	257	263	214	216	141	147	167	169
ONGR43	230	232	187	187	210	210	162	164	134	134	164	166	191	199	188	188	257	265	214	218	145	149	167	171
ONGR44	226	232	183	185	210	214	156	156	134	134	166	168	191	191	186	196	259	265	214	214	145	149	167	167
ONGR45	232	232	185	187	208	214	158	174	130	134	166	166	193	193	160	188	259	265	214	216	145	149	167	169
ONGR46	220	232	187	189	206	210	160	174	128	130	166	166	191	193	186	192	257	267	212	214	145	147	165	167
ONGR47	232	232	185	187	206	210	158	158	128	130	166	166	191	201	184	192	253	253	212	214	143	147	165	167
ONGR48	230	230	185	185	188	210	160	172	130	136	166	168	191	191	160	188	253	253	212	214	147	147	165	167
ONGR49	226	230	183	185	204	206	156	176	128	130	166	166	191	191	192	196	253	253	212	214	143	149	165	167
ONGR50	232	232	181	183	190	208	158	172	128	128	166	166	191	191	190	192	253	253	212	218	145	145	165	171
ONMWL01	228	228	183	183	208	212	180	184	130	146	166	166	163	191	190	190	255	271	214	216	145	149	167	169
ONMWL02	222	228	181	183	208	212	150	160	136	148	164	166	191	191	188	198	255	271	214	216	143	147	167	169
ONMWL03	200	200	183	183	204	210	158	160	148	148	164	164	191	193	188	198	255	255	214	214	143	147	167	167
ONMWL04	200	228	171	183	210	212	164	174	130	150	164	164	191	191	188	192	255	271	212	216	145	145	165	169
ONMWL05	226	228	183	183	210	212	160	160	132	150	150	164	191	193	188	196	255	255	214	214	145	145	167	167
ONMWL06	204	228	183	183	208	224	148	160	128	130	144	164	191	193	192	198	255	255	214	216	139	145	167	169
ONMWL07	228	242	183	183	210	212	158	174	132	148	162	168	193	199	192	198	255	273	214	214	143	145	167	167
ONMWL08	200	228	181	183	206	208	162	174	160	168	160	166	191	191	198	200	255	255	214	214	139	145	167	167
ONMWL09	228	228	185	187	206	212	158	160	128	148	152	166	191	193	188	190	255	255	214	216	143	143	167	169
ONMWL10	228	228	183	183	210	212	158	160	130	144	162	164	193	193	190	192	255	255	212	216	139	145	165	169
ONMWL11	228	230	183	185	206	208	158	160	144	154	164	166	193	195	188	198	255	273	214	216	143	147	167	169
ONMWL12	228	228	181	183	210	210	160	160	128	130	162	166	193	193	188	190	255	255	214	214	139	145	167	167
ONMWL13	228	228	183	185	206	206	160	160	128	146	162	166	193	193	194	194	255	255	214	214	143	143	167	167
ONMWL14	228	228	183	183	210	210	160	160	130	146	162	164	193	193	192	198	255	255	214	216	141	145	167	169
ONMWL15	212	212	183	185	192	208	150	160	130	146	162	164	191	195	190	206	255	273	214	216	145	149	167	169
ONMWL16	228	234	183	185	204	208	162	168	130	146	164	166	191	191	200	204	255	255	214	216	143	147	167	169
ONMWL17	228	228	183	183	206	208	158	158	132	146	144	166	191	193	198	206	255	255	214	216	145	147	167	169
ONMWL18	224	228	183	185	172	190	158	174	146	148	144	164	193	193	202	206	255	255	214	214	143	143	167	167
ONMWL19	224	228	179	183	194	194	174	174	116	148	144	164	191	191	194	198	253	271	214	214	139	143	167	167
ONMWL20	226	228	169	179	212	216	150	158	128	132	166	166	193	193	188	188	255	271	214	216	141	143	167	169
ONMWL21	214	226	183	183	190	212	158	158	136	146	166	166	193	193	206	206	255	271	214	214	141	143	167	167
ONMWL22	204	204	183	183	194	204	148	158	136	140	144	166	191	191	194	200	255	255	214	216	143	143	167	169
ONMWL23	190	190	183	183	198	206	158	172	138	148	166	166	193	193	198	206	255	255	214	216	143	145	167	167
ONMWL24	212	226	181	183	190	206	160	174	134	144	164	168	191	193	174	188	255	255	214	214	141	145	167	167
ONMWL25	226	226	181	181	204	206	152	160	132	136	166	166	191	193	190	206	255	271	214	218	143	145	167	171
ONMWL26	212	226	181	181	182	216	148	158	134	144	168	170	191	193	190	200	255	255	214	214	143	145	167	167
ONMWL27	200	226	181	181	214	216	148	160	136	146	168	168	191	195	190	198	255	255	214	216	143	143	167	169
ONMWL28	226	226	183	183	190	208	148	160	130	132	168	168	191	191	198	198	255	255	214	216	141	143	167	169
ONMWL29	228	232	179	181	208	210	158	160	132	132	168	170	191	201	190	190	253	253	214	214	143	145	167	167
ONMWL30	226	226	169	179	188	206	150	158	128	134	168	168	191	191	190	206	253	253	214	214	139	145	167	167
ONMWL31	200	226	181	181	208	210	150	158	116	146	166	168	169	191	192	198	255	255	214	216	141	143	167	169
ONMWL32	226	226	183	189	188	206	148	158	128	148	132	140	189	191	190	200	255	255	214	214	143	145	167	167
ONMWL33	218	226	181	181	174	184	158	172	138	146	162	166	191	195	192	192	255	255	214	214	145	145	167	167
ONMWL34	226	226	181	181	208	208	146	158	130	146	152	168	191	193	190	190	255	255	216	216	145	147	169	169
ONMWL35	200	226	181	183	192	206	148	158	130	134	162	168	191	193	190	196	255	255	214	214	143	147	167	167
ONMWL36	224	226	183	185	208	210	156	158	144	146	152	168	191	193	202	202	255	255	216	216	145	147	169	169
ONMWL37	226	226	183	183	190	208	160	184	132	148	166	168	191	195	188	190	255	255	214	216	145	149	167	169
ONMWL38	226	226	183	183	208	218	148	174	128	146	162	166	189	191	194	196	255	255	214	214	145	145	167	167
ONMWL39	224	226	183	185	190	194	152	152	128	144	166	166	193	193	190	198	255	273	214	214	143	145	167	167
ONMWL40	200	226	169	191	190	204	160	178	134	134	158	166	189	191	190	198	255	271	214	214	145	149	167	167
ONMWL41	226	228	181	183	178	204	158	174	128	148	160	166	189	191	198	198	255	255	214	214	141	147	167	167
ONMWL42	226	226	183	183	206	208	160	162	130	150	156	166	191	191	190	192	255	255	214	214	143	145	167	167
ONMWL43	226	228	183	183	204	208	164	174	130	134	164	164	191	201	174	198	255	255	214	216	143	149	167	169
ONMWL44	222	228	183	183	192	218	158	174	132	132	164	166	201	201	190	208	255	255	214	214	143	145	167	167
ONMWL45	228	228	183	183	206	208	158	172	148	148	164	166	193	195	190	198	255	255	214	214	143	147	167	167
ONMWL46	216	228	183	183	190	194	148	158	132	148	152	166	193	193	192	192	255	255	214	216	143	145	167	169
ONMWL47	228	228	183	183	190	196	168	174	134	146	164	166	193	193	194	198	255	273	214	214	141	145	167	167
ONMWL48	226	226	183	185	208	208	158	172	128	138	166	166	195	195	188	188	255	255	214	216	145	145	167	169
ONMWL49	222	226	183	183	206	216	154	182	132	144	164	166	195	195	188	188	255	255	214	216	141	147	167	169
ONMWL50	228	228	181	183	200	200	150	158	130	130	162	168	193	193	190	192	255	271	214	216	143	147	167	169
ONRC01	226	226	185	185	202	206	166	166	126	128	168	170	191	191	192	192	255	255	212	216	139	145	167	171
ONRC02	226	226	181	185	202	206	168	172	134	134	166	168	191	191	192	192	257	259	210	214	141	141	165	169
ONRC03	224	228	187	191	192	206	164	170	134	134	168	168	191	191	192	192	255	259	212	214	143	143	167	169
ONRC04	226	228	183	185	192	208	166	168	132	134	166	168	191	191	192	204	255	259	210	214	143	147	165	169
ONRC05	226	228	187	199	204	204	166	170	130	130	166	166	191	191	194	194	257	257	212	214	143	145	167	169
ONRC06	226	226	183	185	188	204	166	168	128	128	164	164	191	191	194	194	255	255	210	214	139	145	165	169
ONRC07	226	226	183	201	208	208	172	172	130	132	164	166	191	191	192	192	255	261	212	214	143	145	167	169
ONRC08	224	226	187	191	204	208	168	170	132	136	166	166	191	193	192	194	257	257	214	214	139	145	169	169
ONRC09	226	228	181	185	206	212	166	172	130	136	166	166	191	191	192	192	257	257	214	218	143	143	169	169
ONRC10	226	226	181	187	204	208	164	172	130	132	166	166	189	189	192	192	257	261	214	218	139	145	169	173
ONRC11	226	226	181	183	204	206	168	168	132	132	166	184	191	191	190	194	257	263	212	218	143	147	167	169
ONRC12	212	226	181	183	202	206	164	172	122	122	164	168	191	191	194	194	257	263	212	216	139	145	167	171
ONRC13	226	226	183	187	200	206	168	170	130	130	168	168	193	193	192	194	257	261	214	216	143	143	169	169
ONRC14	226	226	185	191	202	202	168	172	130	130	168	168	189	189	192	192	259	261	214	218	141	145	169	173
ONRC15	220	226	181	181	190	214	170	170	130	134	168	168	189	189	190	194	257	265	214	216	141	145	169	171
ONRC16	228	230	183	183	172	182	168	172	130	134	168	170	189	189	192	192	257	263	214	218	141	145	169	173
ONRC17	226	230	183	189	204	208	168	170	132	134	168	168	189	189	192	192	259	259	212	216	143	143	167	167
ONRC18	220	228	183	185	208	208	168	172	128	132	166	166	191	191	192	192	259	263	208	214	143	143	163	169
ONRC19	220	220	183	185	204	212	168	172	128	132	168	170	189	189	192	192	259	259	212	216	139	143	167	167
ONRC20	228	228	181	181	208	210	168	172	130	134	168	168	191	191	168	194	259	263	210	216	141	143	165	171
ONRC21	228	228	183	189	200	204	166	170	130	132	168	168	189	189	192	192	259	263	212	214	141	143	167	169
ONRC22	228	228	185	187	208	208	168	170	128	132	158	168	189	189	192	192	259	259	212	216	143	143	167	171
ONRC23	222	228	183	183	204	208	168	172	126	128	168	186	177	177	180	192	261	263	210	216	143	145	165	171
ONRC24	228	232	183	189	200	200	170	170	128	128	166	168	191	191	194	194	259	261	214	216	141	145	169	171
ONRC25	226	228	183	183	210	210	170	172	128	128	166	170	191	191	192	192	259	261	210	216	143	145	165	171

ONRC26	228	228	183	183	208	208	168	168	128	128	166	168	191	191	164	164	259	265	212	212	143	145	167	167
ONRC27	230	230	181	181	186	208	166	172	130	130	166	170	183	183	184	184	259	261	212	216	143	143	167	167
ONRC28	230	230	185	187	204	210	166	172	130	132	168	168	191	191	194	194	259	261	214	218	141	143	169	169
ONRC29	230	230	195	197	210	210	168	172	132	142	168	168	191	191	194	194	257	259	210	216	143	145	165	171
ONRC30	228	230	185	197	194	206	168	172	136	136	168	168	191	191	192	192	259	259	212	216	139	145	167	171
ONRC31	230	230	185	189	190	190	168	172	132	134	166	170	189	189	192	192	257	259	212	216	141	143	167	171
ONRC32	228	228	185	189	190	206	166	170	132	134	168	194	189	189	192	194	257	261	212	216	141	145	167	169
ONRC33	226	230	185	191	206	210	168	172	130	142	168	168	189	189	192	194	257	261	212	216	141	143	167	169
ONRC34	230	230	183	183	206	206	168	168	134	134	166	170	191	191	188	194	257	261	214	218	141	143	169	173
ONRC35	230	230	183	183	172	190	170	172	128	134	168	168	189	199	190	202	259	261	212	216	137	145	167	171
ONRC36	230	232	183	183	206	210	170	170	130	130	166	168	169	169	188	192	257	261	210	216	143	143	165	169
ONRC37	226	230	183	187	184	200	172	172	138	138	166	170	165	165	190	192	259	259	212	214	139	145	167	169
ONRC38	226	230	185	187	190	208	170	170	130	130	168	168	189	189	190	192	259	259	214	214	143	145	169	169
ONRC39	228	228	185	185	210	210	170	170	122	126	168	168	167	191	190	194	259	259	216	216	141	145	171	171
ONRC40	230	230	183	183	202	202	168	172	116	116	166	170	187	187	190	194	259	261	212	216	143	143	167	171
ONRC41	230	230	183	187	184	202	168	172	128	128	166	170	187	187	190	194	259	261	212	216	143	147	167	171
ONRC42	228	238	183	185	184	184	168	172	130	130	168	168	187	189	190	194	257	261	214	216	139	145	169	171
ONRC43	228	228	181	181	190	190	168	172	134	134	166	170	173	173	192	194	259	261	214	214	143	147	169	169
ONRC44	228	228	181	181	208	208	168	174	134	134	190	190	175	175	190	194	259	265	212	216	143	147	167	171
ONRC45	230	230	181	183	208	208	168	172	130	130	168	170	189	189	192	192	259	265	218	218	143	147	161	163
ONRC46	230	230	181	183	204	208	170	172	130	130	168	168	191	191	192	194	259	261	214	216	143	145	169	171
ONRC47	206	230	183	183	204	204	174	174	130	130	168	168	183	193	196	196	259	259	212	214	141	145	167	169
ONRC48	228	228	183	187	192	196	170	172	132	132	168	168	191	191	192	194	259	259	210	216	145	145	165	171
ONRC49	228	228	183	187	190	190	170	172	132	132	168	168	191	191	192	196	261	263	212	216	141	147	165	167
ONRC50	228	228	183	183	206	206	170	170	128	132	168	194	191	191	192	194	259	261	212	214	143	147	167	169
ONWL01	230	230	185	185	202	206	156	172	136	136	168	168	191	193	194	196	255	271	214	216	143	147	165	167
ONWL02	230	230	183	185	192	210	156	176	124	136	164	164	191	195	192	202	255	271	214	216	145	147	167	169
ONWL03	228	232	185	189	190	202	156	156	124	136	164	164	191	193	192	202	255	255	214	214	147	149	167	167
ONWL04	230	232	171	183	210	212	156	156	136	136	150	164	189	191	192	198	255	271	214	214	145	145	167	167
ONWL05	230	232	185	191	206	208	160	162	136	136	144	164	191	193	190	202	255	255	214	214	143	145	167	167
ONWL06	230	230	183	183	208	224	148	162	128	136	162	168	191	193	196	200	255	255	214	214	145	147	167	167
ONWL07	230	230	185	185	194	212	164	182	134	134	160	166	193	193	194	202	255	273	214	214	143	149	167	167
ONWL08	228	230	183	185	190	208	166	166	128	136	152	166	187	191	202	204	255	255	214	214	139	145	167	167
ONWL09	230	232	185	187	206	212	150	162	128	132	162	164	189	191	190	190	255	255	214	216	143	143	167	169
ONWL10	230	230	183	183	210	212	162	166	136	136	164	166	187	193	190	190	255	255	214	214	139	145	167	167
ONWL11	230	230	171	185	182	206	164	190	136	136	162	166	189	193	192	202	255	273	214	214	143	147	167	167
ONWL12	216	230	181	183	210	210	156	156	128	136	162	166	191	191	190	190	255	255	214	216	143	145	167	169
ONWL13	230	230	187	189	206	210	162	162	136	136	162	164	189	191	192	196	255	255	214	216	143	143	167	169
ONWL14	230	230	185	189	190	196	160	160	136	136	162	164	189	209	194	200	255	255	214	214	141	145	167	167
ONWL15	224	230	183	185	192	208	160	160	136	136	164	166	189	189	194	198	255	273	214	214	141	145	167	167
ONWL16	232	234	183	185	204	208	178	178	136	136	144	166	189	191	194	196	255	255	214	214	141	145	167	167
ONWL17	230	234	183	187	190	206	150	160	136	136	144	164	187	193	198	208	255	255	214	216	143	143	167	169
ONWL18	224	232	165	183	172	190	166	166	136	136	144	164	187	191	192	210	255	255	212	214	143	143	165	167
ONWL19	224	224	183	183	190	208	172	172	124	136	166	166	189	191	190	192	253	271	214	214	143	143	167	167
ONWL20	232	232	169	179	212	216	158	160	136	136	166	166	189	189	190	190	255	271	214	214	141	143	167	167
ONWL21	232	232	183	183	190	212	160	160	136	136	144	166	189	189	194	194	255	271	214	216	141	143	167	169
ONWL22	232	232	183	183	194	204	160	176	136	140	166	166	191	191	198	202	255	255	214	216	143	143	167	169
ONWL23	226	232	183	183	198	206	160	174	136	136	164	168	189	191	190	192	255	255	214	214	143	145	167	167
ONWL24	232	236	183	185	194	212	162	178	136	136	166	166	191	195	192	192	255	255	214	214	141	145	167	167
ONWL25	230	232	183	183	182	206	180	190	136	142	168	170	191	193	192	208	255	271	214	216	143	145	167	169
ONWL26	232	232	181	181	182	216	154	168	136	136	168	168	191	193	194	204	255	255	214	214	143	145	167	167
ONWL27	234	234	181	181	214	216	156	156	136	136	168	168	191	193	194	202	255	255	214	216	143	143	167	169
ONWL28	234	234	183	183	190	208	162	164	136	136	168	170	191	193	194	202	255	255	214	214	141	143	167	167
ONWL29	234	234	187	187	190	208	180	198	136	136	168	168	191	193	196	202	253	253	214	214	143	145	167	167
ONWL30	232	234	169	179	188	206	158	178	136	142	166	168	191	193	194	194	253	253	214	214	143	145	167	167
ONWL31	234	234	169	189	188	206	154	154	132	140	132	140	191	193	198	198	255	255	214	214	141	145	167	167
ONWL32	232	232	183	189	208	210	170	170	118	136	162	166	189	189	194	200	255	255	214	216	143	147	167	169
ONWL33	230	234	185	191	190	206	158	164	136	136	152	168	189	199	192	202	255	255	212	212	143	145	165	165
ONWL34	234	234	181	181	208	208	162	180	136	136	162	168	187	189	194	196	255	255	214	214	143	145	167	167
ONWL35	234	234	181	183	192	206	164	196	136	136	152	168	191	191	192	194	255	255	212	212	137	145	165	165
ONWL36	234	236	189	189	182	206	150	162	136	136	166	168	191	191	192	198	255	255	214	214	143	143	167	167
ONWL37	230	234	183	183	190	208	164	192	136	136	162	166	191	191	200	204	255	255	214	214	139	145	167	167
ONWL38	230	234	183	183	208	218	164	192	136	136	166	166	189	189	190	192	255	255	214	216	143	145	167	169
ONWL39	232	232	183	185	190	194	152	164	136	136	158	166	191	191	196	198	255	273	214	214	141	145	167	167
ONWL40	234	234	169	191	190	204	156	192	136	136	160	166	189	189	194	200	255	271	214	214	143	143	167	167
ONWL41	234	234	169	191	202	202	166	166	136	136	156	166	189	189	194	202	255	255	214	214	143	147	167	167
ONWL42	232	242	183	183	206	208	150	174	136	136	164	164	191	191	202	202	255	255	214	214	139	145	167	167
ONWL43	232	232	183	189	184	206	162	162	136	136	164	166	191	199	0	0	255	255	214	214	141	141	167	167
ONWL44	232	232	183	183	192	218	170	180	136	136	164	166	191	193	178	202	255	255	214	216	143	143	167	169
ONWL45	234	234	185	185	216	216	162	182	136	136	152	166	191	193	194	210	255	255	214	214	143	147	167	167
ONWL46	234	234	183	183	190	194	164	180	136	136	164	166	191	195	194	202	255	255	214	214	141	145	167	167
ONWL47	210	234	183	189	184	190	164	168	132	136	166	166	191	193	194	198	255	273	214	214	141	145	167	167
ONWL48	232	232	179	189	200	206	170	176	132	132	164	166	191	193	192	192	255	255	214	216	145	145	167	169
ONWL49	232	232	185	187	206	206	162	180	132	132	162	168	191	193	192	192	255	255	214	214	141	147	167	167
ONWL50	232	232	183	183	204	216	160	166	132	134	162	168	191	193	192	192	255	271	214	216	143	147	167	169
ONWLW01	228	232	193	197	200	204	154	166	122	126	164	166	181	191	194	198	265	269	216	218	147	151	167	169
ONWLW02	226	228	195	197	202	208	148	152	126	128	166	166	195	195	194	196	257	263	214	216	143	145	165	167
ONWLW03	230	234	195	197	200	204	148	148	124	126	166	166	193	193	190	198	253	253	212	214	141	145	163	165
ONWLW04	228	232	193	197	200	204	154	166	122	126	164	166	181	191	194	198	265	269	216	218	147	151	167	169
ONWLW05	228	228	179	187	204	206	158	158	130	132	166	168	191	191	194	196	259	259	218	222	145	147	171	175
ONWLW06	228	230	195	197	196	198	150	158	128	128	164	166	191	191	194	198	257	273	214	216	145	151	165	167
ONWLW07	224	228	183	187	206	210	158	158	132	134	164	166	193	209	160	186	263	265	214	216	141	145	167	169
ONWLW08	226	230	179	187	204	206	158	158	130	132	166	168	191	191	194	196	259	259	218	222	145	147	171	175
ONWLW09	226	228	193	195	212	212	150	156	118	118	164	166	189	193	188	194	255	255	214	216	145	149	165	167
ONWLW10	228	230	191	193	184	202	148	162	126	128	166	166	191	191	196	198	253	253	212	218	149	153	163	169
ONWLW11	226	228	183	187	204	208	158	174	128	146	166	168	193	193	188	188	259	271	212	216	141	145	165	169
ONWLW12	226	232	183	195	208	212	148	152	122	126	166	168	191	193	190	194	257	259	214	220	143	145	163	167
ONWLW13	224	228	193	195	198	200	146	166	126	128	166	166	191	191	198	202	253	253	212	214	147	153	163	165
ONWLW14	226	230	183	187	204	208	158	174	128	146	166	168	193	193	188	188	259	271	212	216	141	145	165	169
ONWLW15	226	228	193	195	206	212	152	158	124	126	164	168	189	193	188	194	255	259	214	218	147	149	165	167
ONWLW16	228	230	185	187	206	210	174	178	134	136	166	166	191	201	190	192	259	263	214	216	143	145	167	169
ONWLW17	228	230	185	187	206	210	174	178	134	136	166	166	191	201	190	192	259	263	214	216	143	145	167	169
ONWLW18	230	230	195	197	202	202	152	158	126	130	166	168	191	195	194	196	259	263	214	216	147	153	165	167
ONWLW19	224	228	197	199	200	204	150	164	124	126	166	166	193	201	192	198	257	267	212	214	149	151	163	165
ONWLW20	228	230	183	183	202	210	156	174	130	140	166	166	193	193	188	192	261	277	214	218	143	149	167	171
ONWLW21	224	228	193	197	200	204	154	166	122	126	164	166	181	191	194	198	265	269	216	218	147	151	167	169
ONWLW22	224	230	183	187	208	212	162	164	126	128	166	192	193	201	192	194	259	279	214	216	143	147	167	169
ONWLW23	226	234	195	195	182	204	150	162	122	126	166	168	191	195	166	194	253	253	212	214	141	141	163	165
ONWLW24	226	232	195	197	202	208	148	152	126	128	166	166	195	195	194	196	257	263	214	216	143	145	165	167
ONWLW25	226	228	183	187	208	212	162	164	126	128	166	192	193	201	192	194	259	279	214	216	143	147	167	169
ONWLW26	228	228	195	195	188	206	148	154	122	126	164	164	187	191	160	188	255	255	214	218	147	149	165	165
ONWLW27	226	230	193	193	204	206	148	148	126	128	166	166	189	189	196	198	259	271	214	218	149	151	165	169
ONWLW28	226	228	195	201	200	200	168	170	126	126	168	168	193	193	196	198	259	263	214	218	147	149	165	169
ONWLW29	224	228	191	197	188	204	144	148	126	128	166	166	189	193	196	200	265	267	214	216	149	151	165	167
ONWLW30	226	228	185	185	206	208	154	158	130	132	166	166	189	189	190	194	267	269	214	218	141	145	167	171
ONWLW31	224	230	183	187	206	210	158	158	132	134	164	166	193	209	160	186	263	265	214	216	141	145	167	169
ONWLW32	232	232	185	187	204	208	156	158	132	134	166	166	191	191	188	198	259	279	214	216	143	145	167	169
ONWLW33	226	228	183	185	208	212	160	186	132	134	162	164	191	193	188	194	263	263	214	216	145	145	167	169
ONWLW34	226	230	183	187	206	208	172	172	124	128	166	168	193	193	192	194	263	265	216	218	145	147	169	171
ONWLW35	228	234	195	203	198	204	146	148	118	126	166	168	193	201	194	202	259	265	200	216	149	149	151	167
ONWLW36	224	228	183	187	204	208	158	174	128	146	166	168	193	193	188	188	259	271	212	216	141	145	165	169
ONWLW37	228	232	183	185	204	208	156	156	128	132	166	168	191	193	192	196	265	269	216	220	143	145	169	173
ONWLW38	226	230	197	199	200	204	150	164	124	126	166	166	193	201	192	198	257	267	212	214	149	151	163	165
ONWLW39	228	228	183	185	208	212	160	186	132	134	162	164	191	193	188	194	263	263	214	216	145	145	167	169
ONWLW40	226	230	197	197	190	206	156	162	120	130	164	166	191	195	188	204	255	257	214	218	145	149	151	165
ONWLW41	226	230	193	193	204	206	148	148	126	128	166	166	189	189	196	198	259	271	214	218	149	151	165	169
ONWLW42	226	234	191	197	188	204	144	148	126	128	166	166	189	193	196	200	265	267	214	216	149	151	165	167
ONWLW43	228	228	195	197	208	212	150	154	124	126	166	168	191	193	190	194	257	261	212	216	145	149	163	167
ONWLW44	226	230	197	197	198	202	148	164	124	128	166	166	191	191	192	194	263	265	214	220	143	149	165	171
ONWLW45	224	228	183	183	202	210	156	174	130	140	166	166	193	193	188	192	261	277	214	218	143	149	167	171
ONWLW46	228	232	175	187	206	212	156	160	126	128	166	192	191	191	190	192	259	263	214	216	143	149	167	169
ONWLW47	228	232	183	185	206	210	158	174	126	146	166	168	191	191	190	196	261	267	216	218	143	149	169	171
ONWLW48	228	228	175	187	206	212	156	160	126	128	166	192	191	191	190	192	259	263	214	216	143	149	167	169

ONWLW49	228	232	185	187	204	208	156	158	132	134	166	166	191	191	188	198	259	279	214	216	143	145	167	169
ONWLW50	226	230	185	187	204	208	156	158	132	134	166	166	191	191	188	198	259	279	214	216	143	145	167	169
ONTO01	230	232	183	187	206	208	156	160	132	138	166	168	191	193	188	192	257	271	214	216	139	145	167	169
ONTO02	230	230	183	183	202	204	156	160	120	136	164	168	189	193	190	192	255	271	214	214	145	147	167	167
ONTO03	230	230	181	185	200	208	160	164	136	162	166	168	189	193	194	200	257	255	214	216	147	149	167	169
ONTO04	216	230	179	183	176	208	154	154	120	132	164	168	191	193	192	196	255	271	212	214	145	145	165	167
ONTO05	230	230	185	185	194	212	152	160	134	138	166	168	189	193	186	188	255	255	212	214	143	145	165	167
ONTO06	230	230	181	185	200	222	152	160	132	134	166	168	187	189	188	190	255	255	214	214	145	147	167	167
ONTO07	224	230	183	187	206	208	152	160	130	132	166	168	189	191	194	200	257	273	214	214	141	149	167	167
ONTO08	228	230	183	185	204	206	152	160	130	134	166	168	191	191	192	196	255	255	212	214	139	145	165	167
ONTO09	230	232	183	187	202	214	152	160	136	136	166	168	189	189	186	188	255	255	214	216	143	143	167	169
ONTO10	230	230	183	187	206	206	138	138	132	136	164	166	189	191	188	190	255	255	210	214	137	145	163	167
ONTO11	230	230	185	187	182	212	158	164	134	134	164	168	189	195	188	190	257	273	212	214	143	147	165	167
ONTO12	216	230	187	191	194	194	156	164	132	136	166	170	187	189	176	190	255	255	212	214	143	145	165	167
ONTO13	230	230	181	193	206	210	162	164	134	138	164	166	189	191	192	194	255	255	214	214	143	143	167	167
ONTO14	230	230	183	187	206	210	162	166	136	140	166	168	191	193	194	200	255	255	210	214	139	145	163	167
ONTO15	224	230	191	197	208	210	162	164	134	136	164	168	189	191	192	196	257	273	212	214	141	145	165	167
ONTO16	232	234	191	199	202	210	162	166	140	144	166	168	187	191	186	188	255	255	214	216	141	145	167	169
ONTO17	230	234	185	187	198	206	162	164	132	134	164	168	189	191	188	190	255	255	212	216	143	143	165	169
ONTO18	224	232	185	189	174	204	162	162	130	160	166	178	189	191	188	190	255	255	214	214	139	143	167	167
ONTO19	224	224	187	191	202	208	158	162	132	136	164	168	195	197	188	190	257	271	216	216	143	143	169	169
ONTO20	232	232	185	189	202	208	152	162	134	138	164	168	189	193	188	190	255	271	214	216	139	143	167	169
ONTO21	232	232	183	189	202	208	154	164	132	136	166	168	189	191	176	190	255	271	212	214	141	143	165	167
ONTO22	232	232	185	193	200	210	156	158	130	132	164	168	189	191	192	194	255	255	214	214	143	143	167	167
ONTO23	226	232	181	187	202	204	148	154	128	134	166	168	191	197	194	200	253	255	214	216	143	147	167	169
ONTO24	232	236	183	185	208	212	152	160	130	136	164	168	193	195	192	196	255	255	214	216	141	145	167	169
ONTO25	230	232	185	191	208	214	138	138	130	134	164	170	191	193	186	188	255	271	212	214	143	145	165	167
ONTO26	232	232	183	187	176	200	158	164	132	136	168	186	189	191	188	190	255	255	214	218	143	145	167	171
ONTO27	234	234	181	187	198	204	156	164	132	140	164	166	189	193	188	190	253	255	214	216	139	143	167	169
ONTO28	234	234	183	185	198	200	144	156	134	136	166	168	193	195	176	190	255	255	214	214	141	143	167	167
ONTO29	234	234	183	191	200	206	162	164	134	138	166	168	189	193	192	194	253	253	214	216	143	145	167	169
ONTO30	232	234	183	187	178	204	162	166	120	134	164	168	189	193	194	200	251	253	214	214	143	145	167	167
ONTO31	234	234	181	187	172	202	162	164	134	142	162	170	191	193	192	196	255	255	214	216	139	145	167	169
ONTO32	232	232	181	185	188	188	162	166	138	142	166	168	191	197	186	188	255	255	214	216	143	147	167	169
ONTO33	230	234	183	189	204	208	162	164	132	136	168	184	189	193	188	190	251	255	214	216	143	145	167	169
ONTO34	234	234	185	189	204	204	162	162	128	132	164	168	193	193	198	200	255	255	212	216	143	145	165	169
ONTO35	234	234	181	187	200	204	162	164	130	136	166	168	187	193	186	192	255	255	214	216	137	145	167	169
ONTO36	234	236	183	187	202	204	164	164	134	138	166	168	191	193	188	192	251	255	214	216	143	143	167	169
ONTO37	230	234	185	189	198	200	162	162	130	134	166	168	189	193	190	192	255	255	214	216	139	145	167	169
ONTO38	230	234	183	189	190	194	162	164	130	132	166	170	191	195	188	190	255	255	214	214	143	145	167	167
ONTO39	232	232	187	193	170	186	164	172	128	134	166	168	191	195	188	190	247	273	214	216	141	145	167	169
ONTO40	234	234	187	197	186	190	162	162	130	132	166	168	191	197	176	190	255	271	214	214	139	143	167	167
ONTO41	234	234	187	193	182	206	166	174	130	134	166	170	191	195	192	194	255	255	214	216	143	147	167	169
ONTO42	232	242	187	193	196	206	168	168	128	132	166	168	193	197	194	200	253	255	212	214	139	145	165	167
ONTO43	232	232	179	183	202	206	170	170	132	134	166	168	191	195	192	196	255	255	212	214	141	141	165	167
ONTO44	232	232	181	185	172	172	158	158	128	134	166	168	191	199	186	188	255	255	212	216	143	143	165	169
ONTO45	232	232	185	189	198	202	154	158	132	136	168	168	201	203	188	190	251	255	214	216	143	147	167	169
ONTO46	232	232	181	193	202	204	158	158	132	136	168	170	195	197	196	198	255	255	214	214	141	145	167	167
ONTO47	226	232	183	187	200	206	158	158	132	138	166	168	191	195	196	202	257	273	214	216	141	145	167	169
ONTO48	232	236	181	185	202	206	158	160	130	136	168	168	193	195	186	190	255	255	214	214	145	145	167	167
ONTO49	230	232	179	189	204	208	158	160	132	134	168	170	193	197	186	194	255	255	216	218	141	147	169	171
ONTO50	232	232	183	187	194	200	154	158	132	136	166	170	193	195	192	200	247	271	214	216	143	147	167	169
MEEB01	228	228	187	189	188	206	158	174	130	132	160	164	191	199	190	198	257	257	216	218	141	143	167	169
MEEB02	214	214	185	193	208	208	160	174	128	132	160	166	187	193	184	192	259	259	212	216	137	145	163	167
MEEB03	230	230	187	187	178	204	156	174	138	138	162	164	191	201	184	192	257	257	216	216	137	145	167	167
MEEB04	228	230	187	187	178	204	158	176	138	140	162	166	189	191	186	192	257	261	216	218	139	145	167	169
MEEB05	230	230	185	191	196	196	156	174	118	130	164	164	191	193	190	192	255	255	212	214	139	145	163	165
MEEB06	236	238	185	187	180	204	160	174	134	138	164	164	187	191	188	194	255	257	200	216	139	145	151	167
MEEB07	232	232	187	187	200	200	160	168	130	134	162	164	189	195	192	196	257	259	216	218	139	145	167	169
MEEB08	230	230	185	187	188	214	172	172	140	142	164	164	189	197	190	198	255	255	218	220	139	145	169	171
MEEB09	234	240	185	189	214	214	158	162	126	130	164	164	191	195	194	198	261	263	216	218	137	145	167	169
MEEB10	232	240	189	189	214	214	158	160	136	136	164	164	191	195	190	194	261	261	214	216	139	145	165	167
MEEB11	222	222	185	189	214	214	162	162	128	130	164	164	189	197	192	194	261	261	214	214	139	145	165	165
MEEB12	234	238	187	189	178	204	156	158	132	138	162	164	187	191	190	196	257	257	216	216	137	145	167	167
MEEB13	230	230	187	189	174	204	166	168	130	132	164	164	187	191	188	192	255	257	216	218	139	145	167	169
MEEB14	232	232	173	187	214	214	158	158	136	140	164	164	189	197	188	194	261	261	214	214	139	145	165	165
MEEB15	230	230	183	187	196	196	156	170	132	134	164	164	187	195	186	190	253	253	214	214	139	145	165	165
MEEB16	230	236	187	187	178	204	158	176	128	136	164	164	191	193	192	192	257	257	216	218	139	145	167	169
MEEB17	230	238	187	189	180	204	158	182	132	134	162	164	189	195	190	192	255	255	214	214	139	145	165	165
MEEB18	224	230	185	193	186	186	158	172	126	130	162	164	189	193	192	196	259	259	214	216	137	145	165	167
MEEB19	232	232	189	191	182	204	158	174	132	136	162	164	187	191	186	194	257	257	216	218	141	145	167	169
MEEB20	230	232	185	187	202	226	158	158	132	134	164	166	187	195	188	192	259	259	216	216	139	145	167	167
MEEB21	230	236	187	189	178	188	158	176	130	138	164	164	189	193	192	192	257	257	216	218	139	145	167	169
MEEB22	232	238	187	189	188	214	158	158	138	138	164	166	191	191	188	194	261	261	214	214	139	145	165	165
MEEB23	232	232	185	187	182	210	158	174	132	132	164	164	189	193	186	192	259	259	214	214	139	145	165	165
MEEB24	214	214	185	193	178	208	156	160	128	130	162	162	189	197	192	196	259	259	216	218	139	145	167	169
MEEB25	232	232	185	185	184	212	160	174	130	134	162	162	189	197	190	192	259	259	218	218	137	145	169	169
MEEB26	246	246	185	185	182	218	174	176	130	158	164	164	189	197	188	194	259	259	216	218	139	145	167	169
MEEB27	232	232	185	187	190	216	156	158	126	130	164	164	189	197	184	192	259	259	216	216	139	145	167	167
MEEB28	232	238	183	185	190	216	158	160	132	134	164	164	191	193	186	192	259	259	218	218	139	145	169	169
MEEB29	230	230	185	187	182	198	158	172	112	134	162	164	187	197	188	192	257	259	194	214	139	145	165	165
MEEB30	232	232	185	189	188	214	158	168	126	130	164	164	189	197	188	194	259	259	202	216	139	145	153	167
MEEB31	230	230	171	187	182	204	162	176	130	132	162	166	187	191	186	194	259	259	208	216	139	145	159	167
MEEB32	230	236	187	189	180	204	158	174	132	136	162	164	187	195	184	190	255	257	214	214	139	147	165	165
MEEB33	230	238	185	187	180	204	158	158	128	132	164	164	189	197	188	194	255	255	214	218	137	145	165	169
MEEB34	224	230	185	187	180	204	158	182	130	130	164	166	189	195	184	192	255	255	216	216	139	145	167	167
MEEB35	214	214	185	187	200	200	160	174	130	134	164	164	189	193	188	190	255	257	200	214	139	145	151	165
MEEB36	232	232	187	189	178	188	160	174	130	132	162	162	187	189	190	194	257	257	216	216	139	145	167	167
MEEB37	230	230	183	191	198	224	158	160	112	130	162	166	189	193	190	196	257	257	214	216	139	145	165	167
MEEB38	228	230	185	187	196	196	158	158	128	134	162	166	187	191	184	192	255	259	214	216	141	147	165	167
MEEB39	230	230	189	189	178	178	158	174	128	132	160	166	187	193	192	196	259	259	216	218	139	145	167	169
MEEB40	220	232	185	187	190	218	156	160	132	134	164	164	193	201	190	192	259	259	214	214	139	145	165	165
MEEB41	222	222	187	189	200	200	158	174	130	130	164	166	191	193	188	192	255	255	216	216	137	145	167	167
MEEB42	230	232	185	185	188	214	160	160	142	146	0	0	189	197	190	192	259	259	214	214	139	145	165	165
MEEB43	228	230	189	189	178	188	156	158	132	134	164	166	187	197	190	192	257	257	216	216	137	145	167	167
MEEB44	228	230	187	187	204	204	166	174	128	134	162	162	187	191	186	194	259	261	214	216	139	145	165	167
MEEB45	222	222	185	189	186	218	158	170	130	158	164	164	189	195	188	194	259	259	216	218	139	147	167	169
MEEB46	218	230	185	187	208	208	158	172	128	130	162	166	189	193	192	198	259	259	214	218	139	145	165	169
MEEB47	246	246	187	193	178	210	158	174	130	136	164	166	189	193	186	192	259	259	214	216	139	145	165	167
MEEB48	230	232	189	189	182	204	158	176	132	134	160	164	189	201	194	194	257	257	216	216	139	145	167	167
MEEB49	230	230	187	189	178	188	168	176	128	132	162	164	189	195	192	196	257	257	204	216	139	147	155	167
MEEB50	226	232	185	187	188	214	174	174	140	144	164	164	189	197	190	194	259	261	212	214	139	145	163	165
MEBSP01	228	232	187	189	188	206	158	174	130	132	162	164	189	193	190	194	257	257	218	218	139	145	169	169
MEBSP02	220	220	185	187	182	210	158	174	132	132	164	180	189	195	194	206	259	259	214	214	137	145	165	165
MEBSP03	230	230	171	187	182	204	162	176	124	130	162	164	189	193	188	194	259	259	216	216	139	145	167	167
MEBSP04	222	222	187	189	178	208	174	176	128	130	164	164	187	193	186	190	259	259	214	214	139	145	165	165
MEBSP05	238	238	175	189	178	204	158	160	126	126	164	168	191	195	188	198	257	257	214	216	139	145	165	167
MEBSP06	232	238	183	185	190	208	158	160	120	124	162	166	197	205	188	192	253	253	214	214	141	145	165	165
MEBSP07	230	230	185	187	178	188	148	162	128	132	164	164	187	197	188	192	251	251	214	216	139	145	165	167
MEBSP08	230	236	185	187	194	208	160	174	130	132	142	166	187	195	184	190	255	257	214	216	137	145	165	167
MEBSP09	238	238	175	189	214	214	158	162	126	130	164	166	187	197	190	198	261	263	216	218	137	145	167	169
MEBSP10	230	230	183	187	196	196	156	170	132	134	164	166	193	195	190	200	253	253	214	214	139	145	165	165
MEBSP11	236	236	175	187	178	188	148	160	122	124	160	164	195	195	190	198	251	253	216	218	139	145	167	169
MEBSP12	244	244	187	193	178	210	158	166	130	136	164	166	191	193	188	196	259	259	214	214	139	145	165	165
MEBSP13	230	234	187	187	204	204	166	174	126	128	164	164	193	193	190	196	259	261	216	216	137	145	167	167
MEBSP14	232	232	175	187	200	200	160	168	130	134	164	164	187	191	188	202	253	255	212	216	141	141	163	167
MEBSP15	230	230	187	189	178	188	148	162	130	132	164	166	191	193	194	198	251	251	216	216	139	145	167	167
MEBSP16	234	238	187	189	210	212	156	158	130	132	164	164	189	193	160	188	257	257	214	218	137	137	165	169
MEBSP17	232	232	185	187	208	210	156	158	126	130	162	164	191	193	188	192	259	259	214	216	139	145	165	167
MEBSP18	230	230	185	187	174	204	150	162	130	132	164	164	191	203	188	204	251	251	214	216	137	141	165	167
MEBSP19	228	230	185	187	200	200	160	174	130	134	162	166	191	195	182	196	251	251	216	216	137	141	167	167
MEBSP20	238	238	187	189	204	208	158	182	132	134	164	164	193	195	160	188	255	255	214	214	139	145	165	165
MEBSP21	228	230	185	187	196	196	158	158	128	134	162	164	191	193	190	198	253	253	212	214	139	145	163	165

MEBSP22	232	232	187	189	208	208	146	162	128	130	164	166	199	203	192	198	259	259	214	214	139	145	165	165
MEBSP23	226	232	185	187	208	208	174	174	140	144	160	164	191	203	188	196	259	261	214	218	139	145	165	169
MEBSP24	220	232	185	187	176	176	156	160	122	124	164	164	193	195	188	192	253	255	214	214	139	145	165	165
MEBSP25	232	232	187	189	186	186	160	160	126	130	164	166	189	191	190	198	259	259	214	216	137	145	165	167
MEBSP26	230	232	189	189	182	204	158	176	132	134	162	164	191	193	188	206	251	255	216	216	139	145	167	167
MEBSP27	228	230	189	189	178	178	158	174	128	132	162	166	191	193	188	192	259	259	216	216	139	145	167	167
MEBSP28	232	232	185	189	206	210	158	168	130	132	164	164	187	195	188	200	259	259	214	214	139	145	165	165
MEBSP29	230	230	187	187	178	204	144	158	122	122	162	164	191	195	192	198	257	257	214	216	139	145	165	167
MEBSP30	230	236	187	189	206	210	158	174	132	136	164	164	193	195	188	198	255	257	214	216	139	145	165	167
MEBSP31	232	240	187	189	214	214	158	160	136	136	162	166	187	197	182	192	261	261	216	216	137	145	167	167
MEBSP32	232	238	187	189	188	214	158	158	138	138	162	164	189	191	192	196	261	261	216	216	139	145	167	167
MEBSP33	230	230	185	187	182	198	158	172	112	134	164	164	193	195	190	194	251	251	214	214	139	145	165	165
MEBSP34	238	238	185	187	206	212	158	158	128	132	162	166	189	193	194	198	255	255	214	214	139	145	165	165
MEBSP35	232	232	175	187	208	210	174	176	130	158	162	164	187	195	188	194	253	253	218	218	139	145	169	169
MEBSP36	232	232	189	189	214	214	158	158	136	140	164	164	191	199	188	200	261	261	218	218	137	137	169	169
MEBSP37	230	232	175	187	202	226	164	166	132	134	164	166	187	197	186	190	259	259	214	214	139	145	165	165
MEBSP38	238	238	185	187	178	188	158	168	126	126	164	166	191	197	188	206	257	257	214	214	139	145	165	165
MEBSP39	214	214	175	187	214	214	162	162	128	130	164	164	191	195	190	204	253	253	216	216	137	145	167	167
MEBSP40	232	232	185	189	198	208	158	170	130	132	160	164	187	195	186	192	253	253	216	216	139	145	167	167
MEBSP41	222	224	187	189	200	200	158	174	130	130	164	164	191	195	188	198	255	255	214	214	139	145	165	165
MEBSP42	230	230	185	191	196	196	156	174	118	130	162	166	187	195	188	192	251	251	212	214	139	145	163	165
MEBSP43	230	230	187	189	198	224	158	160	112	130	164	168	191	193	188	196	251	251	214	216	139	145	165	167
MEBSP44	228	230	187	187	202	208	160	174	130	132	164	164	199	209	188	196	257	261	214	216	139	145	165	167
MEBSP45	230	230	185	187	208	210	172	172	140	142	164	164	197	199	194	196	255	255	216	216	137	145	167	167
MEBSP46	230	230	189	191	182	204	144	160	132	136	164	168	187	195	186	190	251	251	216	216	139	145	167	167
MEBSP47	246	246	185	185	188	192	160	174	130	134	164	164	191	191	184	198	259	259	214	214	139	145	165	165
MEBSP48	230	232	175	187	208	208	166	166	128	132	162	168	191	195	184	196	259	259	214	216	139	145	165	167
MEBSP49	230	232	185	185	208	210	160	160	142	146	164	164	193	201	190	198	259	259	214	216	139	145	165	167
MEBSP50	222	222	185	187	192	200	158	182	130	130	164	164	191	191	196	196	255	255	214	214	139	145	165	165
MASB01	228	228	187	187	186	206	158	172	128	132	168	186	193	201	192	200	257	257	214	214	139	145	165	165
MASB02	230	230	187	187	206	208	158	158	128	132	170	188	191	193	162	192	259	259	212	214	139	145	163	165
MASB03	228	230	187	189	204	208	158	172	126	130	166	188	193	203	162	190	259	261	212	214	139	145	163	165
MASB04	230	230	187	189	174	210	158	202	134	138	170	188	193	195	160	190	259	259	212	214	137	143	163	165
MASB05	230	230	187	187	174	212	158	172	128	136	170	188	193	195	160	190	255	257	212	212	137	145	163	163
MASB06	230	230	187	189	208	208	158	174	130	132	170	188	193	193	160	188	255	271	212	212	137	145	163	163
MASB07	230	230	187	189	192	206	158	174	130	132	170	188	193	195	160	188	255	255	212	212	141	145	163	163
MASB08	230	230	187	189	202	202	160	172	118	118	170	188	193	195	160	190	255	259	212	212	141	145	163	163
MASB09	230	230	187	187	186	226	186	198	120	132	170	188	193	195	160	188	255	261	212	212	141	145	163	163
MASB10	228	230	187	189	182	186	158	198	130	134	170	188	189	191	164	190	253	257	212	212	137	141	163	163
MASB11	230	236	187	189	192	204	158	176	132	138	176	194	191	193	192	200	257	257	210	212	137	145	161	163
MASB12	230	236	187	189	202	206	158	194	130	134	170	194	191	191	164	194	257	257	210	210	141	145	161	161
MASB13	230	230	177	187	208	216	158	194	122	146	170	188	171	193	192	200	257	257	212	214	139	145	163	165
MASB14	234	238	187	189	190	208	160	186	128	130	174	194	191	193	168	190	253	253	212	212	139	145	163	163
MASB15	228	230	187	187	194	206	158	194	130	134	170	188	191	193	188	202	253	255	210	210	139	145	161	161
MASB16	230	236	187	189	202	206	158	176	132	132	170	194	193	195	188	202	253	253	212	214	141	145	163	165
MASB17	230	236	187	189	194	204	158	184	130	134	170	194	193	193	160	188	253	253	214	214	137	139	165	165
MASB18	230	230	185	187	200	204	158	158	146	146	170	188	193	193	188	192	253	257	214	214	139	145	165	165
MASB19	230	230	187	187	202	204	160	174	132	132	170	188	193	195	188	198	255	255	212	212	139	145	163	163
MASB20	230	230	187	187	170	206	158	180	128	134	170	188	193	195	190	194	255	255	212	212	135	145	163	163
MASB21	230	230	187	187	170	204	160	174	132	146	170	188	195	197	160	188	255	255	212	214	139	145	163	165
MASB22	228	230	187	189	202	212	190	200	130	136	170	188	193	195	192	206	255	257	216	216	137	139	167	167
MASB23	230	230	187	187	170	206	160	166	130	142	168	186	195	195	192	206	255	255	212	212	141	145	163	163
MASB24	230	230	187	187	204	204	158	192	130	132	168	186	193	195	188	188	253	253	212	212	139	139	163	163
MASB25	228	230	187	187	188	190	160	192	126	132	168	188	193	195	160	188	255	259	214	214	141	145	165	165
MASB26	230	230	187	187	188	204	160	174	130	132	170	188	193	193	162	192	261	261	212	214	139	141	163	165
MASB27	230	230	187	189	182	208	158	174	130	132	170	188	193	193	194	200	261	261	212	214	139	145	163	165
MASB28	232	232	187	189	190	206	158	172	140	146	170	188	193	193	164	206	259	261	212	212	141	145	163	163
MASB29	230	232	187	189	182	202	158	176	134	134	172	190	193	197	190	198	259	259	212	214	141	145	163	165
MASB30	230	232	187	187	210	210	158	158	132	136	172	190	193	193	192	212	257	273	212	214	139	145	163	165
MASB31	232	232	179	189	182	182	192	196	132	146	172	196	189	191	162	194	259	259	212	214	137	137	163	165
MASB32	232	232	187	189	194	206	158	174	132	134	170	188	189	191	178	190	259	259	212	214	139	145	163	165
MASB33	232	232	187	187	198	210	158	160	146	154	170	188	191	191	160	188	259	259	214	214	139	145	165	165
MASB34	232	232	187	189	208	208	166	174	148	154	170	188	191	191	162	190	259	259	212	212	141	145	163	163
MASB35	232	232	187	189	192	192	158	174	122	134	172	190	189	193	162	192	259	259	212	212	139	145	163	163
MASB36	232	232	189	191	182	222	168	172	130	134	172	190	197	197	162	192	259	259	212	212	137	139	163	163
MASB37	232	232	187	189	208	208	160	174	132	140	172	190	195	195	190	200	253	277	214	214	141	145	165	165
MASB38	232	232	187	199	194	208	166	168	120	132	172	190	195	197	192	196	253	253	212	214	141	145	163	165
MASB39	232	238	191	199	208	208	174	194	128	130	172	196	195	197	192	192	253	253	212	214	141	145	163	165
MASB40	220	232	187	201	196	208	158	174	130	148	158	190	187	191	162	196	253	255	214	214	139	145	165	165
MASB41	232	232	187	189	192	192	160	174	132	132	172	190	187	197	162	194	253	253	212	212	139	145	163	163
MASB42	232	232	189	189	208	208	158	190	132	134	172	190	189	193	202	202	253	253	212	214	139	139	163	165
MASB43	234	234	189	191	186	210	174	192	132	132	172	190	191	193	192	200	253	257	212	212	139	139	163	163
MASB44	234	240	189	191	210	210	174	192	128	130	174	198	191	201	162	192	261	263	212	212	139	145	163	163
MASB45	232	240	189	191	192	212	160	194	136	146	172	198	187	191	190	202	261	261	212	212	139	145	163	163
MASB46	232	232	187	191	208	208	158	190	134	134	174	192	189	191	190	200	261	261	212	212	139	145	163	163
MASB47	232	238	199	199	210	212	156	194	130	130	172	198	187	191	162	194	261	261	212	212	141	145	163	163
MASB48	226	232	187	189	184	210	172	190	132	134	164	190	187	195	162	192	259	261	212	212	137	137	163	163
MASB49	230	232	187	189	194	198	158	190	128	132	170	188	189	195	194	200	259	259	212	214	139	145	163	165
MASB50	230	230	187	189	210	210	160	174	132	132	170	188	189	197	162	198	255	255	212	212	139	145	163	163
NYCM01	228	228	177	185	190	190	158	186	138	142	182	194	193	201	192	196	243	257	212	212	143	145	163	163
NYCM02	228	230	185	187	204	208	158	192	132	134	180	184	189	191	192	196	243	257	206	214	139	145	157	165
NYCM03	228	228	187	187	202	210	160	164	132	134	162	184	191	197	200	208	243	253	216	220	139	145	167	171
NYCM04	228	230	177	191	204	204	154	158	138	144	182	194	189	191	192	196	239	257	220	220	137	143	171	171
NYCM05	228	228	191	193	184	208	158	174	144	144	182	186	191	193	184	202	237	253	214	216	141	145	165	167
NYCM06	230	234	185	191	190	210	158	176	138	144	184	184	191	191	194	194	239	253	216	216	137	145	167	167
NYCM07	228	228	175	185	172	208	158	194	122	138	160	184	171	193	164	194	239	257	216	216	141	143	167	167
NYCM08	228	230	175	189	210	210	160	182	134	134	162	182	191	193	194	196	239	259	218	220	141	145	169	171
NYCM09	228	230	177	187	204	208	166	166	126	128	186	196	191	193	192	204	239	245	218	220	141	143	169	171
NYCM10	230	230	187	189	182	186	160	176	128	128	154	186	191	191	202	202	239	245	218	220	143	143	169	171
NYCM11	230	230	187	193	208	212	160	198	132	132	188	188	191	193	196	198	237	251	218	220	139	145	169	171
NYCM12	230	232	173	187	210	214	160	176	132	132	160	186	191	193	166	192	241	253	218	224	141	145	169	175
NYCM13	230	230	187	187	208	208	162	180	128	130	184	188	189	191	166	202	241	257	220	220	139	145	171	171
NYCM14	230	236	187	189	192	210	160	160	128	132	180	186	189	191	190	202	241	257	220	220	139	145	171	171
NYCM15	230	230	187	187	192	194	162	178	130	132	164	184	193	195	192	196	241	257	220	222	143	143	171	173
NYCM16	230	230	187	187	192	210	160	178	128	132	180	184	193	195	194	200	239	257	218	222	143	145	169	173
NYCM17	230	230	189	193	192	202	160	192	154	154	184	186	193	193	192	198	241	257	220	222	137	139	171	173
NYCM18	230	230	177	187	206	208	160	168	154	154	184	198	193	193	194	194	239	257	218	222	143	145	169	173
NYCM19	230	230	189	191	192	208	162	178	132	132	184	184	193	195	200	202	241	249	218	222	139	145	169	173
NYCM20	228	228	187	195	208	212	158	176	130	130	184	186	193	195	194	194	239	257	216	220	135	145	167	171
NYCM21	222	228	179	187	188	208	160	162	124	130	184	186	195	197	190	190	237	261	218	218	139	145	169	169
NYCM22	228	228	179	187	192	214	156	166	124	146	182	186	193	195	194	196	239	261	218	218	141	143	169	169
NYCM23	222	228	185	189	208	212	160	174	118	130	184	184	193	193	194	196	239	257	220	220	141	145	171	171
NYCM24	228	228	185	185	176	186	160	166	120	130	180	186	191	193	194	196	237	257	218	220	139	139	169	171
NYCM25	228	228	177	187	206	210	160	190	136	140	182	186	191	191	192	200	239	255	218	218	141	145	169	169
NYCM26	230	230	177	189	186	210	162	178	136	138	162	184	189	191	192	192	241	255	218	222	139	141	169	173
NYCM27	230	230	187	187	186	208	162	178	134	140	156	184	189	193	198	200	241	251	220	222	139	145	171	173
NYCM28	230	236	187	187	178	208	162	176	132	134	186	186	193	195	202	202	239	253	222	222	141	145	173	173
NYCM29	230	230	187	187	208	222	158	166	132	136	164	186	191	193	192	194	237	255	222	226	141	145	173	177
NYCM30	228	230	187	187	206	210	162	166	132	132	164	184	189	191	192	196	237	257	222	226	143	145	173	177
NYCM31	228	230	187	189	178	182	160	174	134	138	186	190	189	193	192	202	241	257	224	226	137	137	175	177
NYCM32	228	230	185	187	190	208	162	174	128	134	180	186	193	193	192	198	239	253	222	224	139	145	173	175
NYCM33	230	236	185	193	204	208	174	178	132	134	156	182	195	195	192	202	239	251	220	220	139	145	171	171
NYCM34	224	230	185	193	190	192	162	176	132	134	160	184	197	197	192	200	243	253	220	220	141	143	171	171
NYCM35	230	230	185	187	192	192	158	176	128	130	180	188	195	197	188	192	243	253	232	240	139	145	167	169
NYCM36	232	232	187	189	192	208	162	172	130	134	182	186	197	197	190	194	243	253	222	232	137	139	173	183
NYCM37	232	248	185	187	192	210	172	180	134	148	158	186	195	195	190	192	243	253	222	224	141	145	173	175
NYCM38	230	230	185	185	192	208	158	178	130	130	184	186	195	197	192	192	243	253	222	224	143	145	173	175
NYCM39	230	230	183	187	192	206	158	176	130	130	182	186	189	193	190	204	243	257	222	224	141	145	173	175
NYCM40	230	230	185	185	208	212	158	174	128	132	162	186	189	193	190	194	243	257	218	220	139	145	169	171
NYCM41	230	232	185	187	206	208	162	182	132	132	162	184	191	193	162	194	231	257	218	220	143	145	169	171
NYCM42	230	230	185	189	208	212	160	172	126	126	132	132	191	193	198	202	231	257	218	220	139	139	169	171
NYCM43	230	232	183	187	206	224	158	176	128	132	202	204	189	193	198	198	239	257	216	220	143	139	167	171
NYCM44	230	232	185	187	208	220	160	172	132	132	182	182	191	191	190	190	239	259	216	216	139	145	167	167

NYCM45	230	230	185	187	206	214	160	162	130	132	162	162	191	191	188	196	233	243	216	216	139	145	167	167
NYCM46	230	230	185	187	192	220	158	162	132	154	182	182	195	197	188	196	239	259	216	216	143	145	167	167
NYCM47	230	230	185	185	208	220	160	162	128	128	162	194	195	195	194	194	239	263	214	214	141	145	165	165
NYCM48	230	230	185	185	172	172	158	162	126	130	184	184	195	195	186	186	243	257	216	216	141	137	167	167
NYCM49	230	230	181	183	206	212	172	172	136	138	180	180	193	193	186	186	243	257	214	214	139	145	165	165
NYCM50	230	230	179	185	206	214	158	174	132	134	178	192	193	193	184	184	239	249	212	212	141	143	163	163
NHDF01	228	228	183	187	204	210	166	168	138	142	166	168	193	201	186	188	257	257	214	218	141	143	165	169
NHDF02	230	230	185	185	190	208	166	166	142	144	168	168	191	193	186	194	259	259	214	218	139	145	165	169
NHDF03	228	230	175	185	188	200	166	166	138	156	166	168	193	203	186	200	255	255	214	218	139	145	165	169
NHDF04	230	230	185	187	202	208	166	168	160	160	166	168	193	195	188	192	255	261	212	216	137	143	163	167
NHDF05	230	230	183	183	200	208	166	168	130	144	168	168	193	195	190	194	255	267	212	216	141	145	163	167
NHDF06	230	230	183	185	198	206	166	166	146	148	166	168	193	193	194	196	255	259	214	216	137	145	165	167
NHDF07	230	230	185	187	202	208	164	166	156	158	166	166	193	195	190	196	257	263	212	220	141	145	163	171
NHDF08	228	230	183	191	200	210	166	166	154	154	166	168	193	195	188	190	257	257	214	216	141	145	165	167
NHDF09	230	236	183	183	204	206	166	166	154	154	166	168	193	195	188	196	255	257	216	218	141	145	167	169
NHDF10	230	236	183	191	200	204	166	166	158	160	166	168	193	195	188	188	255	259	212	214	137	141	163	165
NHDF11	230	230	185	189	202	204	164	166	132	136	168	168	193	195	186	188	255	259	216	218	139	145	167	169
NHDF12	230	236	185	187	202	204	164	166	154	156	166	166	193	193	188	204	255	257	212	218	141	145	163	169
NHDF13	230	230	185	193	200	200	164	166	138	138	164	168	193	195	192	196	253	261	214	218	139	145	165	169
NHDF14	234	238	185	185	202	204	166	166	138	142	168	168	193	193	192	192	257	273	216	218	141	141	167	169
NHDF15	228	230	175	187	204	204	168	170	120	160	166	170	193	195	188	202	255	259	214	216	141	143	165	167
NHDF16	230	236	183	185	198	202	170	174	138	138	160	166	193	195	186	188	257	273	214	216	141	145	165	167
NHDF17	230	236	175	187	172	206	166	168	138	156	168	168	193	193	186	196	255	257	214	216	137	139	165	167
NHDF18	230	230	173	185	188	202	166	168	120	138	166	166	193	193	190	192	255	255	214	220	139	145	165	171
NHDF19	230	230	185	187	206	210	164	166	138	156	166	166	193	195	188	200	255	255	212	220	141	145	163	171
NHDF20	230	230	183	187	196	208	168	174	152	154	166	168	193	195	188	188	255	255	214	220	141	145	165	171
NHDF21	230	230	185	187	206	210	166	168	154	156	162	166	195	197	188	194	255	255	214	218	139	145	165	169
NHDF22	228	230	183	185	198	212	166	168	156	158	166	168	193	195	196	196	255	257	214	218	137	139	165	169
NHDF23	230	230	183	187	200	212	168	170	136	156	166	166	195	195	188	206	255	255	216	220	141	145	167	171
NHDF24	230	230	183	187	180	206	168	170	142	150	166	166	193	195	194	196	253	253	214	220	139	139	165	171
NHDF25	228	230	183	185	190	204	168	170	136	136	164	164	193	195	184	186	255	259	214	218	141	145	165	169
NHDF26	230	230	183	183	188	202	168	170	136	156	166	166	193	193	184	188	257	259	214	218	139	141	165	169
NHDF27	230	230	169	183	190	202	166	168	154	154	168	170	193	193	190	196	257	257	214	220	141	145	165	171
NHDF28	230	230	183	185	190	190	164	166	138	142	168	168	193	193	196	198	257	259	216	220	141	145	167	171
NHDF29	230	230	183	191	192	196	166	166	138	154	168	170	193	197	190	196	259	259	214	218	141	145	165	169
NHDF30	232	232	185	187	176	188	166	174	154	156	166	166	193	193	190	192	259	259	214	216	139	145	165	167
NHDF31	230	232	185	187	188	190	164	166	156	156	166	166	193	197	186	196	259	259	214	218	137	137	165	169
NHDF32	214	214	183	185	188	204	166	168	138	156	168	170	193	193	194	194	259	259	214	216	143	145	165	167
NHDF33	218	230	183	185	196	204	166	166	138	142	168	168	195	195	186	186	259	259	216	216	139	145	167	167
NHDF34	224	230	181	183	198	202	166	166	126	126	168	170	197	197	186	194	259	259	214	216	141	145	165	167
NHDF35	214	214	185	185	188	202	166	168	138	138	168	170	195	197	190	204	253	253	212	216	139	145	163	167
NHDF36	246	246	185	187	188	206	166	168	154	158	168	168	197	197	180	196	253	253	214	216	137	139	165	167
NHDF37	232	232	183	187	184	192	166	170	156	156	168	170	195	195	186	196	253	253	212	216	143	145	163	167
NHDF38	232	232	185	185	188	206	166	168	134	154	170	170	195	197	186	190	253	253	212	218	141	145	163	169
NHDF39	232	238	183	185	184	190	166	168	152	156	170	170	195	197	184	190	253	271	212	222	141	145	163	173
NHDF40	220	232	183	183	172	202	164	168	138	156	170	170	195	195	190	194	255	269	214	218	141	145	165	169
NHDF41	232	232	183	185	202	208	166	170	138	156	170	170	193	195	190	196	259	259	208	220	139	145	159	171
NHDF42	232	232	183	185	188	200	166	168	156	156	166	170	195	195	194	196	259	259	214	216	139	139	165	167
NHDF43	222	222	185	187	204	206	166	168	158	160	168	168	195	197	188	196	261	261	214	214	143	139	165	165
NHDF44	234	240	181	185	186	188	166	170	158	160	168	168	197	197	186	190	261	263	212	216	139	145	163	167
NHDF45	232	240	183	185	196	200	166	168	120	136	168	168	195	197	188	190	261	261	212	216	139	145	163	167
NHDF46	232	232	183	183	198	200	166	170	138	138	168	168	195	197	192	202	261	261	216	216	143	143	167	167
NHDF47	232	238	183	185	198	206	152	168	136	156	168	168	195	195	184	188	261	261	214	216	141	145	165	167
NHDF48	226	232	185	185	198	208	160	166	154	156	168	170	195	195	186	186	259	261	214	216	141	141	165	167
NHDF49	230	232	183	185	196	208	166	168	158	158	160	170	193	193	184	188	259	259	212	218	139	145	163	169
NHDF50	230	230	183	185	196	206	168	168	138	146	160	162	193	193	188	194	255	255	218	218	139	145	169	169
MNWL01	200	228	195	197	200	200	148	162	126	130	164	166	191	191	196	198	255	259	200	214	147	151	151	165
MNWL02	228	228	195	195	200	202	148	148	126	128	164	166	195	201	196	198	255	261	214	216	143	147	165	167
MNWL03	228	228	193	195	210	210	148	164	128	130	166	166	193	193	194	196	257	259	214	214	149	149	165	165
MNWL04	228	230	189	195	200	200	146	156	126	128	162	164	191	191	196	196	257	259	214	214	149	153	165	165
MNWL05	228	230	193	197	198	198	146	146	126	130	164	166	193	193	198	202	259	261	214	214	147	151	165	165
MNWL06	226	228	191	197	200	204	138	138	128	132	130	168	193	193	196	204	259	261	214	214	143	145	165	165
MNWL07	228	242	195	199	200	200	148	162	124	128	164	166	191	191	194	198	257	275	214	214	147	149	165	165
MNWL08	200	228	193	197	200	204	148	148	128	130	164	166	193	209	166	192	263	265	214	216	145	149	165	167
MNWL09	228	228	193	197	198	202	148	164	124	142	166	166	193	193	194	194	259	271	212	216	145	149	163	167
MNWL10	228	228	191	195	198	202	146	148	128	130	166	168	191	191	194	204	259	279	214	216	147	149	165	167
MNWL11	228	230	189	193	200	204	164	168	130	132	166	166	191	201	196	198	259	263	200	216	141	145	151	167
MNWL12	228	228	189	195	202	206	150	176	128	130	162	164	191	193	194	200	263	263	214	216	149	149	165	167
MNWL13	228	228	185	197	200	206	146	150	122	124	166	192	191	191	196	198	259	263	214	216	147	153	165	167
MNWL14	228	228	193	197	202	206	152	154	122	124	166	192	193	201	198	200	259	279	214	216	147	151	165	167
MNWL15	228	228	193	197	210	210	162	162	128	130	166	168	193	193	198	200	263	265	216	218	149	151	167	169
MNWL16	226	226	193	195	200	200	148	164	126	128	166	168	191	191	196	202	261	267	216	218	147	153	167	169
MNWL17	226	226	195	195	198	198	146	166	126	130	166	166	189	189	196	200	267	269	214	218	143	145	165	169
MNWL18	224	226	193	195	188	204	152	158	124	128	166	168	191	193	198	202	265	269	200	220	147	149	151	171
MNWL19	226	226	189	197	202	208	150	164	126	128	166	168	191	191	196	204	259	259	218	222	149	151	169	173
MNWL20	226	228	193	193	198	204	146	164	126	136	166	166	193	193	194	198	261	277	214	218	147	153	165	169
MNWL21	226	226	193	197	200	200	146	164	124	124	166	166	193	193	166	192	257	275	214	218	147	149	165	169
MNWL22	224	226	197	197	198	202	148	164	124	128	166	166	191	191	192	194	263	265	214	220	143	149	165	171
MNWL23	224	226	193	193	204	206	148	148	126	128	166	166	189	189	196	198	259	271	214	218	149	151	165	169
MNWL24	226	226	193	197	200	204	154	166	122	126	164	166	181	191	194	198	265	269	216	218	147	151	167	169
MNWL25	226	226	191	197	188	204	144	148	126	128	166	166	189	193	196	200	265	267	214	216	149	151	165	167
MNWL26	224	226	195	197	202	208	148	152	126	128	166	166	195	195	194	196	257	263	214	216	143	145	165	167
MNWL27	226	226	195	203	198	204	146	148	118	126	166	168	193	201	194	202	259	265	200	216	149	149	151	167
MNWL28	226	226	195	201	200	200	168	170	126	126	168	168	193	193	196	198	259	263	214	218	147	149	165	169
MNWL29	228	232	195	197	202	202	152	158	126	130	166	168	191	195	194	196	259	263	214	216	147	153	165	167
MNWL30	228	234	195	197	196	198	150	158	128	128	164	166	191	191	194	198	257	273	214	216	145	151	165	167
MNWL31	228	228	181	195	176	186	148	162	126	128	164	166	191	193	168	194	257	267	214	218	145	149	165	169
MNWL32	224	228	191	195	184	200	150	160	124	128	144	166	189	191	192	196	265	269	214	216	145	145	165	167
MNWL33	224	228	191	193	198	198	148	166	130	130	130	166	191	193	192	194	267	271	216	218	145	149	167	169
MNWL34	226	228	181	195	198	202	146	166	130	130	166	166	191	193	192	194	265	269	200	218	147	149	151	169
MNWL35	224	226	193	197	198	198	152	158	126	128	140	166	191	191	192	196	255	257	214	216	149	151	165	167
MNWL36	224	226	197	197	200	202	150	164	104	124	166	168	193	201	192	196	257	261	214	218	143	145	165	169
MNWL37	226	228	193	193	202	206	146	162	124	124	168	168	193	193	196	198	257	263	214	218	145	151	165	169
MNWL38	222	228	191	193	190	204	146	162	126	142	166	168	181	195	194	196	255	259	214	216	149	151	165	167
MNWL39	228	228	181	193	190	204	148	164	124	128	130	166	191	193	194	198	259	265	214	216	147	151	165	167
MNWL40	224	226	193	195	182	204	160	160	126	126	164	166	193	195	192	198	255	265	212	214	147	153	163	165
MNWL41	226	228	191	195	204	204	148	162	126	126	164	166	191	193	192	198	261	265	214	216	147	153	165	167
MNWL42	226	226	189	197	200	204	150	154	126	130	168	168	191	199	192	196	265	269	214	216	141	145	165	167
MNWL43	226	228	197	197	198	198	152	154	130	130	164	166	191	199	194	194	267	271	214	218	149	153	165	169
MNWL44	222	228	193	195	200	202	146	146	130	130	166	168	191	191	192	202	265	269	214	214	149	153	165	165
MNWL45	228	228	195	197	202	206	148	164	126	130	130	166	191	191	166	194	255	257	214	216	149	153	165	167
MNWL46	216	228	197	199	200	204	150	164	124	126	166	166	193	201	192	198	257	267	212	214	149	151	163	165
MNWL47	228	228	195	197	200	204	148	148	124	126	166	166	193	193	190	198	253	253	212	214	141	145	163	165
MNWL48	226	226	195	195	182	204	150	162	122	126	166	168	191	195	166	194	253	253	212	214	141	141	163	165
MNWL49	222	226	193	195	198	200	146	166	126	128	166	166	191	191	198	202	253	253	212	214	147	153	163	165
MNWL50	228	228	191	193	184	202	148	162	126	128	166	166	191	191	196	198	253	253	212	218	149	153	163	169
MNBL01	226	232	197	197	190	206	156	162	120	130	164	166	191	195	188	204	255	257	214	218	145	149	151	165
MNBL02	228	230	195	199	190	206	148	152	120	130	166	168	189	193	206	206	257	261	200	220	143	147	165	165
MNBL03	224	230	195	195	190	212	134	146	116	126	164	168	191	193	160	200	257	261	218	222	149	149	165	165
MNBL04	228	232	183	195	182	194	146	148	122	124	182	184	193	195	160	192	275	295	214	218	149	153	165	165
MNBL05	228	234	183	195	190	214	152	152	126	126	164	168	189	201	158	192	257	259	214	218	147	151	165	167
MNBL06	230	232	183	195	208	212	148	152	122	126	166	168	191	193	190	194	257	259	214	220	143	145	163	167
MNBL07	228	234	195	195	188	206	148	154	122	126	164	164	187	191	160	188	255	255	214	218	147	149	165	165
MNBL08	228	228	193	195	212	212	150	156	118	118	164	166	189	193	188	194	255	255	214	216	145	149	165	167
MNBL09	228	230	195	197	208	212	150	154	124	126	166	168	191	193	190	194	257	261	212	216	145	149	163	167
MNBL10	226	230	193	195	206	212	152	158	124	126	164	168	189	193	188	194	255	259	214	218	147	149	165	167
MNBL11	228	228	195	195	204	208	144	148	122	124	164	166	191	193	188	190	255	255	200	220	141	145	151	167
MNBL12	226	230	193	199	188	204	132	146	114	126	164	166	185	193	158	188	255	259	218	222	149	149	165	167
MNBL13	228	230	195	195	196	202	146	150	124	126	166	168	189	191	184	188	255	257	214	218	143	147	165	167
MNBL14	228	228	195	195	200	212	146	160	124	124	166	168	187	191	188	188	159	253	214	218	149	149	151	167
MNBL15	230	230	193	197	198	206	146	150	122	126	170	172	187	189	184	190	255	259	214	220	149	153	165	167
MNBL16	224	230	197	199	200	206	130	144	124	126	156	168	187	189	158	188	255	259	214	218	147	151	165	167
MNBL17	226	228	195	251	198	202	146	146	124	126	168	170	191	191	188	190	253	293	214	218	143	145	165	167

MNBL18	226	228	195	197	200	204	146	160	112	122	156	168	189	193	194	196	255	257	200	220	149	153	151	171
MNBL19	226	230	195	199	176	188	132	146	124	124	156	168	187	191	160	194	255	257	218	222	147	151	169	173
MNBL20	226	230	195	197	176	188	146	148	124	126	166	170	189	199	188	198	253	257	214	218	143	145	165	167
MNBL21	228	228	195	195	194	200	144	150	120	128	166	168	191	197	158	188	253	253	214	218	147	149	151	167
MNBL22	226	228	195	195	202	214	146	146	112	124	166	168	191	201	160	190	253	253	214	220	143	149	165	169
MNBL23	228	232	195	197	212	216	132	146	124	128	166	168	189	193	160	194	253	253	214	218	149	151	165	167
MNBL24	228	230	197	197	206	216	144	148	124	126	166	168	191	193	118	194	253	257	214	216	147	151	165	167
MNBL25	222	228	195	197	202	210	146	160	124	124	166	170	187	193	200	204	253	255	216	218	149	151	165	167
MNBL26	228	228	195	197	190	202	146	152	126	126	166	170	189	193	194	198	253	253	200	218	143	145	165	167
MNBL27	230	234	195	197	160	190	148	152	122	128	164	168	191	193	188	194	255	257	214	216	149	149	151	167
MNBL28	228	234	195	197	190	190	144	148	122	128	166	168	189	195	160	190	253	257	214	218	147	149	165	169
MNBL29	224	228	195	195	200	208	130	144	122	126	146	168	191	191	160	192	253	253	214	218	147	153	165	167
MNBL30	226	230	193	195	200	214	130	144	124	126	168	172	191	201	160	192	253	253	214	216	145	151	165	167
MNBL31	228	230	193	195	204	214	130	144	122	128	168	170	191	191	162	194	253	253	214	216	145	149	165	169
MNBL32	226	230	193	197	204	208	132	144	128	128	168	170	193	193	162	196	255	255	212	214	143	149	165	167
MNBL33	228	228	183	195	200	208	152	158	124	126	166	170	193	201	198	206	253	253	216	218	149	151	167	169
MNBL34	222	228	195	197	208	214	152	158	126	268	164	166	191	191	186	188	253	253	200	218	147	151	151	169
MNBL35	228	228	195	197	202	208	146	150	124	124	166	168	191	191	192	194	253	253	214	216	149	151	165	169
MNBL36	228	232	193	197	204	214	146	150	124	126	166	166	191	195	194	204	253	253	214	218	143	145	165	165
MNBL37	228	228	195	197	208	208	152	160	126	126	164	166	191	191	188	198	253	253	214	218	149	149	165	167
MNBL38	228	228	181	195	176	202	144	144	126	128	166	168	193	193	190	198	253	253	214	216	149	151	163	165
MNBL39	228	228	195	197	208	208	144	148	124	126	164	168	191	195	188	188	253	253	214	216	147	151	163	165
MNBL40	226	228	195	197	200	204	144	148	114	126	166	168	187	195	194	194	253	257	212	214	147	153	163	165
MNBL41	224	228	165	195	196	198	146	160	124	128	166	168	117	191	160	190	251	287	214	216	147	153	165	167
MNBL42	226	228	193	195	198	202	146	148	126	128	166	168	191	195	186	190	253	257	214	216	141	145	165	167
MNBL43	228	232	193	195	194	198	146	146	124	128	164	168	191	195	188	188	251	255	214	218	149	153	165	169
MNBL44	228	228	191	195	196	198	146	160	124	128	166	168	187	195	194	194	251	287	214	214	149	153	165	165
MNBL45	228	228	195	197	198	202	146	148	126	128	166	168	117	191	160	190	253	257	214	216	149	153	163	165
MNBL46	228	228	193	197	194	198	146	146	124	128	166	168	191	195	186	190	251	255	214	218	147	153	163	165
MNBL47	226	228	193	195	196	198	146	160	124	128	164	168	191	195	188	188	251	287	214	214	147	153	163	169
MNBL48	224	228	195	197	198	202	146	148	126	128	166	168	187	195	194	194	253	257	214	216	141	145	163	165
MNBL49	226	228	195	197	194	198	146	146	124	128	166	168	117	191	160	190	251	255	212	214	147	153	163	165
MNBL50	226	228	191	195	194	198	146	146	124	128	166	168	191	195	186	190	251	255	212	214	149	153	163	169
PAOL01	190	230	177	185	190	190	148	160	132	134	182	194	179	189	192	206	255	255	214	214	141	143	165	165
PAOL02	228	228	185	187	204	208	148	160	132	134	176	176	187	191	190	190	249	255	214	216	139	145	165	169
PAOL03	228	228	187	187	208	208	148	158	132	134	176	176	191	191	190	190	255	269	214	216	139	145	165	171
PAOL04	230	230	177	191	184	190	148	158	132	132	170	178	189	191	192	196	239	259	220	220	137	143	171	171
PAOL05	230	230	191	193	206	210	158	174	144	144	142	166	191	193	184	202	239	245	214	216	141	145	165	173
PAOL06	230	230	185	191	206	206	158	176	138	144	184	184	191	191	194	194	239	245	216	216	137	145	167	167
PAOL07	228	228	175	185	172	208	158	194	122	138	160	184	171	193	164	194	237	251	216	216	141	143	167	169
PAOL08	228	230	185	185	210	210	160	182	134	134	162	182	191	193	194	196	241	253	218	220	143	145	169	171
PAOL09	228	230	185	207	204	208	146	158	126	128	186	196	195	195	192	204	255	259	218	220	143	143	169	171
PAOL10	230	230	185	205	182	186	158	172	128	128	154	186	191	195	202	202	255	261	218	220	143	143	169	171
PAOL11	230	230	187	193	208	212	158	178	132	132	188	188	193	201	196	198	253	257	218	220	139	145	169	175
PAOL12	230	232	173	187	210	214	146	156	132	132	160	186	181	191	166	192	257	257	218	224	141	145	167	167
PAOL13	230	230	187	187	208	208	162	180	130	132	184	188	189	191	166	202	257	257	220	220	139	145	171	171
PAOL14	230	236	187	189	192	210	160	160	130	132	180	186	189	191	190	202	257	257	220	220	141	145	169	169
PAOL15	230	230	187	187	192	194	162	178	132	136	164	184	193	195	192	196	257	261	220	222	143	143	167	167
PAOL16	230	230	187	187	192	210	160	178	130	132	180	184	193	195	190	190	255	257	218	222	143	145	169	173
PAOL17	230	230	189	193	192	202	156	166	130	132	184	186	193	193	192	206	257	273	220	222	137	139	171	173
PAOL18	230	230	177	187	206	208	150	164	154	154	184	198	193	193	184	190	255	273	218	222	145	145	169	173
PAOL19	230	230	189	191	192	208	150	158	132	132	184	184	193	195	192	198	257	263	218	222	139	145	169	171
PAOL20	228	228	187	195	206	206	158	176	130	130	184	186	193	195	194	194	255	273	216	220	145	145	167	169
PAOL21	222	228	179	187	206	206	160	162	124	130	184	186	195	197	190	190	239	257	218	218	145	145	169	173
PAOL22	228	230	179	187	186	196	156	166	124	146	164	166	193	195	194	196	241	249	218	218	141	143	169	169
PAOL23	222	228	185	189	206	206	160	174	118	130	166	168	193	193	194	196	239	257	220	220	141	145	171	171
PAOL24	222	228	185	185	176	186	160	166	120	130	164	168	191	193	194	196	237	261	218	220	139	139	169	169
PAOL25	226	228	177	187	206	210	160	190	136	140	166	166	191	191	192	200	253	255	216	216	141	145	167	173
PAOL26	230	230	177	189	186	210	162	178	136	138	156	166	189	191	192	192	257	259	214	216	145	141	165	173
PAOL27	230	230	187	187	186	208	162	178	134	140	156	166	189	193	198	200	257	257	214	216	143	145	165	173
PAOL28	230	236	187	187	178	208	162	176	132	134	186	186	193	195	202	202	255	259	214	214	141	145	165	169
PAOL29	230	230	185	187	208	222	158	166	132	136	164	186	191	193	192	194	253	257	214	214	145	145	165	177
PAOL30	228	230	183	183	206	210	162	166	132	132	164	184	189	191	192	196	253	273	214	214	143	145	165	177
PAOL31	228	230	171	171	178	182	160	174	134	138	186	190	189	193	192	202	241	257	224	226	137	137	175	175
PAOL32	228	230	189	207	190	208	162	174	128	134	180	186	187	191	192	198	239	253	222	224	143	145	173	173
PAOL33	230	236	185	193	204	208	174	178	132	134	156	182	179	191	192	198	239	251	220	220	139	145	171	171
PAOL34	224	230	185	193	190	192	162	176	132	134	160	184	187	191	192	198	243	253	220	220	145	149	169	169
PAOL35	228	228	185	187	192	192	158	176	128	130	180	188	195	197	192	194	243	253	232	240	139	145	167	183
PAOL36	228	228	187	189	192	208	162	172	130	134	182	186	197	197	190	194	259	259	222	232	137	139	173	175
PAOL37	232	248	185	187	192	210	172	180	134	148	158	186	195	195	190	192	255	255	222	224	145	147	173	173
PAOL38	230	230	185	185	192	208	158	178	130	130	184	186	195	197	192	192	257	257	222	224	143	145	173	173
PAOL39	230	230	183	187	202	206	158	176	132	136	182	186	189	193	190	204	255	255	222	224	141	145	173	173
PAOL40	230	230	185	185	202	206	158	172	130	132	168	184	189	193	190	194	253	257	216	216	139	145	167	171
PAOL41	230	232	185	185	190	208	146	158	132	132	142	164	191	193	162	194	255	271	214	216	143	145	165	171
PAOL42	230	230	183	185	178	208	156	174	126	126	166	166	191	193	198	202	243	257	214	216	139	139	165	171
PAOL43	230	232	183	201	206	224	156	170	128	132	166	166	189	193	198	198	231	257	214	214	143	147	165	167
PAOL44	228	228	201	203	208	220	160	172	132	132	182	182	191	191	190	190	231	257	214	214	139	145	165	167
PAOL45	224	230	183	203	206	214	160	162	130	132	162	162	191	191	188	196	239	257	214	214	139	145	165	167
PAOL46	230	230	185	187	192	220	158	162	132	154	182	182	195	197	188	196	239	259	216	216	147	147	167	167
PAOL47	190	230	185	185	178	206	158	172	128	128	162	194	195	195	194	194	233	243	214	214	141	145	165	167
PAOL48	230	230	185	185	172	172	146	158	126	130	184	184	195	195	186	186	239	259	216	216	141	137	167	167
PAOL49	230	230	181	183	206	212	156	174	136	138	180	180	193	193	186	186	259	259	214	214	141	145	165	163
PAOL50	230	230	179	185	206	214	156	170	132	134	178	192	193	193	184	184	255	255	212	212	141	143	163	163
VABS01	226	226	183	185	196	206	160	174	132	134	178	178	193	193	186	190	253	267	212	212	137	143	165	165
VABS02	230	230	173	185	204	214	168	168	130	140	162	164	189	189	186	186	255	271	210	214	137	143	163	165
VABS03	232	236	181	187	212	212	168	172	134	144	164	164	189	189	192	194	259	265	210	210	141	141	161	161
VABS04	226	228	185	189	202	204	148	156	150	154	160	164	187	187	188	194	261	263	212	216	143	143	167	169
VABS05	228	228	181	197	204	208	156	156	154	154	162	182	187	189	186	190	253	253	212	214	135	141	165	169
VABS06	228	234	173	187	182	204	154	168	152	152	172	190	189	193	188	190	257	257	212	214	135	143	169	173
VABS07	228	228	183	185	194	194	156	156	120	124	160	164	193	195	192	198	257	259	212	212	137	141	163	167
VABS08	228	228	181	181	192	196	158	174	130	132	166	166	193	195	190	192	251	251	210	214	137	145	165	169
VABS09	230	236	183	191	204	210	154	174	124	124	168	186	191	191	194	194	253	253	212	214	137	143	163	167
VABS10	230	230	185	187	188	188	156	170	138	140	164	164	189	191	184	186	235	255	214	214	137	143	165	167
VABS11	230	230	179	179	186	188	166	170	114	146	180	184	185	193	194	194	251	251	210	210	137	143	163	163
VABS12	226	226	181	181	188	204	146	160	128	156	142	162	189	195	196	200	253	253	210	210	139	143	163	163
VABS13	224	224	183	187	204	210	164	168	120	124	162	166	187	193	190	192	253	271	214	216	141	141	163	163
VABS14	226	228	197	197	198	204	158	190	132	132	166	166	189	191	188	196	237	249	214	214	137	143	163	165
VABS15	232	232	183	187	200	200	166	168	126	130	180	180	187	187	190	202	257	257	214	216	141	145	165	167
VABS16	230	230	185	185	186	204	158	166	128	156	166	166	191	193	198	206	257	269	210	212	141	147	163	167
VABS17	224	224	169	183	178	202	160	176	124	126	160	164	191	193	186	192	255	255	214	214	139	143	165	167
VABS18	228	228	191	195	206	206	158	172	122	126	162	166	191	193	188	190	259	259	212	216	137	143	165	165
VABS19	232	232	185	185	214	214	168	168	146	152	162	162	189	189	190	190	251	251	212	214	141	141	165	165
VABS20	222	226	181	181	204	206	170	170	128	138	162	166	187	191	192	198	251	251	208	214	139	143	165	169
VABS21	228	232	185	197	212	214	158	172	128	130	164	166	189	191	186	198	253	253	212	212	143	145	161	165
VABS22	226	226	183	185	190	194	152	156	130	144	182	182	191	191	190	194	261	265	212	212	139	145	163	163
VABS23	226	226	177	183	198	204	166	168	128	130	180	192	189	189	190	192	255	259	212	212	139	139	165	165
VABS24	228	228	181	187	196	208	164	166	126	144	164	164	185	193	190	190	253	269	212	212	137	143	161	161
VABS25	226	226	179	179	194	194	152	152	126	128	172	192	191	193	188	190	253	253	212	216	141	147	161	163
VABS26	218	224	183	185	188	202	146	158	128	128	142	164	189	189	184	188	253	253	212	212	137	143	165	165
VABS27	224	230	185	185	202	202	148	172	126	130	164	166	189	191	190	200	253	253	212	214	139	145	165	165
VABS28	226	228	185	195	176	202	156	156	128	128	168	186	193	193	188	196	253	253	210	214	137	143	165	167
VABS29	228	228	181	185	176	208	156	158	130	130	162	164	191	193	184	186	253	257	212	212	137	143	163	167
VABS30	226	228	181	185	176	202	156	172	130	132	164	164	187	191	190	192	253	253	212	212	137	143	165	165
VABS31	230	230	185	185	200	206	144	156	136	136	142	166	189	191	186	194	255	257	202	214	139	143	165	165
VABS32	212	212	169	181	192	206	156	156	120	124	164	164	185	189	192	196	253	253	212	212	137	143	161	163
VABS33	226	228	183	185	206	206	156	172	130	134	164	164	193	193	186	192	259	259	212	216	137	143	167	169
VABS34	226	226	189	191	200	202	156	166	130	132	166	182	187	189	188	188	253	253	212	214	141	145	165	167
VABS35	230	230	183	189	206	208	166	170	134	134	164	164	189	191	184	188	251	251	216	216	137	143	167	169
VABS36	226	228	183	187	196	206	158	170	122	124	162	164	189	189	192	192	249	253	214	214	137	143	165	167
VABS37	224	230	181	183	170	200	164	164	126	134	164	166	189	189	158	184	253	253	210	212	143	143	163	163
VABS38	228	228	183	185	202	206	150	156	130	132	166	168	189	189	190	192	241	251	210	214	137	141	165	167
VABS39	228	230	181	187	188	204	156	190	132	136	162	166	193	195	188	198	239	249	210	212	143	145	161	161
VABS40	226	226	169	185	204	204	168	168	130	138	168	168	191	193	192	196	253	259	212	212	137	143	163	165

VABS41	228	228	185	187	172	202	158	164	126	132	164	164	191	193	190	198	239	255	212	212	137	143	163	167
VABS42	228	228	181	181	206	206	146	158	124	126	160	164	177	187	158	186	253	253	216	216	139	143	171	173
VABS43	226	232	179	183	180	200	154	172	130	134	138	164	193	193	192	192	253	253	208	212	137	143	165	169
VABS44	228	230	181	183	202	206	160	176	126	146	168	186	189	189	188	190	253	253	212	214	139	143	165	165
VABS45	220	220	183	185	180	180	162	190	124	128	164	164	187	191	186	188	261	263	212	214	139	143	165	169
VABS46	226	230	181	181	200	212	146	156	134	134	166	168	189	191	164	190	237	261	216	216	137	143	163	169
VABS47	228	230	185	191	188	192	170	170	130	134	162	162	187	187	166	192	257	257	214	214	135	143	163	167
VABS48	214	228	173	185	202	204	164	164	128	132	142	162	191	191	190	198	257	259	212	214	135	139	165	165
VABS49	226	226	183	183	190	210	156	156	130	132	164	166	191	193	190	192	253	259	216	218	147	149	165	165
VABS50	226	226	179	179	198	202	158	172	134	134	154	164	191	193	184	188	253	267	216	216	141	141	163	167
NCAV01	234	234	187	189	196	210	168	202	128	132	168	168	195	195	196	204	257	257	194	218	141	149	151	167
NCAV02	234	236	185	197	208	212	150	160	134	136	170	172	191	191	196	196	257	273	218	218	141	147	169	169
NCAV03	234	236	185	197	200	200	172	172	132	138	168	172	193	193	196	202	255	257	216	216	141	147	169	171
NCAV04	216	216	185	197	190	202	150	166	132	132	168	170	195	195	192	202	255	255	216	216	151	153	167	167
NCAV05	240	240	189	191	186	192	150	162	128	132	168	168	191	193	190	192	257	271	218	218	141	147	167	171
NCAV06	246	246	185	185	210	214	168	170	134	136	192	194	195	195	196	200	271	275	216	216	141	149	167	167
NCAV07	234	238	197	197	200	204	148	176	130	150	144	168	191	191	200	206	261	261	0	0	141	147	169	171
NCAV08	232	238	195	197	192	202	160	176	134	136	166	166	193	203	188	202	255	257	214	216	141	147	151	167
NCAV09	232	232	197	199	190	222	146	158	134	134	158	168	199	207	194	202	257	263	216	216	143	147	169	171
NCAV10	232	232	185	191	198	200	162	162	126	130	164	166	195	195	202	204	257	257	214	218	141	147	159	169
NCAV11	230	230	177	187	206	206	162	176	132	132	178	180	197	199	166	166	257	257	216	218	139	139	165	165
NCAV12	230	236	199	199	192	204	150	166	130	132	168	168	195	203	190	196	255	255	214	214	141	147	169	173
NCAV13	230	230	197	201	196	208	160	176	130	132	166	168	193	193	166	192	241	263	216	218	139	147	167	167
NCAV14	230	230	197	197	208	214	148	168	134	134	164	170	193	193	190	200	259	259	216	216	141	147	169	171
NCAV15	228	228	171	181	188	212	160	162	134	134	168	168	189	195	198	200	263	263	212	216	145	145	167	169
NCAV16	228	228	171	191	188	210	162	162	132	132	166	170	179	193	196	198	259	259	214	216	145	147	167	167
NCAV17	230	236	185	191	190	212	160	162	124	124	172	176	189	195	190	192	257	259	216	218	141	147	169	169
NCAV18	230	230	185	185	206	208	162	178	140	140	164	186	191	193	198	200	257	257	214	216	143	147	169	171
NCAV19	228	228	185	187	206	206	162	176	128	132	168	168	197	197	192	194	255	255	216	216	137	147	167	167
NCAV20	228	228	185	189	194	214	160	178	124	124	162	166	195	195	190	200	257	257	216	216	145	147	165	167
NCAV21	228	228	185	189	198	202	164	164	130	136	160	168	197	199	194	196	255	271	216	218	143	145	167	167
NCAV22	230	230	173	185	208	208	164	180	124	126	168	168	191	195	192	192	259	259	216	220	145	147	167	169
NCAV23	230	230	175	185	208	208	176	176	124	128	190	194	209	209	190	190	253	253	216	216	141	147	169	171
NCAV24	230	230	185	185	188	198	144	152	132	160	168	172	195	195	194	200	265	267	218	222	141	147	169	169
NCAV25	230	230	185	185	190	208	168	170	126	130	168	168	119	193	188	190	245	263	216	218	143	147	167	167
NCAV26	234	234	185	201	186	194	160	180	120	132	154	158	191	191	196	200	257	257	216	216	141	147	167	169
NCAV27	230	234	185	203	208	212	158	158	132	136	174	192	191	197	164	194	259	263	216	218	141	147	167	169
NCAV28	230	230	181	183	208	214	184	192	134	134	190	190	193	195	188	190	253	255	218	218	145	145	167	167
NCAV29	228	232	173	185	194	206	160	184	142	142	168	170	193	193	196	196	259	259	216	218	141	147	171	175
NCAV30	234	234	185	185	212	214	170	172	130	132	166	168	193	203	192	196	261	281	216	218	145	147	169	169
NCAV31	230	230	185	185	212	212	162	174	130	132	146	170	193	197	198	204	255	255	200	216	141	147	167	171
NCAV32	230	238	183	183	212	212	158	160	140	158	166	166	193	195	190	192	261	265	216	218	141	147	167	169
NCAV33	232	232	187	189	210	212	158	176	140	144	168	168	179	193	192	196	257	261	214	220	145	149	169	171
NCAV34	232	234	177	189	210	212	160	174	124	128	170	172	189	191	194	198	251	259	214	224	145	151	153	171
NCAV35	232	236	177	191	192	192	160	174	130	132	168	170	191	193	174	188	257	259	216	216	145	145	167	169
NCAV36	234	236	189	191	180	210	160	166	132	134	166	168	197	197	196	196	255	255	218	218	141	147	163	169
NCAV37	234	234	187	189	210	224	170	174	130	134	168	194	191	191	202	208	257	261	220	222	141	147	171	173
NCAV38	232	232	187	195	208	212	162	162	128	132	168	170	193	193	200	200	257	275	216	218	147	147	169	169
NCAV39	232	232	185	203	180	184	168	174	132	134	168	170	195	195	202	206	259	259	216	216	149	151	167	167
NCAV40	230	232	185	203	190	190	174	182	130	132	166	168	195	205	186	204	257	259	216	218	141	147	167	171
NCAV41	232	232	187	203	212	212	164	178	132	134	170	172	193	205	194	198	255	255	216	216	145	151	171	171
NCAV42	230	230	185	185	176	176	168	168	132	136	158	168	191	191	204	204	257	257	216	220	143	143	169	171
NCAV43	230	230	181	183	172	174	170	172	126	130	168	172	193	195	194	200	257	257	214	216	145	149	165	165
NCAV44	224	230	173	185	190	212	162	194	126	126	168	168	189	201	192	194	249	257	216	216	143	147	169	171
NCAV45	230	230	185	185	208	210	158	162	132	138	168	168	193	195	192	196	255	255	216	218	139	139	165	167
NCAV46	230	230	185	187	190	212	170	170	134	138	164	196	193	203	188	192	255	255	216	218	141	147	169	169
NCAV47	230	230	183	189	212	216	176	176	126	128	170	188	193	193	192	192	253	257	218	220	145	145	169	171
NCAV48	230	230	185	203	210	216	154	154	130	132	176	178	191	191	196	198	255	255	216	216	141	147	169	169
NCAV49	228	228	187	203	204	208	142	148	132	132	166	170	201	203	192	192	257	257	220	222	151	155	167	169
NCAV50	228	228	185	185	178	208	142	148	132	132	166	170	201	203	192	192	257	257	220	222	151	155	167	169
