# Supplementary material for: Post-glacial phylogeography and evolution of a wide-ranging highly-exploited keystone forest tree, eastern white pine (Pinus strobus) in North America: single refugium, multiple routes
Source: BMC Evol Biol. 2016 Mar 2;16:56. doi: 10.1186/s12862-016-0624-1 (PMC4774161; doi:10.1186/s12862-016-0624-1)
Supplement: Additional file 7: Table S4. — Chloroplast microsatellite genotype data. (PDF 138 kb) [file 12862_2016_624_MOESM7_ESM.pdf]

Post-glacial phylogeography and evolution of a wide-ranging highly-exploited keystone forest tree, eastern white pine (*Pinus strobus*) in North America: Single refugium, multiple routes

**John W. R. Zinck and Om P. Rajora**

**Table S4. Chloroplast microsatellite genotype data**

| Sample | pt26081 | pt63718 | pt71936 |
|---|---|---|---|
| NFGL01 | 136 | 115 | 163 |
| NFGL02 | 138 | 115 | 163 |
| NFGL03 | 136 | 115 | 163 |
| NFGL04 | 136 | 114 | 163 |
| NFGL05 | 126 | 115 | 163 |
| NFGL06 | 126 | 115 | 163 |
| NFGL07 | 136 | 114 | 163 |
| NFGL08 | 136 | 113 | 163 |
| NFGL09 | 132 | 113 | 163 |
| NFGL10 | 136 | 115 | 163 |
| NFGL11 | 136 | 115 | 163 |
| NFGL12 | 136 | 114 | 163 |
| NFGL13 | 136 | 115 | 163 |
| NFGL14 | 136 | 114 | 163 |
| NFGL15 | 136 | 115 | 163 |
| NFGL16 | 134 | 114 | 163 |
| NFGL17 | 134 | 115 | 163 |
| NFGL18 | 136 | 115 | 163 |
| NFGL19 | 136 | 114 | 163 |
| NFGL20 | 136 | 115 | 163 |
| NBPMH01 | 136 | 113 | 163 |
| NBPMH02 | 136 | 113 | 163 |
| NBPMH03 | 136 | 115 | 161 |
| NBPMH04 | 126 | 115 | 161 |
| NBPMH05 | 136 | 115 | 163 |
| NBPMH06 | 136 | 115 | 163 |
| NBPMH07 | 136 | 115 | 163 |
| NBPMH08 | 136 | 114 | 161 |
| NBPMH09 | 136 | 115 | 161 |
| NBPMH10 | 136 | 115 | 163 |
| NBPMH11 | 126 | 113 | 161 |
| NBPMH12 | 136 | 115 | 161 |
| NBPMH13 | 136 | 115 | 163 |
| NBPMH14 | 136 | 115 | 163 |
| NBPMH15 | 136 | 113 | 163 |
| NBPMH16 | 136 | 115 | 163 |
| NBPMH17 | 136 | 115 | 163 |
| NBPMH18 | 136 | 114 | 163 |
| NBPMH19 | 136 | 115 | 163 |
| NBPMH20 | 136 | 114 | 163 |
| NBCI01 | 136 | 115 | 163 |

| | | | |
|---|---|---|---|
| NBCI02 | 136 | 113 | 163 |
| NBCI03 | 136 | 115 | 163 |
| NBCI04 | 136 | 113 | 163 |
| NBCI05 | 126 | 115 | 163 |
| NBCI06 | 136 | 115 | 163 |
| NBCI07 | 136 | 114 | 163 |
| NBCI08 | 126 | 115 | 163 |
| NBCI09 | 136 | 115 | 161 |
| NBCI10 | 136 | 115 | 163 |
| NBCI11 | 126 | 115 | 161 |
| NBCI12 | 136 | 115 | 163 |
| NBCI13 | 136 | 113 | 161 |
| NBCI14 | 136 | 113 | 163 |
| NBCI15 | 136 | 115 | 163 |
| NBCI16 | 136 | 115 | 163 |
| NBCI17 | 136 | 114 | 163 |
| NBCI18 | 136 | 115 | 163 |
| NBCI19 | 136 | 115 | 163 |
| NBCI20 | 136 | 115 | 163 |
| NBCR01 | 136 | 114 | 163 |
| NBCR02 | 136 | 114 | 163 |
| NBCR03 | 136 | 115 | 163 |
| NBCR04 | 126 | 115 | 163 |
| NBCR05 | 136 | 115 | 163 |
| NBCR06 | 136 | 113 | 163 |
| NBCR07 | 136 | 115 | 161 |
| NBCR08 | 136 | 113 | 163 |
| NBCR09 | 136 | 115 | 163 |
| NBCR10 | 126 | 115 | 163 |
| NBCR11 | 136 | 115 | 163 |
| NBCR12 | 136 | 115 | 163 |
| NBCR13 | 136 | 115 | 163 |
| NBCR14 | 126 | 115 | 163 |
| NBCR15 | 136 | 115 | 163 |
| NBCR16 | 136 | 113 | 163 |
| NBCR17 | 126 | 115 | 163 |
| NBCR18 | 136 | 113 | 163 |
| NBCR19 | 126 | 115 | 163 |
| NBCR20 | 136 | 114 | 163 |
| NBOP01 | 136 | 115 | 163 |
| NBOP02 | 136 | 115 | 163 |
| NBOP03 | 126 | 115 | 163 |
| NBOP04 | 136 | 115 | 163 |
| NBOP05 | 136 | 115 | 161 |
| NBOP06 | 136 | 115 | 163 |
| NBOP07 | 136 | 113 | 163 |
| NBOP08 | 136 | 115 | 163 |
| NBOP09 | 126 | 115 | 163 |
| NBOP10 | 136 | 115 | 163 |

| | | | |
|---|---|---|---|
| NBOP11 | 136 | 114 | 161 |
| NBOP12 | 136 | 115 | 161 |
| NBOP13 | 128 | 115 | 163 |
| NBOP14 | 136 | 115 | 163 |
| NBOP15 | 136 | 115 | 163 |
| NBOP16 | 136 | 113 | 163 |
| NBOP17 | 136 | 113 | 163 |
| NBOP18 | 136 | 113 | 163 |
| NBOP19 | 136 | 115 | 163 |
| NBOP20 | 136 | 114 | 163 |
| NSSMB01 | 136 | 113 | 163 |
| NSSMB02 | 126 | 115 | 163 |
| NSSMB03 | 136 | 113 | 163 |
| NSSMB04 | 136 | 114 | 165 |
| NSSMB05 | 136 | 115 | 163 |
| NSSMB06 | 136 | 115 | 163 |
| NSSMB07 | 136 | 115 | 161 |
| NSSMB08 | 136 | 115 | 165 |
| NSSMB09 | 136 | 114 | 163 |
| NSSMB10 | 136 | 115 | 163 |
| NSSMB11 | 136 | 115 | 161 |
| NSSMB12 | 136 | 115 | 163 |
| NSSMB13 | 136 | 113 | 163 |
| NSSMB14 | 136 | 115 | 163 |
| NSSMB15 | 136 | 115 | 163 |
| NSSMB16 | 136 | 115 | 165 |
| NSSMB17 | 136 | 114 | 163 |
| NSSMB18 | 136 | 115 | 163 |
| NSSMB19 | 136 | 115 | 163 |
| NSSMB20 | 136 | 115 | 163 |
| NSRL01 | 136 | 115 | 165 |
| NSRL02 | 136 | 113 | 163 |
| NSRL03 | 136 | 113 | 163 |
| NSRL04 | 136 | 115 | 163 |
| NSRL05 | 136 | 113 | 163 |
| NSRL06 | 136 | 114 | 161 |
| NSRL07 | 136 | 114 | 163 |
| NSRL08 | 126 | 115 | 165 |
| NSRL09 | 136 | 113 | 165 |
| NSRL10 | 136 | 115 | 163 |
| NSRL11 | 136 | 114 | 163 |
| NSRL12 | 136 | 115 | 163 |
| NSRL13 | 138 | 113 | 163 |
| NSRL14 | 136 | 115 | 163 |
| NSRL15 | 136 | 115 | 163 |
| NSRL16 | 136 | 115 | 163 |
| NSRL17 | 136 | 115 | 163 |
| NSRL18 | 136 | 115 | 163 |
| NSRL19 | 136 | 113 | 163 |

| | | | |
|---|---|---|---|
| NSRL20 | 136 | 115 | 163 |
| NSDL01 | 136 | 115 | 163 |
| NSDL02 | 136 | 115 | 163 |
| NSDL03 | 136 | 113 | 163 |
| NSDL04 | 136 | 115 | 161 |
| NSDL05 | 136 | 114 | 163 |
| NSDL06 | 136 | 114 | 163 |
| NSDL07 | 136 | 114 | 163 |
| NSDL08 | 136 | 115 | 165 |
| NSDL09 | 126 | 115 | 165 |
| NSDL10 | 136 | 115 | 163 |
| NSDL11 | 136 | 113 | 163 |
| NSDL12 | 136 | 115 | 161 |
| NSDL13 | 136 | 114 | 163 |
| NSDL14 | 136 | 114 | 163 |
| NSDL15 | 126 | 115 | 163 |
| NSDL16 | 136 | 113 | 163 |
| NSDL17 | 136 | 113 | 163 |
| NSDL18 | 136 | 115 | 165 |
| NSDL19 | 136 | 115 | 163 |
| NSDL20 | 136 | 115 | 163 |
| NSUM01 | 136 | 115 | 163 |
| NSUM02 | 136 | 113 | 163 |
| NSUM03 | 136 | 115 | 165 |
| NSUM04 | 136 | 113 | 163 |
| NSUM05 | 136 | 115 | 163 |
| NSUM06 | 136 | 115 | 161 |
| NSUM07 | 136 | 114 | 163 |
| NSUM08 | 136 | 115 | 163 |
| NSUM09 | 136 | 114 | 165 |
| NSUM10 | 136 | 115 | 165 |
| NSUM11 | 136 | 113 | 163 |
| NSUM12 | 136 | 115 | 163 |
| NSUM13 | 126 | 115 | 163 |
| NSUM14 | 136 | 113 | 165 |
| NSUM15 | 136 | 114 | 163 |
| NSUM16 | 136 | 114 | 163 |
| NSUM17 | 136 | 115 | 163 |
| NSUM18 | 136 | 115 | 163 |
| NSUM19 | 136 | 115 | 163 |
| NSUM20 | 136 | 113 | 163 |
| PQTM01 | 136 | 115 | 163 |
| PQTM02 | 126 | 115 | 163 |
| PQTM03 | 136 | 115 | 161 |
| PQTM04 | 136 | 113 | 163 |
| PQTM05 | 136 | 115 | 163 |
| PQTM06 | 136 | 115 | 161 |
| PQTM07 | 136 | 114 | 163 |
| PQTM08 | 126 | 115 | 163 |

| | | | |
|---|---|---|---|
| PQTM09 | 136 | 115 | 161 |
| PQTM10 | 136 | 115 | 163 |
| PQTM11 | 136 | 113 | 163 |
| PQTM12 | 126 | 115 | 163 |
| PQTM13 | 136 | 115 | 165 |
| PQTM14 | 126 | 115 | 163 |
| PQTM15 | 136 | 114 | 163 |
| PQTM16 | 126 | 115 | 163 |
| PQTM17 | 136 | 115 | 165 |
| PQTM18 | 126 | 113 | 163 |
| PQTM19 | 136 | 114 | 163 |
| PQTM20 | 136 | 113 | 163 |
| PQCT01 | 136 | 115 | 161 |
| PQCT02 | 136 | 115 | 165 |
| PQCT03 | 136 | 114 | 163 |
| PQCT04 | 126 | 115 | 161 |
| PQCT05 | 126 | 114 | 165 |
| PQCT06 | 136 | 113 | 165 |
| PQCT07 | 136 | 115 | 161 |
| PQCT08 | 126 | 115 | 161 |
| PQCT09 | 136 | 114 | 163 |
| PQCT10 | 126 | 115 | 163 |
| PQCT11 | 136 | 115 | 161 |
| PQCT12 | 136 | 114 | 163 |
| PQCT13 | 126 | 115 | 163 |
| PQCT14 | 136 | 114 | 163 |
| PQCT15 | 136 | 113 | 163 |
| PQCT16 | 126 | 115 | 163 |
| PQCT17 | 126 | 115 | 163 |
| PQCT18 | 136 | 114 | 163 |
| PQCT19 | 136 | 115 | 163 |
| PQCT20 | 126 | 115 | 163 |
| PQSR01 | 136 | 114 | 163 |
| PQSR02 | 138 | 115 | 163 |
| PQSR03 | 136 | 113 | 163 |
| PQSR04 | 136 | 114 | 161 |
| PQSR05 | 136 | 115 | 163 |
| PQSR06 | 136 | 115 | 161 |
| PQSR07 | 126 | 115 | 163 |
| PQSR08 | 136 | 115 | 161 |
| PQSR09 | 136 | 115 | 163 |
| PQSR10 | 136 | 115 | 163 |
| PQSR11 | 126 | 115 | 165 |
| PQSR12 | 136 | 115 | 163 |
| PQSR13 | 136 | 115 | 163 |
| PQSR14 | 136 | 115 | 163 |
| PQSR15 | 136 | 115 | 165 |
| PQSR16 | 136 | 115 | 163 |
| PQSR17 | 136 | 115 | 163 |

| | | | |
|---|---|---|---|
| PQSR18 | 138 | 115 | 163 |
| PQSR19 | 136 | 115 | 163 |
| PQSR20 | 136 | 115 | 163 |
| PQSS01 | 126 | 115 | 163 |
| PQSS02 | 136 | 115 | 163 |
| PQSS03 | 136 | 115 | 161 |
| PQSS04 | 136 | 115 | 161 |
| PQSS05 | 126 | 115 | 163 |
| PQSS06 | 136 | 115 | 163 |
| PQSS07 | 136 | 115 | 163 |
| PQSS08 | 136 | 115 | 161 |
| PQSS09 | 126 | 115 | 163 |
| PQSS10 | 136 | 115 | 163 |
| PQSS11 | 136 | 115 | 165 |
| PQSS12 | 138 | 115 | 163 |
| PQSS13 | 136 | 115 | 163 |
| PQSS14 | 136 | 115 | 163 |
| PQSS15 | 126 | 115 | 163 |
| PQSS16 | 136 | 115 | 163 |
| PQSS17 | 136 | 115 | 163 |
| PQSS18 | 126 | 115 | 163 |
| PQSS19 | 126 | 115 | 163 |
| PQSS20 | 136 | 115 | 163 |
| PQLP01 | 136 | 115 | 163 |
| PQLP02 | 126 | 115 | 163 |
| PQLP03 | 136 | 114 | 163 |
| PQLP04 | 136 | 115 | 161 |
| PQLP05 | 136 | 115 | 163 |
| PQLP06 | 126 | 115 | 163 |
| PQLP07 | 136 | 114 | 161 |
| PQLP08 | 136 | 115 | 161 |
| PQLP09 | 136 | 115 | 161 |
| PQLP10 | 136 | 114 | 163 |
| PQLP11 | 136 | 115 | 163 |
| PQLP12 | 126 | 115 | 163 |
| PQLP13 | 136 | 115 | 163 |
| PQLP14 | 138 | 115 | 163 |
| PQLP15 | 136 | 115 | 163 |
| PQLP16 | 136 | 115 | 163 |
| PQLP17 | 126 | 115 | 163 |
| PQLP18 | 136 | 114 | 163 |
| PQLP19 | 138 | 115 | 163 |
| PQLP20 | 138 | 115 | 163 |
| ONML01 | 140 | 115 | 163 |
| ONML02 | 136 | 115 | 161 |
| ONML03 | 126 | 115 | 169 |
| ONML04 | 136 | 115 | 169 |
| ONML05 | 136 | 115 | 163 |
| ONML06 | 140 | 115 | 163 |

| | | | |
|---|---|---|---|
| ONML07 | 136 | 114 | 163 |
| ONML08 | 136 | 115 | 163 |
| ONML09 | 136 | 115 | 163 |
| ONML10 | 138 | 115 | 161 |
| ONML11 | 136 | 115 | 163 |
| ONML12 | 138 | 115 | 163 |
| ONML13 | 136 | 114 | 163 |
| ONML14 | 138 | 115 | 169 |
| ONML15 | 136 | 115 | 163 |
| ONML16 | 136 | 115 | 163 |
| ONML17 | 136 | 116 | 169 |
| ONML18 | 136 | 115 | 163 |
| ONML19 | 136 | 115 | 163 |
| ONML20 | 136 | 115 | 169 |
| ONFR01 | 138 | 115 | 163 |
| ONFR02 | 126 | 115 | 161 |
| ONFR03 | 138 | 114 | 163 |
| ONFR04 | 138 | 115 | 159 |
| ONFR05 | 126 | 115 | 163 |
| ONFR06 | 126 | 115 | 169 |
| ONFR07 | 126 | 115 | 163 |
| ONFR08 | 138 | 115 | 163 |
| ONFR09 | 136 | 114 | 163 |
| ONFR10 | 126 | 115 | 169 |
| ONFR11 | 136 | 115 | 161 |
| ONFR12 | 126 | 115 | 169 |
| ONFR13 | 126 | 115 | 163 |
| ONFR14 | 138 | 115 | 161 |
| ONFR15 | 136 | 115 | 163 |
| ONFR16 | 138 | 116 | 163 |
| ONFR17 | 136 | 115 | 161 |
| ONFR18 | 136 | 115 | 163 |
| ONFR19 | 138 | 115 | 163 |
| ONFR20 | 138 | 115 | 163 |
| ONHF01 | 136 | 114 | 163 |
| ONHF02 | 126 | 115 | 163 |
| ONHF03 | 126 | 115 | 163 |
| ONHF04 | 138 | 115 | 161 |
| ONHF05 | 136 | 115 | 163 |
| ONHF06 | 136 | 114 | 163 |
| ONHF07 | 136 | 115 | 161 |
| ONHF08 | 126 | 115 | 163 |
| ONHF09 | 136 | 115 | 161 |
| ONHF10 | 126 | 115 | 163 |
| ONHF11 | 136 | 115 | 169 |
| ONHF12 | 136 | 115 | 163 |
| ONHF13 | 140 | 115 | 163 |
| ONHF14 | 136 | 116 | 163 |
| ONHF15 | 138 | 115 | 169 |

| | | | |
|---|---|---|---|
| ONHF16 | 136 | 115 | 163 |
| ONHF17 | 138 | 115 | 169 |
| ONHF18 | 136 | 115 | 161 |
| ONHF19 | 136 | 114 | 163 |
| ONHF20 | 136 | 115 | 163 |
| ONGL01 | 136 | 115 | 161 |
| ONGL02 | 126 | 116 | 163 |
| ONGL03 | 138 | 115 | 163 |
| ONGL04 | 138 | 115 | 163 |
| ONGL05 | 136 | 116 | 161 |
| ONGL06 | 136 | 116 | 161 |
| ONGL07 | 138 | 115 | 161 |
| ONGL08 | 136 | 115 | 163 |
| ONGL09 | 138 | 115 | 163 |
| ONGL10 | 138 | 115 | 163 |
| ONGL11 | 136 | 115 | 165 |
| ONGL12 | 136 | 114 | 163 |
| ONGL13 | 126 | 115 | 163 |
| ONGL14 | 126 | 115 | 169 |
| ONGL15 | 136 | 114 | 163 |
| ONGL16 | 136 | 115 | 163 |
| ONGL17 | 138 | 115 | 159 |
| ONGL18 | 126 | 115 | 163 |
| ONGL19 | 136 | 115 | 163 |
| ONGL20 | 136 | 115 | 163 |
| ONMWL01 | 136 | 116 | 161 |
| ONMWL02 | 126 | 115 | 163 |
| ONMWL03 | 138 | 114 | 163 |
| ONMWL04 | 138 | 115 | 163 |
| ONMWL05 | 126 | 115 | 161 |
| ONMWL06 | 136 | 115 | 163 |
| ONMWL07 | 140 | 115 | 163 |
| ONMWL08 | 138 | 115 | 163 |
| ONMWL09 | 138 | 115 | 159 |
| ONMWL10 | 136 | 114 | 163 |
| ONMWL11 | 136 | 114 | 163 |
| ONMWL12 | 136 | 115 | 163 |
| ONMWL13 | 138 | 115 | 167 |
| ONMWL14 | 136 | 115 | 163 |
| ONMWL15 | 138 | 115 | 163 |
| ONMWL16 | 136 | 115 | 163 |
| ONMWL17 | 140 | 115 | 163 |
| ONMWL18 | 136 | 116 | 163 |
| ONMWL19 | 136 | 116 | 163 |
| ONMWL20 | 136 | 115 | 163 |
| ONRC01 | 126 | 115 | 163 |
| ONRC02 | 136 | 113 | 163 |
| ONRC03 | 136 | 114 | 163 |
| ONRC04 | 136 | 115 | 161 |

| | | | |
|---|---|---|---|
| ONRC05 | 126 | 115 | 163 |
| ONRC06 | 138 | 115 | 163 |
| ONRC07 | 126 | 115 | 163 |
| ONRC08 | 126 | 115 | 161 |
| ONRC09 | 136 | 114 | 163 |
| ONRC10 | 136 | 115 | 163 |
| ONRC11 | 126 | 115 | 163 |
| ONRC12 | 136 | 114 | 169 |
| ONRC13 | 138 | 115 | 163 |
| ONRC14 | 140 | 115 | 163 |
| ONRC15 | 138 | 115 | 163 |
| ONRC16 | 136 | 114 | 163 |
| ONRC17 | 136 | 115 | 163 |
| ONRC18 | 136 | 115 | 163 |
| ONRC19 | 136 | 116 | 163 |
| ONRC20 | 136 | 115 | 163 |
| ONWL01 | 140 | 115 | 163 |
| ONWL02 | 136 | 114 | 163 |
| ONWL03 | 136 | 115 | 161 |
| ONWL04 | 126 | 115 | 159 |
| ONWL05 | 126 | 115 | 163 |
| ONWL06 | 140 | 115 | 163 |
| ONWL07 | 136 | 114 | 163 |
| ONWL08 | 136 | 115 | 159 |
| ONWL09 | 138 | 115 | 163 |
| ONWL10 | 136 | 114 | 163 |
| ONWL11 | 138 | 115 | 163 |
| ONWL12 | 126 | 115 | 163 |
| ONWL13 | 136 | 116 | 163 |
| ONWL14 | 136 | 115 | 163 |
| ONWL15 | 136 | 116 | 161 |
| ONWL16 | 126 | 115 | 163 |
| ONWL17 | 136 | 115 | 163 |
| ONWL18 | 136 | 115 | 167 |
| ONWL19 | 136 | 116 | 163 |
| ONWL20 | 138 | 115 | 163 |
| ONWLW01 | 136 | 115 | 159 |
| ONWLW02 | 140 | 115 | 159 |
| ONWLW03 | 140 | 116 | 163 |
| ONWLW04 | 136 | 116 | 163 |
| ONWLW05 | 138 | 115 | 163 |
| ONWLW06 | 136 | 115 | 163 |
| ONWLW07 | 138 | 115 | 163 |
| ONWLW08 | 136 | 116 | 159 |
| ONWLW09 | 138 | 115 | 163 |
| ONWLW10 | 136 | 115 | 159 |
| ONWLW11 | 140 | 115 | 163 |
| ONWLW12 | 136 | 115 | 163 |
| ONWLW13 | 136 | 115 | 163 |

| | | | |
|---|---|---|---|
| ONWLW14 | 140 | 115 | 167 |
| ONWLW15 | 136 | 115 | 167 |
| ONWLW16 | 142 | 115 | 163 |
| ONWLW17 | 136 | 115 | 159 |
| ONWLW18 | 138 | 114 | 169 |
| ONWLW19 | 136 | 115 | 167 |
| ONWLW20 | 140 | 115 | 163 |
| ONTO01 | 138 | 115 | 163 |
| ONTO02 | 138 | 115 | 163 |
| ONTO03 | 140 | 115 | 161 |
| ONTO04 | 126 | 115 | 163 |
| ONTO05 | 136 | 116 | 163 |
| ONTO06 | 138 | 115 | 161 |
| ONTO07 | 136 | 114 | 163 |
| ONTO08 | 126 | 115 | 163 |
| ONTO09 | 126 | 115 | 161 |
| ONTO10 | 138 | 115 | 161 |
| ONTO11 | 140 | 115 | 161 |
| ONTO12 | 138 | 115 | 163 |
| ONTO13 | 136 | 115 | 163 |
| ONTO14 | 138 | 115 | 163 |
| ONTO15 | 138 | 115 | 163 |
| ONTO16 | 136 | 114 | 163 |
| ONTO17 | 140 | 115 | 159 |
| ONTO18 | 136 | 114 | 163 |
| ONTO19 | 136 | 114 | 163 |
| ONTO20 | 136 | 114 | 163 |
| MEEB01 | 136 | 113 | 163 |
| MEEB02 | 136 | 115 | 161 |
| MEEB03 | 136 | 115 | 167 |
| MEEB04 | 136 | 115 | 161 |
| MEEB05 | 136 | 115 | 161 |
| MEEB06 | 126 | 115 | 163 |
| MEEB07 | 136 | 113 | 163 |
| MEEB08 | 136 | 113 | 163 |
| MEEB09 | 136 | 115 | 161 |
| MEEB10 | 136 | 115 | 161 |
| MEEB11 | 126 | 114 | 163 |
| MEEB12 | 136 | 114 | 163 |
| MEEB13 | 136 | 115 | 167 |
| MEEB14 | 138 | 114 | 163 |
| MEEB15 | 136 | 113 | 163 |
| MEEB16 | 136 | 114 | 163 |
| MEEB17 | 136 | 115 | 163 |
| MEEB18 | 136 | 115 | 163 |
| MEEB19 | 136 | 115 | 163 |
| MEEB20 | 136 | 115 | 163 |
| MEBSP01 | 136 | 115 | 163 |
| MEBSP02 | 136 | 115 | 163 |

| | | | |
|---|---|---|---|
| MEBSP03 | 136 | 115 | 161 |
| MEBSP04 | 126 | 115 | 161 |
| MEBSP05 | 136 | 115 | 165 |
| MEBSP06 | 136 | 115 | 163 |
| MEBSP07 | 136 | 115 | 163 |
| MEBSP08 | 136 | 115 | 163 |
| MEBSP09 | 136 | 115 | 161 |
| MEBSP10 | 136 | 115 | 167 |
| MEBSP11 | 136 | 115 | 163 |
| MEBSP12 | 136 | 115 | 163 |
| MEBSP13 | 136 | 115 | 163 |
| MEBSP14 | 136 | 115 | 165 |
| MEBSP15 | 136 | 115 | 163 |
| MEBSP16 | 136 | 115 | 163 |
| MEBSP17 | 136 | 115 | 161 |
| MEBSP18 | 136 | 115 | 163 |
| MEBSP19 | 136 | 115 | 163 |
| MEBSP20 | 136 | 115 | 163 |
| MASB01 | 136 | 115 | 165 |
| MASB02 | 138 | 115 | 161 |
| MASB03 | 136 | 114 | 165 |
| MASB04 | 136 | 113 | 163 |
| MASB05 | 126 | 115 | 161 |
| MASB06 | 126 | 115 | 161 |
| MASB07 | 126 | 115 | 161 |
| MASB08 | 136 | 115 | 165 |
| MASB09 | 136 | 113 | 163 |
| MASB10 | 136 | 113 | 163 |
| MASB11 | 126 | 115 | 163 |
| MASB12 | 136 | 113 | 161 |
| MASB13 | 136 | 114 | 163 |
| MASB14 | 136 | 113 | 163 |
| MASB15 | 126 | 115 | 165 |
| MASB16 | 136 | 115 | 165 |
| MASB17 | 126 | 115 | 163 |
| MASB18 | 126 | 115 | 163 |
| MASB19 | 136 | 115 | 165 |
| MASB20 | 136 | 113 | 163 |
| NYCM01 | 136 | 115 | 163 |
| NYCM02 | 126 | 114 | 161 |
| NYCM03 | 138 | 115 | 167 |
| NYCM04 | 136 | 115 | 163 |
| NYCM05 | 126 | 114 | 161 |
| NYCM06 | 126 | 115 | 163 |
| NYCM07 | 138 | 115 | 163 |
| NYCM08 | 136 | 115 | 161 |
| NYCM09 | 136 | 115 | 161 |
| NYCM10 | 138 | 115 | 165 |
| NYCM11 | 136 | 114 | 163 |

| | | | |
|---|---|---|---|
| NYCM12 | 138 | 115 | 163 |
| NYCM13 | 136 | 115 | 165 |
| NYCM14 | 136 | 115 | 163 |
| NYCM15 | 136 | 114 | 165 |
| NYCM16 | 126 | 115 | 163 |
| NYCM17 | 138 | 113 | 163 |
| NYCM18 | 138 | 115 | 163 |
| NYCM19 | 136 | 115 | 163 |
| NYCM20 | 136 | 114 | 163 |
| NHEWP01 | 136 | 113 | 163 |
| NHEWP02 | 138 | 115 | 163 |
| NHEWP03 | 136 | 115 | 161 |
| NHEWP04 | 136 | 114 | 163 |
| NHEWP05 | 126 | 115 | 163 |
| NHEWP06 | 126 | 115 | 161 |
| NHEWP07 | 136 | 114 | 163 |
| NHEWP08 | 136 | 115 | 165 |
| NHEWP09 | 136 | 115 | 161 |
| NHEWP10 | 126 | 115 | 163 |
| NHEWP11 | 136 | 114 | 163 |
| NHEWP12 | 126 | 115 | 163 |
| NHEWP13 | 136 | 115 | 163 |
| NHEWP14 | 136 | 113 | 165 |
| NHEWP15 | 136 | 115 | 163 |
| NHEWP16 | 136 | 115 | 163 |
| NHEWP17 | 136 | 113 | 163 |
| NHEWP18 | 126 | 115 | 163 |
| NHEWP19 | 136 | 115 | 163 |
| NHEWP20 | 136 | 115 | 163 |
| MNWL01 | 136 | 115 | 163 |
| MNWL02 | 140 | 115 | 159 |
| MNWL03 | 140 | 116 | 161 |
| MNWL04 | 136 | 115 | 163 |
| MNWL05 | 138 | 115 | 163 |
| MNWL06 | 138 | 116 | 159 |
| MNWL07 | 138 | 115 | 177 |
| MNWL08 | 136 | 115 | 163 |
| MNWL09 | 140 | 115 | 163 |
| MNWL10 | 140 | 115 | 159 |
| MNWL11 | 136 | 114 | 163 |
| MNWL12 | 138 | 115 | 159 |
| MNWL13 | 140 | 116 | 159 |
| MNWL14 | 142 | 115 | 163 |
| MNWL15 | 136 | 116 | 163 |
| MNWL16 | 138 | 115 | 165 |
| MNWL17 | 136 | 116 | 159 |
| MNWL18 | 140 | 115 | 167 |
| MNWL19 | 136 | 115 | 163 |
| MNWL20 | 136 | 116 | 159 |

| | | | |
|---|---|---|---|
| MNBL01 | 140 | 115 | 151 |
| MNBL02 | 140 | 115 | 159 |
| MNBL03 | 136 | 114 | 163 |
| MNBL04 | 138 | 115 | 161 |
| MNBL05 | 136 | 114 | 163 |
| MNBL06 | 138 | 115 | 159 |
| MNBL07 | 138 | 116 | 163 |
| MNBL08 | 136 | 115 | 161 |
| MNBL09 | 140 | 115 | 159 |
| MNBL10 | 140 | 115 | 159 |
| MNBL11 | 136 | 114 | 163 |
| MNBL12 | 138 | 116 | 161 |
| MNBL13 | 140 | 115 | 161 |
| MNBL14 | 142 | 115 | 159 |
| MNBL15 | 138 | 115 | 161 |
| MNBL16 | 136 | 115 | 159 |
| MNBL17 | 136 | 115 | 159 |
| MNBL18 | 140 | 115 | 161 |
| MNBL19 | 136 | 115 | 161 |
| MNBL20 | 138 | 116 | 163 |
| PAOL01 | 136 | 115 | 163 |
| PAOL02 | 136 | 115 | 163 |
| PAOL03 | 136 | 114 | 161 |
| PAOL04 | 126 | 114 | 167 |
| PAOL05 | 136 | 114 | 161 |
| PAOL06 | 136 | 115 | 161 |
| PAOL07 | 126 | 114 | 161 |
| PAOL08 | 136 | 115 | 163 |
| PAOL09 | 138 | 115 | 169 |
| PAOL10 | 136 | 115 | 167 |
| PAOL11 | 126 | 115 | 163 |
| PAOL12 | 136 | 114 | 167 |
| PAOL13 | 126 | 115 | 163 |
| PAOL14 | 136 | 115 | 169 |
| PAOL15 | 138 | 114 | 163 |
| PAOL16 | 136 | 113 | 163 |
| PAOL17 | 136 | 115 | 163 |
| PAOL18 | 136 | 115 | 163 |
| PAOL19 | 136 | 115 | 163 |
| PAOL20 | 136 | 115 | 163 |
| VABS01 | 138 | 115 | 161 |
| VABS02 | 136 | 115 | 161 |
| VABS03 | 136 | 114 | 163 |
| VABS04 | 138 | 114 | 163 |
| VABS05 | 136 | 115 | 167 |
| VABS06 | 126 | 115 | 163 |
| VABS07 | 136 | 115 | 167 |
| VABS08 | 138 | 115 | 161 |
| VABS09 | 136 | 114 | 163 |

| | | | |
|---|---|---|---|
| VABS10 | 138 | 116 | 169 |
| VABS11 | 136 | 115 | 163 |
| VABS12 | 126 | 115 | 163 |
| VABS13 | 136 | 113 | 161 |
| VABS14 | 136 | 115 | 163 |
| VABS15 | 128 | 115 | 161 |
| VABS16 | 136 | 115 | 161 |
| VABS17 | 136 | 114 | 163 |
| VABS18 | 138 | 115 | 163 |
| VABS19 | 136 | 114 | 163 |
| VABS20 | 136 | 115 | 163 |
| NCAV01 | 136 | 115 | 161 |
| NCAV02 | 140 | 115 | 167 |
| NCAV03 | 136 | 116 | 163 |
| NCAV04 | 126 | 114 | 163 |
| NCAV05 | 126 | 115 | 161 |
| NCAV06 | 136 | 114 | 161 |
| NCAV07 | 140 | 115 | 161 |
| NCAV08 | 136 | 116 | 163 |
| NCAV09 | 138 | 115 | 167 |
| NCAV10 | 136 | 115 | 169 |
| NCAV11 | 136 | 113 | 163 |
| NCAV12 | 140 | 115 | 163 |
| NCAV13 | 136 | 115 | 161 |
| NCAV14 | 136 | 113 | 163 |
| NCAV15 | 126 | 115 | 163 |
| NCAV16 | 136 | 114 | 161 |
| NCAV17 | 136 | 116 | 163 |
| NCAV18 | 138 | 115 | 169 |
| NCAV19 | 136 | 115 | 163 |
| NCAV20 | 136 | 115 | 163 |
